# Lewis acid-promoted direct synthesis of isoxazole derivatives

**DOI:** 10.3762/bjoc.19.113

**Published:** 2023-10-16

**Authors:** Dengxu Qiu, Chenhui Jiang, Pan Gao, Yu Yuan

**Affiliations:** 1 College of Chemistry and Chemical Engineering, Yangzhou University, Yangzhou 225002, China

**Keywords:** aluminum trichloride, Lewis acid, isoxazole derivatives, sodium nitrite, transition metals

## Abstract

Isoxazole derivatives were synthesized via a one-pot method utilizing 2-methylquinoline derivatives as template substrates, sodium nitrite as a nitrogen-oxygen source, and solely using aluminum trichloride as the additive. This approach circumvents the need for costly or highly toxic transition metals and presents a novel pathway for the synthesis of isoxazole derivatives.

## Introduction

**Figure 1:**
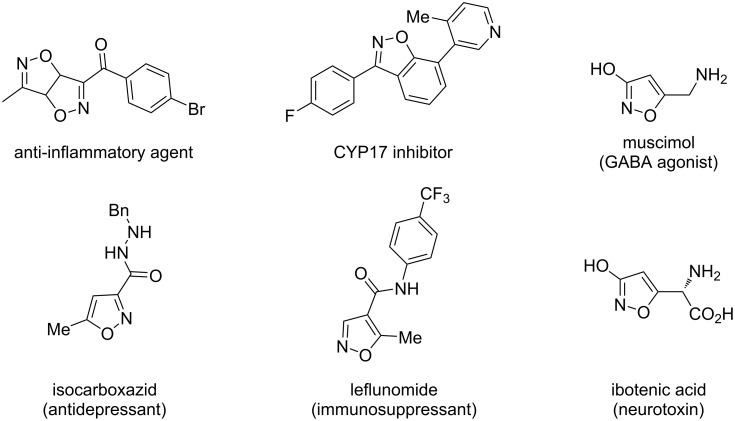
Natural products and drug molecules containing isoxazole moieties.

**Scheme 1:**
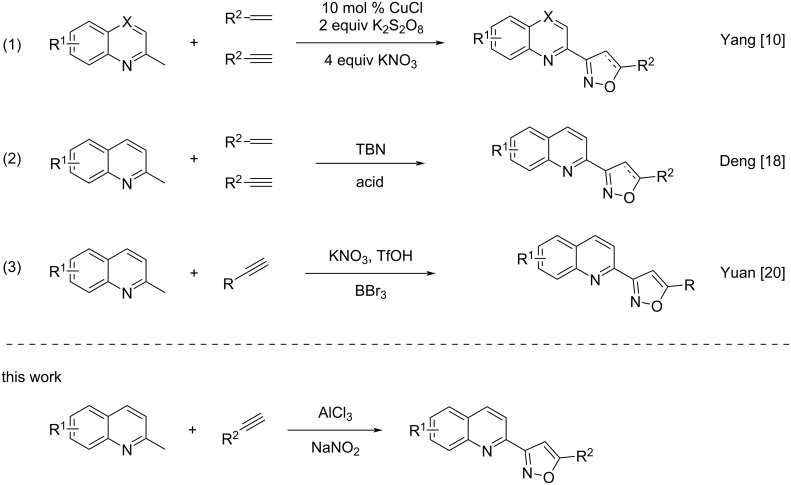
Traditional methods for the synthesis of isoxazoles and the current approach.

The isoxazole derivatives not only exist in many natural products [1–3] and pharmaceutical intermediates [4–7], but also have great application values in organic synthesis [8–9] (Figure 1). In the past decades, many methods have been developed to prepare isoxazole derivatives [10–13]. However, most of the starting materials for these methods are oximes and hydroximinoyl chlorides [4,13–15]. Recently, the sp^3^ C–H bond functional group transformation of 2-methylquinoline derivatives into isoxazole derivatives has been reported [16]. In 2015, Yang’s group [10,17] reported the copper-catalyzed conversion of methylarenes into isoxazole derivatives with KNO_3_ as the source of nitrile oxide (Scheme 1, reaction 1). In 2019, Deng’s group [18] developed a three-component synthesis method of isoxazole derivatives using TBN as nitrogen source (Scheme 1, reaction 2). In 2017, Xu and co-workers [19] developed a copper-mediated annulation reaction to synthesize isoxazoles from two different alkynes. In fact, most methods mostly used highly toxic transition-metal catalysts such as copper metals. In order to develop cheaper and more environmentally friendly catalysts, our laboratory recently developed an alternative approach to the synthesis of isooxazoles starting from 2-methylquinoline and alkynes mediated by Brønsted acids in good yields (Scheme 1, reaction 3) [20].

The utilization of main element metal aluminum salts in organic synthesis holds significant potential due to their cost-effectiveness as compared to heavy metals. This renders them highly valuable for various applications in the field. Herein, we successfully developed a method that uses sodium nitrite as the source of nitrile oxide, and only applies aluminium trichloride as the Lewis acid to realize the sp^3^ C–H-bond activation of nitrogen heterocycles to synthesize isoxazole derivatives.

## Results and Discussion

At the outset of this study, we chose the reaction of 2-methylquinoline (**2a**) with phenylacetylene (**1a**) in the presence of AlCl_3_ (3 equiv) and sodium nitrite (10 equiv) in DMAc at 90 °C under a nitrogen atmosphere. The desired isoxazole product **3a** was isolated in 92% yield (Table 1, entry 1). It was found that no product was formed in the absence of AlCl_3_ and 64% yield were obtained when the reaction was performed using 2 equiv AlCl_3_ (Table 1, entries 2 and 3). When 5 equiv sodium nitrite were used, the corresponding yield was also decreased (Table 1, entry 4). Other Lewis acids employed in the reaction were less effective than AlCl_3_ (Table 1, entries 5–7). Furthermore, solvent screening showed that DMAc was the best reaction medium for this cycloaddition compared with DMSO and DMF (Table 1, entries 8 and 9). The reaction yield was decreased to 21% when increasing the temperature to 140 °C under standard conditions (Table 1, entry 10). Finally, the nitrogen atmosphere was essential since the yield substantially decreased under air atmosphere (Table 1, entry 11).

**Table 1:** Optimization of reaction conditions for the synthesis of isoxazoles^a^.

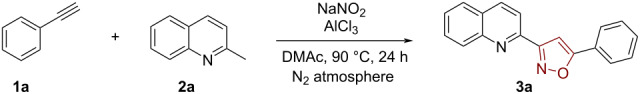		

Entry	Variation from standard conditions	Yield (%)^b^

1	none	92
2	without AlCl_3_	n.r.
3	2 equiv of AlCl_3_	64
4	5 equiv of NaNO_2_	77
5	FeCl_3_ instead of AlCl_3_	55
6	TiCl_4_ instead of AlCl_3_	67
7	BF_3_ instead of AlCl_3_	n.r.
8	DMF instead of DMAc	72
9	DMSO instead of DMAc	n.r.
10	at 140 °C	21
11	under air	26

^a^Standard reaction conditions: **1a** (0.1 mmol, 1 equiv), **2a** (0.2 mmol, 2 equiv), AlCl_3_ (0.3 mmol, 3 equiv), NaNO_2_ (1 mmol, 10.0 equiv), DMAc (1.0 mL), N_2_ atmosphere, 90 °C, 24 h. ^b^Isolated yield; n.r., no reaction.

**Scheme 2:**
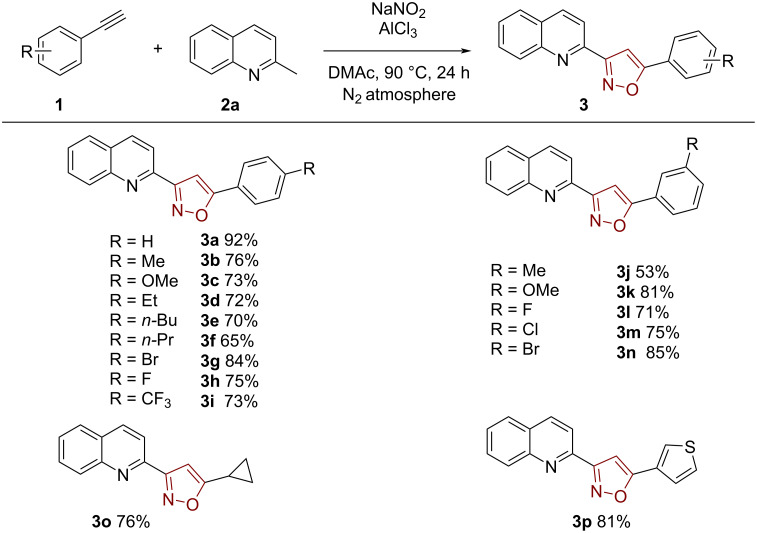
Reaction scope of alkynes. Conditions: **1** (0.1 mmol, 1 equiv), **2a** (0.2 mmol, 2 equiv), AlCl_3_ (0.3 mmol, 3 equiv), NaNO_2_ (1 mmol, 10.0 equiv), DMAc (1.0 mL), N_2_ atmosphere, 90 °C, 24 h.

**Figure 2:**
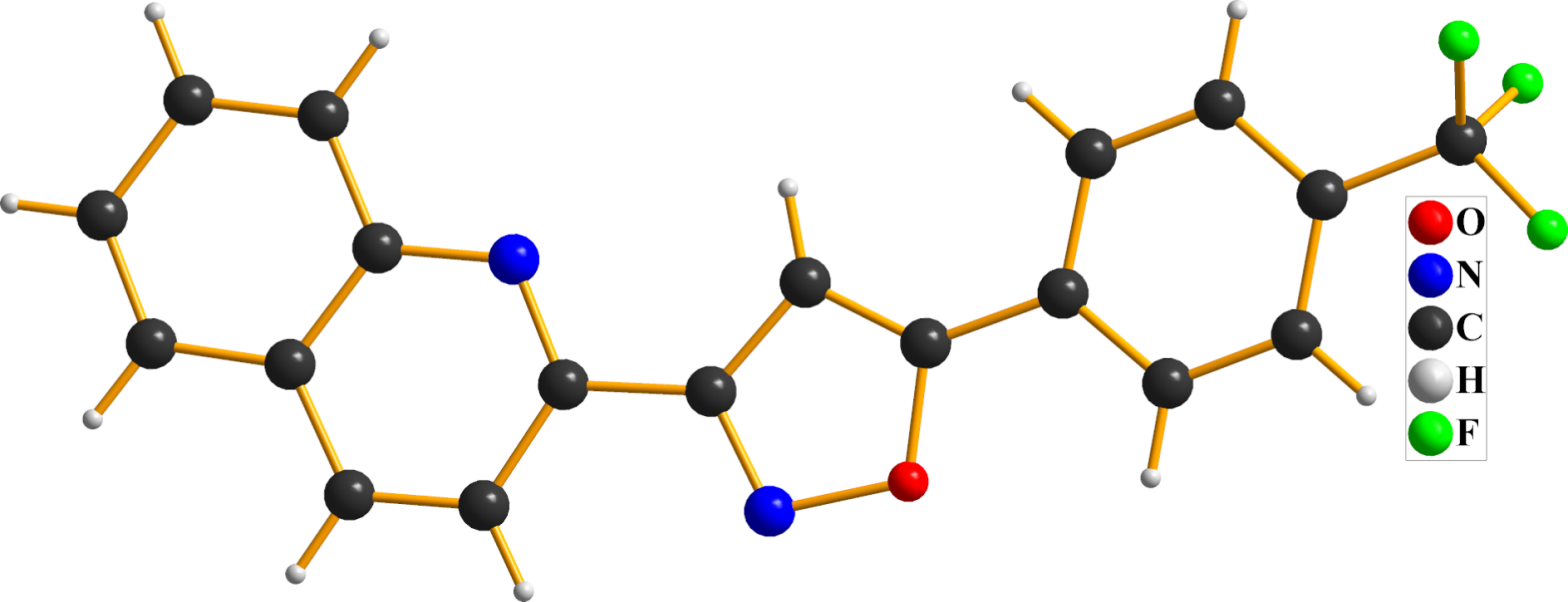
Crystal structure of **3i**.

With the optimal reaction conditions in hand, various alkynes were examined as dipolarophiles (Scheme 2). A range of functional groups were tolerated in this reaction, such as alkyl, methoxy, halo, and heterocycles. It was found that electron-deficient groups in the phenyl ring (**3g**–**i**) were more beneficial to the reaction outcome than electron-rich groups in the phenyl ring (**3a**–**f**). The crystal structure of product **3i** is shown in Figure 2. Also, substituents in different positions of the phenyl ring in acetylene **1** smoothly reacted with NaNO_2_ under the reaction conditions affording the products in good to excellent yields, which showed that the steric hindrance has little effect on the reaction (**3j**–**n**). Furthermore, some heteroaromatic and aliphatic alkynes were also utilized, and the corresponding products **3o** and **3p** were isolated in good yields. We also tried 1,2-diphenylethyne as sustrate, which is an internal alkyne instead of a terminal alkyne, but no desired product was obtained.

**Scheme 3:**
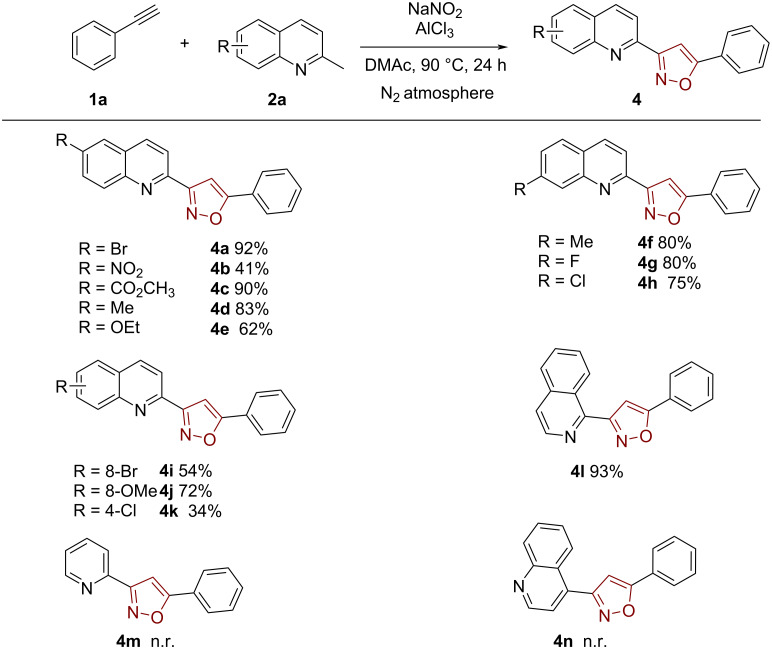
Reaction substrate scope of quinolines. Conditions: **1a** (0.1 mmol, 1 equiv), **2** (0.2 mmol, 2 equiv), AlCl_3_ (0.3 mmol, 3 equiv), NaNO_2_ (1 mmol, 10.0 equiv), DMAc (1.0 mL), N_2_ atmosphere, 90 °C, 24 h.

Next, we explored the substrate scope of 2-methylquinolines under the standard conditions. 2-Methylquinoline bearing different substituents at various positions gave the corresponding products with moderate to good yields (Scheme 3). It was observed that 2-methylquinoline with electron-deficient functional groups afforded the corresponding products in excellent yields of up to 92% (**4a**–**c**). Likewise, 2-methylquinoline substituted with electron-rich functional groups were suitable substrates and achieved good results (**4d** and **4e**). Fortunately, various functional groups in different positions were also tolerated in the reaction (**4f**–**k**). Moreover, this reaction could be carried out with 1-methylisoquinoline as substrate, which afforded product **4l** in 93% yield. Besides, we also tried 2-methylpyridine and 4-methylquinoline as substrates, but no reaction was detected under the standard conditions (**4m** and **4n**). In addition, there was no product formed when 2-formylquinoline was used as the substrate.

**Scheme 4:**
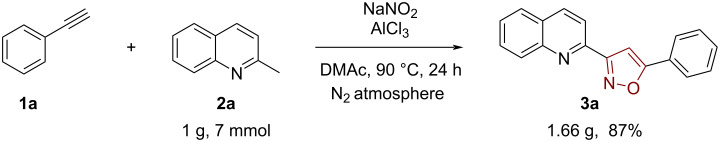
Gram scale reaction.

To further demonstrate the synthetic versatility of this developed method, we carried out the reaction in a gram scale. It was found that the desired product could be obtained in 87% yield (Scheme 4).

Next, some control experiments were carried out to study the reaction mechanism. We found that the reaction of compound **3a** could not be inhibited by TEMPO and BHT under the standard conditions. Therefore, it is assumed that the reaction is not a free radical reaction.

**Scheme 5:**
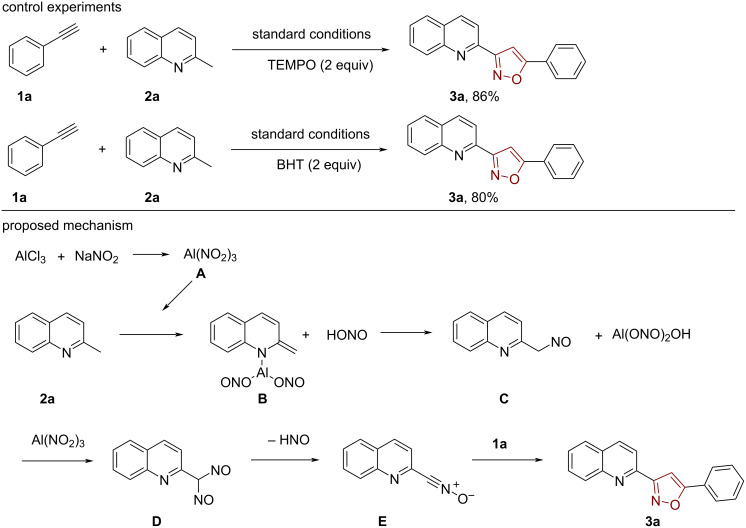
Control experiments and possible reaction mechanism.

Based on the control experiments and previous literature [21], we propose the following possible mechanism, which is shown in Scheme 5. Aluminum trichloride reacts with sodium nitrite to form an intermediate aluminum complex **A**, which is further complexed with the starting material **2a** to generate intermediate **B** and HONO [22]. Then, the intermediate **B** conjugates with HONO to generate intermediate **C** [22]. Next, the intermediate **D** is produced by the same progress. The intermediate **D** then undergoes elimination of nitroxylic acid to produce nitrile oxide **E** [23], which can be converted to the desired isoxazole with **1a** through a 1,3-dipolar cycloaddition.

## Conclusion

In conclusion, we have developed an efficient and concise synthesis of isoxazole nitrogen heterocycles by direct C–H-bond activation of methyl heteroaromatics. The method avoids using toxic transition metals and provides a new way to synthesize isoxazole molecules. Further related transformations of products and application of this method are currently developed in our laboratory.

## Experimental

Representative procedure for the synthesis of compound **3a**. To a flame-dried 15 mL Schlenk tube filled with nitrogen, 2-methylquinoline (**2a**, 28.6 mg, 0.2 mmol), phenylacetylene (**1a**, 10.2 mg, 0.1 mmol), AlCl_3_ (40.0 mg, 0.3 mmol), sodium nitrite (35.0 mg, 1.0 mmol), and absolute dry DMAc (1.0 mL) were added under nitrogen. The formed mixture was stirred at 90 °C under nitrogen for 24 h with TLC monitoring. Upon completion, the solution was cooled to room temperature and the solvent was removed under vacuum directly. The crude residue was purified by column chromatography on silica gel (ethyl acetate/petroleum ether 40:1) to afford product **3a** with 87% yield.

## Supporting Information

File 1 Characterization data and copies of spectra.
