# Supplementary material for: Lewis acid-promoted direct synthesis of isoxazole derivatives
Source: Beilstein J Org Chem. 2023 Oct 16;19:1562–7. doi: 10.3762/bjoc.19.113 (PMC10616705; doi:10.3762/bjoc.19.113)

BEILSTEIN JOURNAL OF ORGANIC CHEMISTRY

Supporting Information

for

Lewis acid-promoted direct synthesis of isoxazole derivatives

Dengxu Qiu, Chenhui Jiang, Pan Gao and Yu Yuan

Beilstein J. Org. Chem. **2023,** *19,* 1562–1567. doi:10.3762/bjoc.19.113

Characterization data and copies of spectra

License and Terms: This is a supporting information file under the terms of the Creative Commons Attribution License (https://creativecommons.org/licenses/by/4.0). Please note that the reuse, redistribution and reproduction in particular requires that the author(s) and source are credited and that individual graphics may be subject to special legal provisions.
The license is subject to the *Beilstein Journal of Organic Chemistry* terms and conditions: (https://www.beilstein-journals.org/bjoc/terms)

General information:

Unless otherwise noted, all reagents and solvents were obtained from commercial suppliers and used without further purification. ^{1}H NMR, ^{13}C NMR, and ^{19}F NMR spectra were obtained with an Agilent Technologies 400 or 500 spectrometer in $CDCl_3$ or DMSO-d_6 with TMS as an internal standard. Mass spectra were obtained on a Bruker Dalton maxis instrument. All reactions were carried out under a nitrogen atmosphere. All reactions under standard conditions were monitored by thin-layer chromatography (TLC) on gel F254 plates. Flash column chromatography was carried out using 300–400 mesh silica gel at medium pressure.

Gram-scale synthesis of 3a

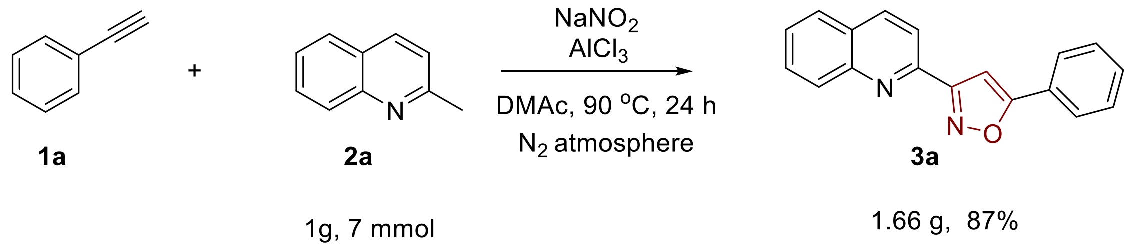

To a flame-dried 100 mL Schlenk tube filled with nitrogen, 2-methylquinoline (**2a**, 1.0 g, 7 mmol), phenylacetylene (**1a**, 0.36 g, 3.5 mmol), $AlCl_3$ (1.4 g, 10.5 mmol), sodium nitrite (2.4 g, 35 mmol), and absolute dry DMAc (30 mL) were added under nitrogen. The formed mixture was stirred at 90 °C under nitrogen for 24 h and monitored by TLC. After work-up, 1.66 g (87%) of product **3a** were obtained.

Characterization of the products

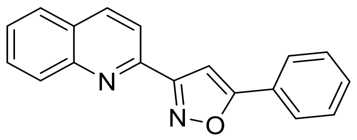

5-Phenyl-3-(quinolin-2-yl)isoxazole (3a)*[1,2,3]*. Yield: 92%. yellow solid. Mp: 128-130℃.

^{1}H NMR (400 MHz, CDCl$_3$): δ 8.30 – 8.15 (m, 3H), 7.93 – 7.82 (m, 3H), 7.76 (ddd, J = 8.5, 6.9, 1.5 Hz, 1H), 7.58 (ddd, J = 8.1, 6.9, 1.2 Hz, 1H), 7.53 – 7.45 (m, 3H), 7.39 (s, 1H).

^{13}C NMR (101 MHz, CDCl$_3$): δ 170.6, 164.2, 148.7, 148.0, 136.9, 130.3, 129.9, 129.7, 129.0, 128.4, 127.7, 127.5, 127.3, 125.9, 119.1, 98.6.

HRMS (ESI) m/z: $[M+H]^+$calcd. for: $C_{18}H_{13}N_2O^+$: 273.1022; found 273.1027.

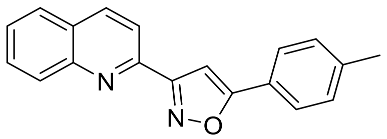

3-(Quinolin-2-yl)-5-(p-tolyl)isoxazole (3b)* [1,2]*. Yield: 76%. yellow solid. Mp: 150-152℃.

^{1}H NMR (400 MHz, CDCl$_3$): δ 8.28 – 8.16 (m, 3H), 7.86 (dd, J = 7.8, 1.3 Hz, 1H), 7.81 – 7.73 (m, 3H), 7.59 (ddd, J = 8.1, 6.9, 1.2 Hz, 1H), 7.34 (s, 1H), 7.30 (dt, J = 7.8, 0.8 Hz, 2H), 2.42 (s, 3H).

^{13}C NMR (101 MHz, CDCl$_3$): δ 170.9, 164.1, 148.8, 148.0, 140.6, 136.9, 129.9, 129.7, 129.7, 128.4, 127.7, 127.3, 125.8, 124.8, 119.1, 98.0, 21.5.

HRMS (ESI) m/z: $[M+H]^+$ calcd. for: $C_{19}H_{15}N_2O^+$: 287.1179; found 287.1182.

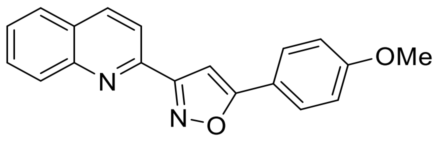

***5-(4-Methoxyphenyl)-3-(quinolin-2-yl)isoxazole (3c)* [1,2].** Yield: 73%. yellow solid. Mp: 140-142℃.

^{1}H NMR (400 MHz, CDCl$_3$): δ 8.3 – 8.15 (m, 3H), 7.9 – 7.81 (m, 3H), 7.78 – 7.73 (m, 1H), 7.71 – 7.66 (m, 1H), 7.60 – 7.55 (m, 1H), 7.3 (s, 1H), 7.0 – 6.9 (m, 2H), 3.9 (s, 3H).

^{13}C NMR (101 MHz, CDCl$_3$): δ 170.6, 164.1, 161.1, 148.8, 148.2, 148.0, 137.5, 136.9, 131.3, 130.0, 129.9, 129.7, 129.5, 128.4, 127.8, 127.7, 127.5, 127.3, 123.3, 120.3, 119.1, 114.4, 97.2, 55.4.

HRMS (ESI) m/z: [M+Na]$^{+}$ calcd. for: $C_{19}H_{14}N_2O_2^{+}$: 325.0953; found 325.0943.

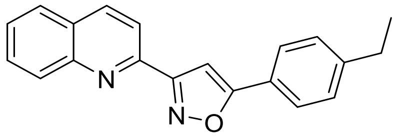

***5-(4-Ethylphenyl)-3-(quinolin-2-yl)isoxazole (3d)* [1,2].** Yield: 72%. yellow solid. Mp:112-114℃.

^{1}H NMR (400 MHz, CDCl$_3$): δ 8.29 – 8.16 (m, 3H), 7.89 – 7.81 (m, 3H), 7.76 (td, J = 6.9, 1.6 Hz, 1H), 7.62 – 7.56 (m, 1H), 7.37 – 7.30 (m, 3H), 2.72 (q, J = 7.7 Hz, 2H), 1.29 (t, J = 7.6 Hz, 3H).

^{13}C NMR (101 MHz, CDCl$_3$): δ 170.9, 164.1, 148.8, 148.0, 146.8, 136.9, 129.9, 129.7, 128.5, 128.4, 127.7, 127.3, 125.9, 125.0, 119.1, 98.0, 28.8, 15.3.

HRMS (ESI) m/z: [M+H]$^{+}$ calcd. for: $C_{20}H_{17}N_2O^{+}$: 301.1335; found 301.1338.

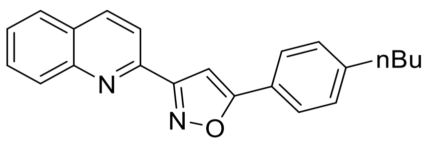

***5-(4-Butylphenyl)-3-(quinolin-2-yl)isoxazole (3e)* [1].** Yield: 70%. yellow solid. Mp: 58-61℃.

^{1}H NMR (400 MHz, CDCl$_3$): δ 8.28 – 8.15 (m, 3H), 7.86 (d, J = 8.1 Hz, 1H), 7.81 (dd, J = 8.1, 1.4 Hz, 2H), 7.78 – 7.74 (m, 1H), 7.61 – 7.56 (m, 1H), 7.34 (d, J = 1.3 Hz, 1H), 7.33 – 7.29 (m, 2H), 2.67 (t, J = 7.8 Hz, 2H), 1.67 – 1.61 (m, 2H), 1.38 (q, J = 7.0 Hz, 2H), 0.95 (td, J = 7.3, 1.3 Hz, 3H).

^{13}C NMR (101 MHz, CDCl$_3$): δ 170.9, 164.1, 148.8, 148.0, 145.6, 136.9, 129.9, 129.7, 129.2, 129.1, 128.4, 127.7, 127.3, 125.9, 124.9, 119.2, 98.0, 35.6, 33.4, 22.3, 13.9.

HRMS (ESI) m/z: [M+H]$^+$calcd. for: $C_{20}H_{22}N_2NaO^+$; 351.1473, found 351.1463.

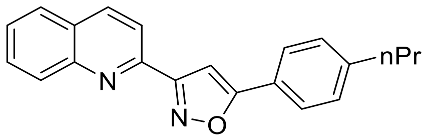

***5-(4-Propylphenyl)-3-(quinolin-2-yl)isoxazole (3f)* [2].** Yield: 65%. yellow solid. Mp: 88-90℃.

^{1}H NMR (400 MHz, CDCl$_3$): δ 8.28 – 8.16 (m, 3H), 7.86 (d, J = 8.2 Hz, 1H), 7.81 (d, J = 8.2 Hz, 2H), 7.78 – 7.74 (m, 1H), 7.58 (ddd, J = 8.1, 6.9, 1.2 Hz, 1H), 7.34 (s, 1H), 7.33 – 7.29 (m, 2H), 2.65 (t, 2H), 1.69 (dd, J = 7.5 Hz, 2H), 0.97 (t, J = 7.3 Hz, 3H).

^{13}C NMR (101 MHz, CDCl$_3$): δ 170.9, 164.1, 148.8, 148.0, 145.3, 136.9, 129.9, 129.7, 129.1, 128.4, 127.7, 127.3, 125.9, 125.0, 119.2, 98.0, 38.0, 24.3, 13.8.

HRMS (ESI) m/z: [M+H]$^+$calcd. for: $C_{21}H_{19}N_2O^+$: 315.1492; found 315.1499.

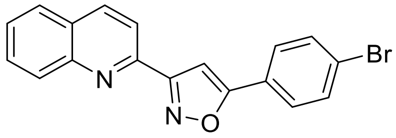

***5-(4-Bromophenyl)-3-(quinolin-2-yl)isoxazole (3g)* [2].** Yield: 84%. yellow solid. Mp: 200-202℃.

^{1}H NMR (400 MHz, CDCl$_3$): δ 8.27 – 8.13 (m, 3H), 7.87 – 7.82 (m, 1H), 7.78 – 7.70 (m, 3H), 7.64 – 7.55 (m, 3H), 7.37 (d, J = 1.6 Hz, 1H).

^{13}C NMR (101 MHz, CDCl$_3$): δ 169.5, 164.2, 148.4, 148.0, 137.0, 132.3, 130.0, 129.7, 128.4, 127.7, 127.4, 127.3, 126.3, 124.6, 119.0, 99.1, 99.0.

HRMS (ESI) m/z: [M+H]$^+$calcd. for: $C_{18}H_{12}BrN_2O^+$: 351.0128; found 351.0133.

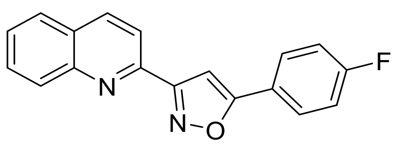

***5-(4-Fluorophenyl)-3-(quinolin-2-yl)isoxazole (3h)* [1,2].** Yield: 75%. white solid. Mp: 164-166℃.

^{1}H NMR (400 MHz, CDCl$_3$): δ 8.27 – 8.14 (m, 3H), 7.90 – 7.83 (m, 3H), 7.76 (t, J = 8.4, 6.9, 1.4 Hz, 1H), 7.58 (t, J = 8.1, 6.9, 1.2 Hz, 1H), 7.33 (s, 1H), 7.18 (t, J = 8.6 Hz, 2H).

^{13}C NMR (101 MHz, CDCl$_3$): δ 169.6, 165.0, 164.6 (d, J = 250.0 Hz), 148.5, 148.0, 137.0, 130.0, 129.7, 128.4, 127.9 (d, J = 9.0 Hz), 127.7, 127.3, 123.8 (d, J=3.0Hz), 119.1, 116.3 (d, J = 23.0 Hz), 98.4.

^{19}F NMR (471 MHz, CDCl$_3$): δ -109.4.

HRMS (ESI) m/z: [M+H]$^+$calcd. for: $C_{18}H_{12}FN_2O^+$: 291.0928; found 291.0932.

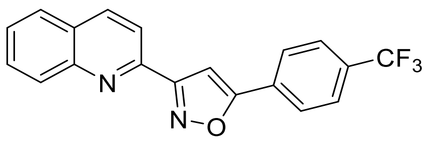

***3-(Quinolin-2-yl)-5-(4-(trifluoromethyl)phenyl)isoxazole (3i)* [1].** Yield: 73%. yellow solid. Mp: 172-175℃.

^{1}H NMR (400 MHz, CDCl$_3$): δ 8.22 – 8.11 (m, 3H), 7.93 (d, J = 8.1 Hz, 2H), 7.81 (d, J = 8.2 Hz, 1H), 7.76 – 7.67 (m, 3H), 7.59 – 7.52 (m, 1H), 7.44 (s, 1H).

^{13}C NMR (101 MHz, CDCl$_3$): δ 168.8, 164.3, 148.2, 148.0, 136.9, 131.7 (q, J=32.0 Hz), 130.4, 130.0, 129.7, 128.4, 127.7, 127.4, 126.0, 125.9, 123.7 (q, J=271.0 Hz), 118.9, 100.1.

^{19}F NMR (471 MHz, CDCl3): δ -62.9.

HRMS (ESI) m/z: [M+H]$^+$calcd. for: $C_{19}H_{12}F_3N_2O^+$: 341.0902; found 341.0908.

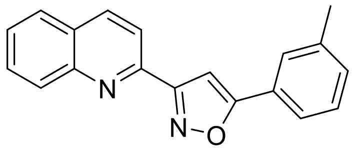

***3-(Quinolin-2-yl)-5-(m-tolyl)isoxazole (3j)* [1,2].** Yield: 53%. Yellow solid. Mp: 109-111℃.

^{1}H NMR (400 MHz, CDCl$_3$): δ 8.29 – 8.15 (m, 3H), 7.87 (dd, J = 8.1, 1.5 Hz, 1H), 7.79 – 7.69 (m, 3H), 7.59 (t, *J* = 8.1, 6.8, 1.2 Hz, 1H), 7.41 – 7.36 (m, 2H), 7.28 (d, J = 7.6 Hz, 1H), 2.45 (s, 3H).

^{13}C NMR (101 MHz, CDCl$_3$): δ 170.8, 164.1, 148.7, 148.1, 138.8, 136.9, 131.1, 129.9, 129.7, 128.9, 128.4, 127.7, 127.4, 127.3, 126.5, 123.1, 119.1, 98.5, 21.5.

HRMS (ESI) m/z: [M+H]$^+$ calcd. for: $C_{19}H_{15}N_2O^+$: 287.1179; found 287.1188.

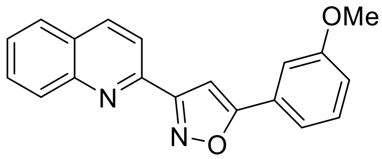

***5-(3-Methoxyphenyl)-3-(quinolin-2-yl)isoxazole (3k)* [1].** Yield: 81%. yellow solid. Mp: 104-106℃.

^{1}H NMR (400 MHz, CDCl$_3$): δ 8.29 – 8.16 (m, 3H), 7.86 (dd, J = 8.1, 1.5 Hz, 1H), 7.79 – 7.71 (m, 1H), 7.61 – 7.55 (m, 1H), 7.48 (dt, J = 7.6, 1.3 Hz, 1H), 7.45 – 7.35 (m, 3H), 7.03 – 6.98 (m, 1H), 3.89 (s, 3H).

^{13}C NMR (101 MHz, CDCl$_3$): δ 170.5, 164.2, 160.0, 148.6, 148.0, 136.9, 130.2, 129.9, 129.7, 128.6, 128.4, 127.7, 127.3, 119.1, 118.4, 116.4, 110.9, 98.9, 55.4, 55.4.

HRMS (ESI) m/z: [M+H]$^+$ calcd. for: $C_{19}H_{15}N_2O_2^+$: 303.1134; found 303.1138.

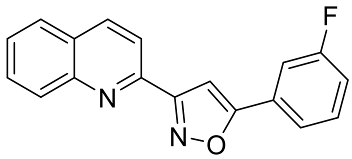

***5-(3-Fluorophenyl)-3-(quinolin-2-yl)isoxazole (3l)* [1,2].** Yield: 71%. yellow solid. Mp: 125-127℃.

^{1}H NMR (400 MHz, CDCl $_3$): δ 8.29 – 8.15 (m, 3H), 7.86 (dd, J = 8.2, 1.6 Hz, 1H), 7.76 (tt, J = 6.8, 1.5 Hz, 1H), 7.67 (dq, J = 7.8, 1.3 Hz, 1H), 7.63 – 7.55 (m, 2H), 7.50 – 7.44 (m, 1H), 7.41 (d, J = 1.4 Hz, 1H), 7.20 – 7.11 (m, 1H).

^{13}C NMR (101 MHz, CDCl $_3$): δ 169.2, 164.3, 162.9 (d, J = 246.0 Hz), 148.2 (d, J = 36.0 Hz), 137.0, 130.8, 130.7, 130.0, 129.7, 129.3 (d, J = 7.0 Hz), 128.4, 127.7, 127.4, 121.6 (d, J = 4.0 Hz), 119.0, 117.2 (d, J = 21.0 Hz), 112.8 (d, J = 23.0 Hz), 99.5.

^{19}F NMR (471 MHz, CDCl$_3$): δ -111.5.

HRMS (ESI) m/z: [M+H]$^+$calcd. for: $C_{18}H_{12}FN_2O^+$: 291.0934; found 291.0933.

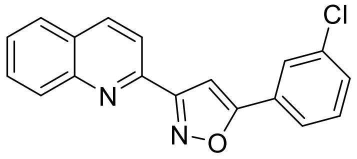

5-(3-Chlorophenyl)-3-(quinolin-2-yl)isoxazole (3m) [1]**.** Yield: 75%. yellow solid. Mp: 138-141℃.

^{1}H NMR (400 MHz, CDCl $_3$): δ 8.26 – 8.12 (m, 3H), 7.87 – 7.81 (m, 2H), 7.78 – 7.71 (m, 2H), 7.57 (ddd, J = 7.9, 7.0, 1.2 Hz, 1H), 7.45 – 7.34 (m, 3H).

^{13}C NMR (101 MHz, CDCl $_3$): δ 169.0, 164.2, 148.3, 148.0, 136.9, 135.1, 130.3, 130.2, 130.0, 129.7, 129.0, 128.4, 127.7, 127.4, 125.9, 125.9, 123.9, 119.0, 99.5, 99.5.

HRMS (ESI) m/z: [M+H]$^+$calcd. for: $C_{18}H_{12}ClN_2O^+$: 307.0638; found 307.0644.

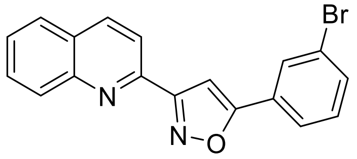

5-(3-Bromophenyl)-3-(quinolin-2-yl)isoxazole (3n) [1]**.** Yield: 85%. yellow solid. Mp: 147-149℃.

^{1}H NMR (400 MHz, CDCl $_3$): δ 8.23 (q, J = 8.5 Hz, 2H), 8.16 (d, J = 8.5 Hz, 1H), 8.03 (t, J = 1.8 Hz, 1H), 7.87 – 7.72 (m, 3H), 7.62 – 7.55 (m, 2H), 7.40 (s, 1H), 7.36 (t, J = 7.9 Hz, 1H).

^{13}C NMR (101 MHz, CDCl $_3$): δ 168.9, 164.2, 148.3, 148.0, 137.0, 133.1, 130.6, 130.0, 129.7, 129.2, 128.8, 128.4, 127.7, 127.4, 124.4, 123.1, 119.0, 99.5, 99.5.

HRMS (ESI) m/z: [M+H] $^+$calcd. for: $C_{18}H_{12}BrN_2O^+$: 351.0133; found 351.0139.

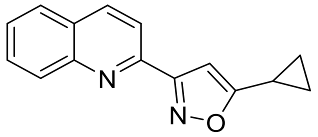

***5-Cyclopropyl-3-(quinolin-2-yl)isoxazole (3o)* [1,2].** Yield: 76%. yellow oil .

^{1}H NMR (400 MHz, CDCl $_3$): δ 8.21 (dd, *J* = 8.6, 0.8 Hz, 1H), 8.17 – 8.10 (m, 2H), 7.83 (dd, J = 8.1, 1.5 Hz, 1H), 7.73 (ddd, J = 8.5, 6.9, 1.5 Hz, 1H), 7.56 (ddd, J = 8.1, 6.9, 1.2 Hz, 1H), 6.72 (s, 1H), 2.19 – 2.08 (m, 1H), 1.13 – 1.02 (m, 4H).

^{13}C NMR (101 MHz, CDCl $_3$): δ 175.9, 163.6, 148.9, 148.0, 136.8, 129.8, 129.7, 128.3, 127.7, 127.2, 119.1, 97.6, 8.6, 8.2.

HRMS (ESI) m/z: [M+H] $^+$ calcd. for: $C_{15}H_{13}N_2O^+$: 237.1022; found 237.1027.

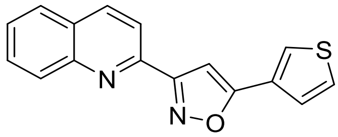

***3-(Quinolin-2-yl)-5-(thiophen-3-yl)isoxazole (3p)* [1].** Yield: 81%. yellow solid. Mp: 155-157℃.

^{1}H NMR (400 MHz, CDCl $_3$): δ 8.30 – 8.20 (m, 2H), 8.17 (d, J = 8.5 Hz, 1H), 7.90 – 7.82 (m, 2H), 7.79 – 7.83 (m, 1H), 7.59 (ddd, J = 8.3, 4.9, 1.6 Hz, 1H), 7.52 (dq, J = 4.9, 1.5 Hz, 1H), 7.45 (ddd, J = 4.8, 3.0, 1.5 Hz, 1H), 7.24 (t, J = 1.6 Hz, 1H).

^{13}C NMR (101 MHz, CDCl $_3$): δ 166.8, 164.0, 148.6, 148.0, 136.9, 130.0, 129.9, 129.7, 128.9, 128.4, 127.7, 127.3, 127.1, 125.5, 124.4, 119.2, 98.4.

HRMS (ESI) m/z: [M+H] $^+$ calcd. for: $C_{16}H_{11}N_2OS^+$: 279.0592; found 279.0599.

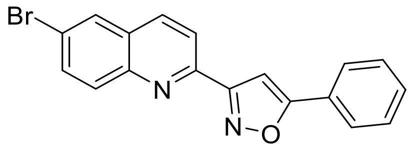

***3-(6-Bromoquinolin-2-yl)-5-phenylisoxazole (4a)* [1,2].** Yield: 92%. yellow solid. Mp: 177-179℃.

^{1}H NMR (400 MHz, CDCl$_3$): δ 8.22 (dd, *J* = 8.6, 2.5 Hz, 1H), 8.14 (dd, *J* = 8.6, 2.4 Hz, 1H), 8.03 – 7.99 (m, 2H), 7.90 – 7.86 (m, 2H), 7.80 (dt, *J* = 8.9, 2.4 Hz, 1H), 7.54 – 7.45 (m, 3H), 7.33 (d, *J* = 2.6 Hz, 1H).

^{13}C NMR (101 MHz, CDCl$_3$): δ 170.8, 163.8, 149.0, 146.6, 135.9, 133.4, 131.4, 130.3, 129.8, 129.4, 129.0, 127.3, 125.9, 121.3, 120.0, 98.5.

HRMS (ESI) m/z: [M+H]$^+$ calcd. for: $C_{18}H_{12}BrN_2O^+$: 351.0128; found 351.0131.

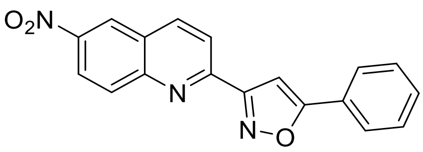

***3-(6-Nitroquinolin-2-yl)-5-phenylisoxazole (4b)* .** Yield: 41%. yellow solid. Mp: 233-237℃.

^{1}H NMR (400 MHz, DMSO): δ 9.04 (d, J = 2.6 Hz, 1H), 8.80 (d, J = 8.6 Hz, 1H), 8.45 (dd, J = 9.3, 2.6 Hz, 1H), 8.24 (dd, J = 16.1, 8.9 Hz, 2H), 7.95 (dd, J = 7.4, 2.1 Hz, 2H), 7.63 (s, 1H), 7.52 (q, J = 4.9 Hz, 3H).

^{13}C NMR (400 MHz, DMSO): δ 171.0, 163.7, 151.6, 149.7, 145.8, 140.4, 131.4, 131.2, 129.7, 127.5, 126.9, 126.2, 125.5, 124.0, 120.9, 99.5.

HRMS (ESI) m/z: [M+H]$^+$ calcd. for: $C_{18}H_{12}N_3O_3^+$: 318.0879; found 317.0885.

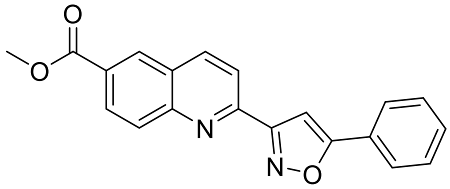

***Methyl 2-(5-phenylisoxazol-3-yl)quinoline-6-carboxylate (4c)* [2].** Yield: 90%. yellow solid. Mp: 172-174℃.

^{1}H NMR (400 MHz, CDCl $_3$): δ 8.60 (d, J = 1.9 Hz, 1H), 8.36 – 8.27 (m, 3H), 8.21 – 8.17 (m, 1H), 7.91 – 7.88 (m, 2H), 7.52 – 7.46 (m, 3H), 7.38 (s, 1H), 4.00 (s, 3H).

^{13}C NMR (101 MHz, CDCl $_3$): δ 170.9, 166.5, 163.8, 150.7, 149.8, 138.2, 130.7, 130.4, 130.0, 129.4, 129.1, 128.6, 127.5, 127.3, 125.9, 119.8, 98.6, 98.6, 52.5.

HRMS (ESI) m/z: [M+H]$^+$calcd. for: $C_{20}H_{15}N_2O_3^+$: 331.1077; found 331.1080.

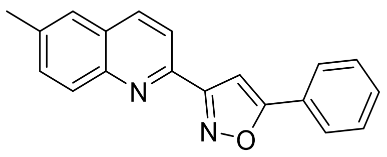

***3-(6-Methylquinolin-2-yl)-5-phenylisoxazole (4d)* [1,2].** Yield: 83%. yellow solid. Mp: 129-131℃.

^{1}H NMR (400 MHz, CDCl$_3$): δ 8.21 – 8.14 (m, 2H), 8.06 (d, *J* = 8.5 Hz, 1H), 7.91 – 7.87 (m, 2H), 7.62 – 7.56 (m, 2H), 7.52 – 7.43 (m, 3H), 7.37 (s, 1H), 2.55 (s, 3H).

^{13}C NMR (101 MHz, CDCl$_3$): δ 170.5, 164.2, 147.8, 146.6, 137.4, 136.2, 132.2, 130.2, 129.4, 129.0, 128.5, 127.5, 126.6, 125.9, 119.1, 98.6, 21.7.

HRMS (ESI) m/z: [M+H]$^+$ calcd. for: $C_{19}H_{15}N_2O^+$: 287.1179; found 287.1185.

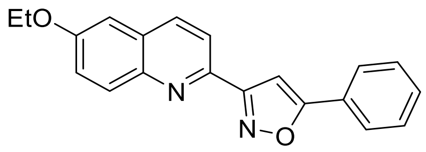

***3-(6-Ethoxyquinolin-2-yl)-5-phenylisoxazole (4e)* [2].** Yield: 62%. yellow solid. Mp: 205-207℃.

^{1}H NMR (400 MHz, CDCl$_3$): δ 8.19 – 8.11 (m, 2H), 8.06 (dd, J = 9.2, 1.4 Hz, 1H), 7.90 (dt, J = 7.9, 1.7 Hz, 2H), 7.52 – 7.45 (m, 3H), 7.40 (dt, J = 9.2, 2.2 Hz, 1H), 7.37 – 7.35 (m, 1H), 7.27 – 7.24 (m, 1H), 4.22 – 4.13 (m, 2H), 1.53 – 1.48 (m, 3H).

^{13}C NMR (101 MHz, CDCl$_3$): δ 170.5, 164.2, 157.8, 146.2, 144.0, 135.5, 131.1, 130.2, 129.6, 129.0, 127.5, 125.9, 123.0, 119.4, 105.9, 98.5, 63.9, 14.7.

HRMS (ESI) m/z: [M+H]$^+$ calcd. for: $C_{19}H_{15}N_2O_2^+$: 303.1134; found 303.1138.

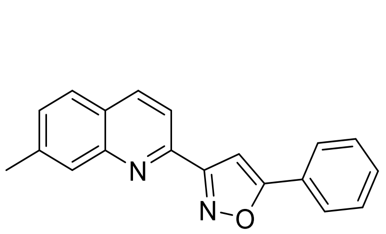

***3-(7-Methylquinolin-2-yl)-5-phenylisoxazole (4f)* .** Yield: 80%. yellow solid. Mp: 131-133℃.

^{1}H NMR (400 MHz, CDCl$_3$): δ 8.14 – 8.06 (m, 2H), 8.01 – 7.89 (m, 2H), 7.82 – 7.78 (m, 2H), 7.66 (d, J = 8.8 Hz, 1H), 7.19 (dt, J = 8.9, 2.6 Hz, 1H), 7.14 (q, J = 2.7 Hz, 1H), 4.47 (dd, J = 18.1, 2.4 Hz, 1H), 4.16 (dd, J = 18.1, 2.3 Hz, 1H), 3.97 – 3.88 (s, 3H).

^{13}C NMR (101 MHz, CDCl$_3$): δ 158.6, 158.5, 148.6, 146.3, 135.5, 134.7, 133.5, 131.3, 130.5, 130.0, 129.8, 129.3, 128.2, 127.4, 126.2, 124.3, 121.8, 119.9, 119.7, 105.5, 55.4.

HRMS (ESI) m/z: [M+Na]$^+$ calcd. for: $C_{19}H_{14}N_2NaO^+$: 309.1004; found309.0991.

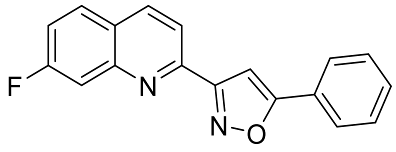

***3-(7-Fluoroquinolin-2-yl)-5-phenylisoxazole (4g)* [1].** Yield: 80%. yellow solid. Mp: 131-133℃.

^{1}H NMR (400 MHz, CDCl$_3$): δ 8.26 – 8.17 (m, 2H), 7.91 – 7.77 (m, 4H), 7.49 (qd, J = 7.8, 6.8, 3.7 Hz, 3H), 7.40 – 7.34 (m, 2H).

^{13}C NMR (101 MHz, CDCl$_3$): δ 170.7, 163.9, 163.2 (d, J = 250.0 Hz), 149.6, 149.0, 148.9, 136.8, 130.3, 129.7 (d, J = 10.0 Hz), 129.0, 127.4, 125.9, 125.4, 118.5, 118.4, 117.8 (d, J = 25.0 Hz), 113.3 (d, J = 10.0 Hz), 98.6.

^{19}F NMR (471 MHz, CDCl3): δ -108.8.

HRMS (ESI) m/z: [M+H]$^+$calcd. for: $C_{18}H_{12}FN_2O^+$: 291.0934; found 291.0935.

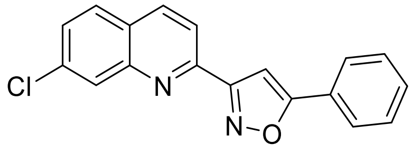

***3-(7-Chloroquinolin-2-yl)-5-phenylisoxazole (4h)* [1,2].** Yield: 75%. white solid. Mp: 146-148℃.

^{1}H NMR (400 MHz, CDCl$_3$): δ 8.22 (s, 2H), 8.16 (d, J = 2.1 Hz, 1H), 7.90 – 7.87 (m, 2H), 7.78 (dd, J = 8.7, 0.5 Hz, 1H), 7.54 – 7.47 (m, 4H), 7.34 (s, 1H).

^{13}C NMR (101 MHz, CDCl$_3$): δ 170.8, 163.8, 149.6, 148.3, 136.7, 135.8, 130.3, 129.0, 128.9, 128.7, 128.3, 127.3, 126.7, 125.9, 119.3, 98.6, 98.5.

HRMS (ESI) m/z: [M+H]$^+$calcd. for: $C_{18}H_{12}ClN_2O^+$: 307.0633; found 307.0638.

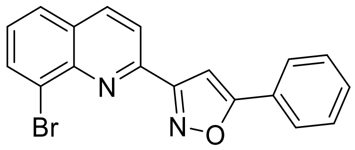

***3-(8-Bromoquinolin-2-yl)-5-phenylisoxazole (4i)* [1].** Yield: 54%. white solid. Mp: 142-144℃.

^{1}H NMR (400 MHz, CDCl$_3$): δ 8.27 (q, J = 8.5 Hz, 2H), 8.08 (dd, J = 7.5, 1.3 Hz, 1H), 7.93 – 7.90 (m, 2H), 7.81 (dd, J = 8.1, 1.3 Hz, 1H), 7.53 – 7.40 (m, 5H).

^{13}C NMR (101 MHz, CDCl$_3$): δ 170.7, 164.0, 149.4, 144.9, 137.4, 133.6, 130.3, 129.6, 129.0, 127.7, 127.6, 127.4, 126.0, 125.5, 119.8, 99.0, 98.9.

HRMS (ESI) m/z: [M+H]$^+$calcd. for: $C_{18}H_{12}BrN_2O^+$: 351.0133; found 351.0139.

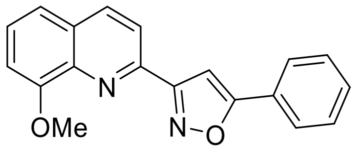

***3-(8-Methoxyquinolin-2-yl)-5-phenylisoxazole(4j)* [2].** Yield: 72%. white solid. Mp: 129-131℃.

^{1}H NMR (400 MHz, CDCl$_3$): δ 8.29 – 8.22 (m, 2H), 7.92 – 7.88 (m, 2H), 7.54 – 7.43 (m, 6H), 7.10 (dd, J = 7.7, 1.3 Hz, 1H), 4.13 (s, 3H).

^{13}C NMR (101 MHz, CDCl$_3$): δ 170.5, 164.2, 155.5, 147.6, 139.9, 137.0, 130.2, 129.6, 129.0, 127.6, 127.5, 125.9, 119.7, 119.6, 108.4, 99.0, 99.0, 56.2.

HRMS (ESI) m/z: [M+H]$^+$calcd. for: $C_{19}H_{15}N_2O_2^+$: 303.1128; found 303.1135.

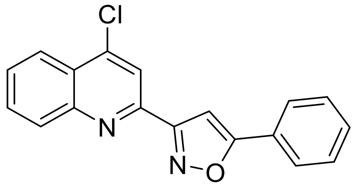

***3-(4-Chloroquinolin-2-yl)-5-phenylisoxazole (4k)* [2].** Yield: 34%. yellow solid. Mp: 149-151℃.

^{1}H NMR (400 MHz, CDCl$_3$): δ 8.31 (s, 1H), 8.24 (dd, J = 8.4, 1.4 Hz, 1H), 8.17 (dd, J = 8.4, 1.1 Hz, 1H), 7.87 (dd, J = 7.7, 1.8 Hz, 2H), 7.82 – 7.76 (m, 1H), 7.70 – 7.64 (m, 1H), 7.50 – 7.46 (m, 3H), 7.34 (s, 1H).

^{13}C NMR (101 MHz, CDCl $_3$): δ 170.9, 163.3, 148.7, 148.5, 143.3, 130.8, 130.5, 130.4, 130.1, 129.0, 128.2, 127.3, 126.5, 125.9, 124.2, 119.2, 98.5, 98.5.

HRMS (ESI) m/z: [M+H] $^+$calcd. for: $C_{19}H_{15}N_2O_2^+$: 307.0633; found 307.0637.

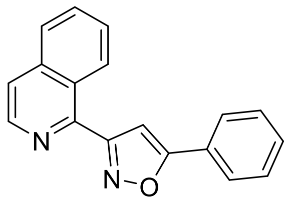

***3-(Isoquinolin-1-yl)-5-phenylisoxazole (4l)* [1,2].** Yield: 93%. yellow solid. Mp: 96-98℃.

^{1}H NMR (400 MHz, $CDCl_3$) δ 9.19 (d, J = 8.6 Hz, 1H), 8.65 (d, J = 5.5 Hz, 1H), 7.94 – 7.87 (m, 3H), 7.77 – 7.69 (m, 3H), 7.53 – 7.45 (m, 3H), 7.29 (s, 1H).

^{13}C NMR (101 MHz, $CDCl_3$) δ 169.6, 164.4, 148.5, 142.0, 136.9, 130.5, 130.2, 129.0, 128.4, 127.7, 127.3, 127.0, 126.8, 125.9, 122.2, 101.0.

HRMS (ESI) m/z: [M+H] $^+$calcd. for: C18H13N2O$^+$: 273.1022, found 273.1028.

Single crystal X-ray diffraction

Crystals of compound **3i** were obtained by slow diffusion from a solution of the compound in $CHCl_3$ layered with petroleum ether at room temperature for several days (Figure S1). Crystal data and details of the structure determination are presented in Table S1.

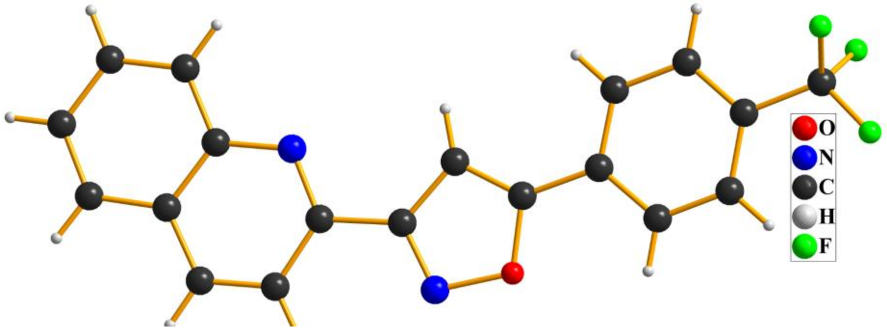

Figure S1. Crystal structure of **3i**

Table S1: The single crystal data of compounds **3i**

Phase	**3**i
Identification code	20230826a_a
Empirical formula	$C_{19}H_{11}F_3N_2O$
Formula weight	340.30
Temperature/K	296
Crystal system	triclinic
Space group	P-1
a/Å	7.2105(9)
b/Å	9.3278(11)
c/Å	12.3469(14)
α/°	90.218(4)
β/°	104.858(4)
γ/°	102.493(4)
Volume/Å^3	782.11(16)
Z	2
ρ_{calc}g/cm^3	1.445
μ/mm^{-1}	0.115
F(000)	348.0
Crystal size/mm^3	0.2 × 0.18 × 0.08
Radiation	MoKα (λ = 0.71073)
2Θ range for data collection/°	3.42 to 55.174
Index ranges	-9 ≤ h ≤ 9, -12 ≤ k ≤ 9, -16 ≤ l ≤ 16
Reflections collected	11926
Independent reflections	3612 [R_{int} = 0.0347, R_{sigma} = 0.0439]
Data/restraints/parameters	3612/0/226
Goodness-of-fit on F^2	1.033
Final R indexes [I>=2σ (I)]	R_1 = 0.0690, wR_2 = 0.1686
Final R indexes [all data]	R_1 = 0.1190, wR_2 = 0.1972
Largest diff. peak/hole / e Å^{-3}	0.40/-0.29

References

1. Meng, L.; Zhang S.W.; Jia X.D.; Lv I.Z.;Yuan Y. *Tetrahedron Letters,* **2019,** 60, 1443-1447.
2. D, H, Wang.; F, Zhang.; F, H, Xiao.; G,J, Deng.; Org. Biomol. Chem, **2019**, 17, 9163-9168.
3. J, M. Pérez.; D, J, Ramón. ACS Sustainable Chem. Eng. **2015**, 9, 2343–2349.

^{1}H NMR spectra of compound **3a**

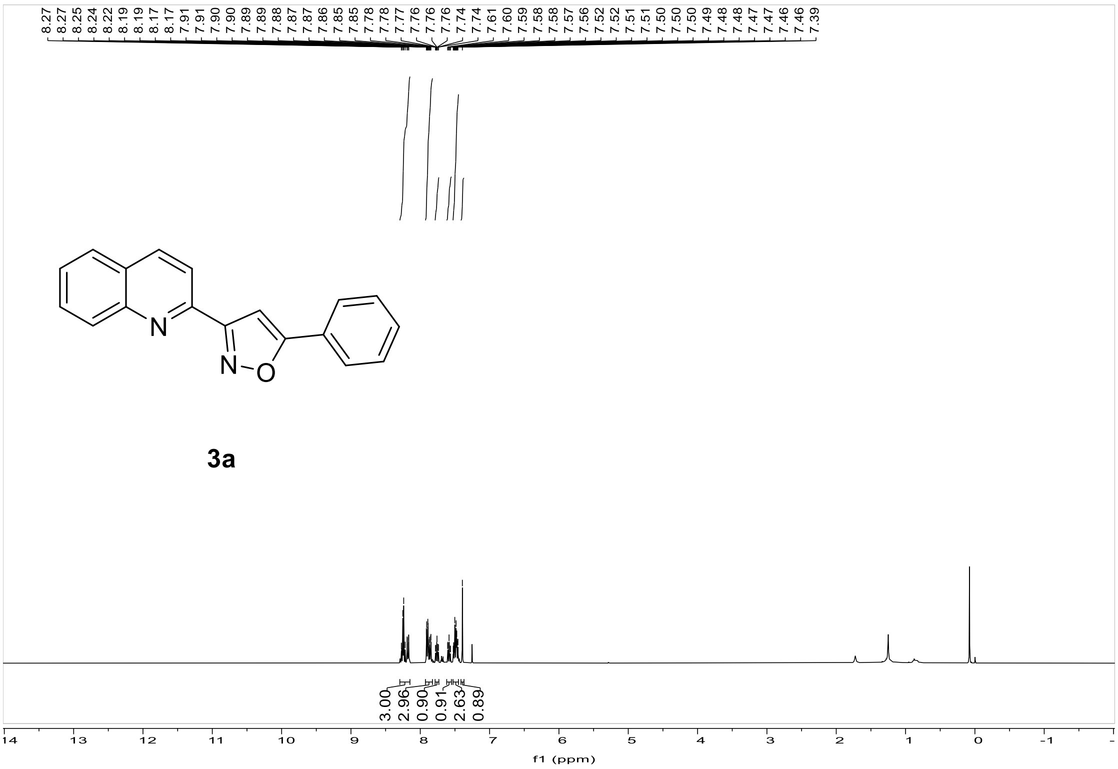

^{13}C{^{1}H} NMR spectra of compound **3a**

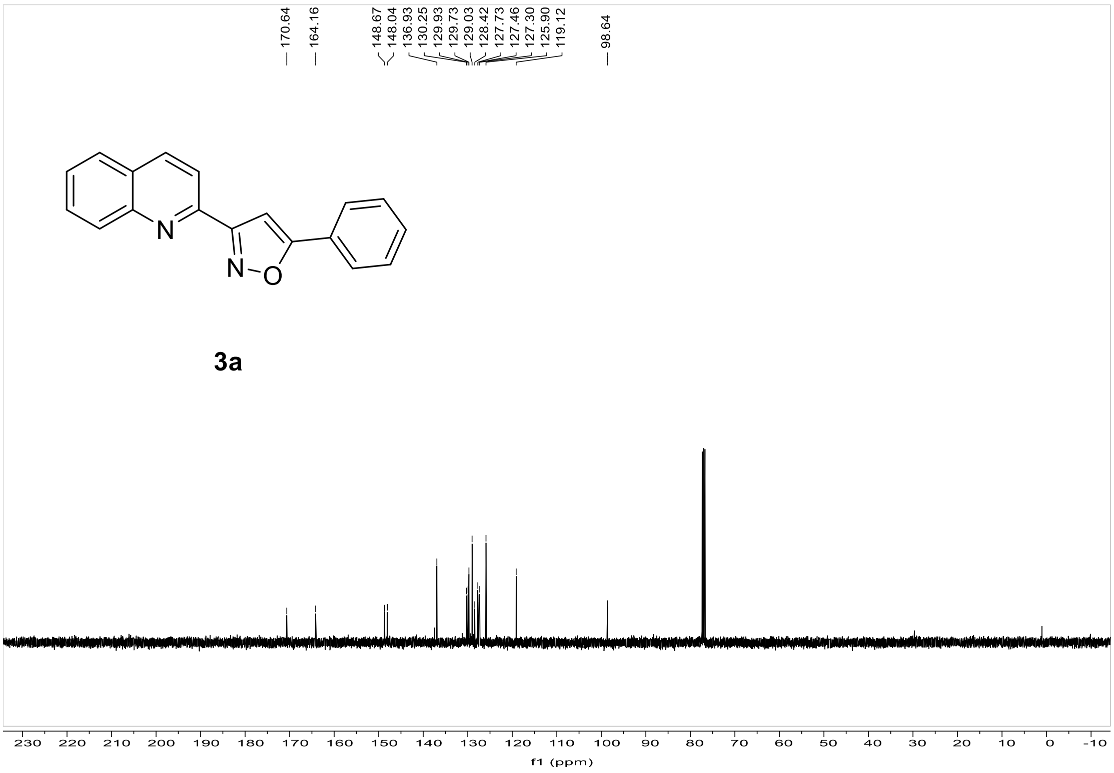

^{1}H NMR spectra of compound **3b**

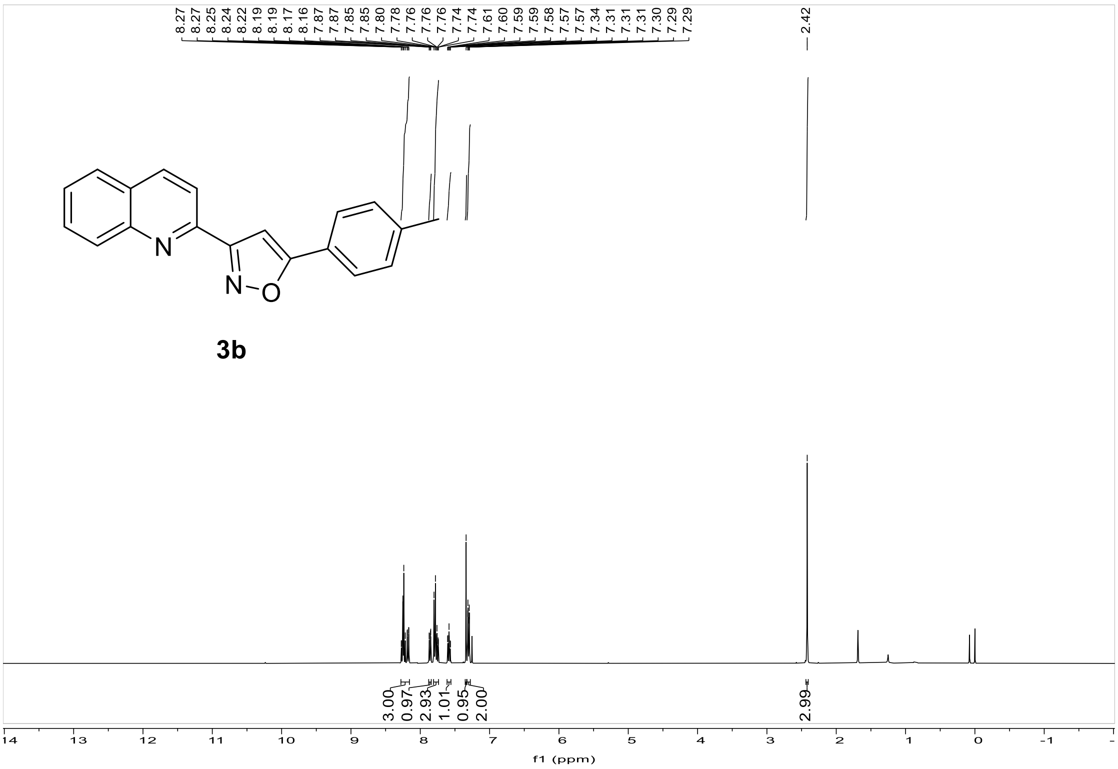

$^{13}C\{^{1}H\}$ NMR spectra of compound **3b**

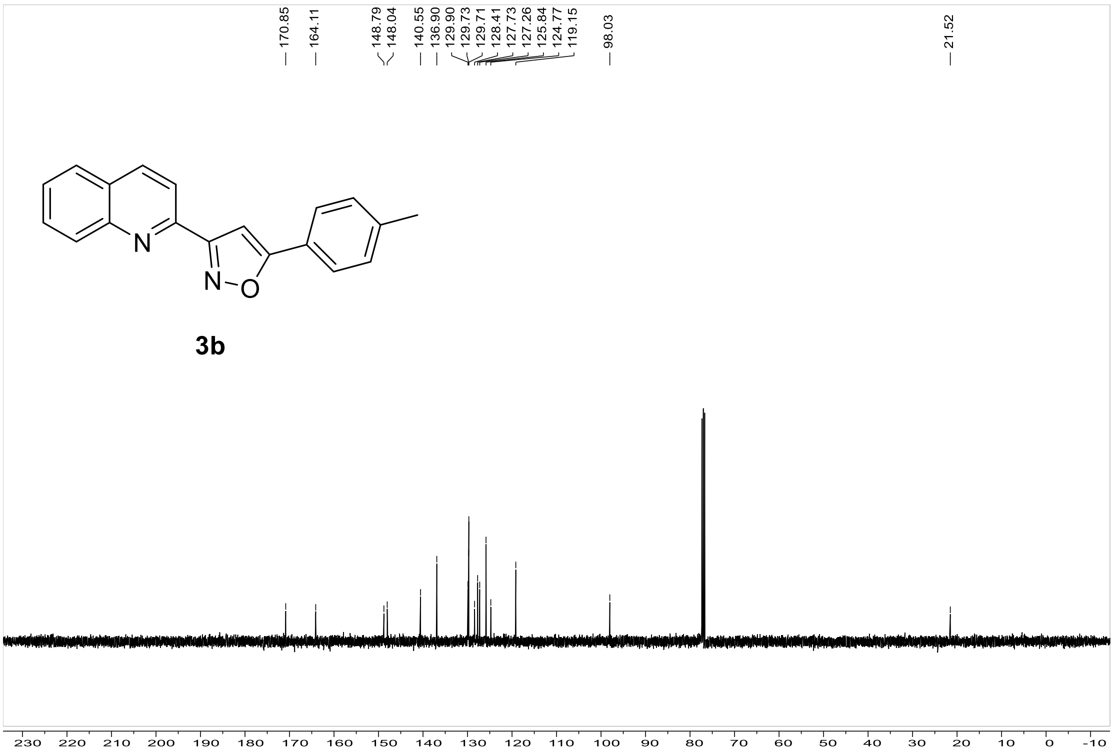

^{1}H NMR spectra of compound **3c**

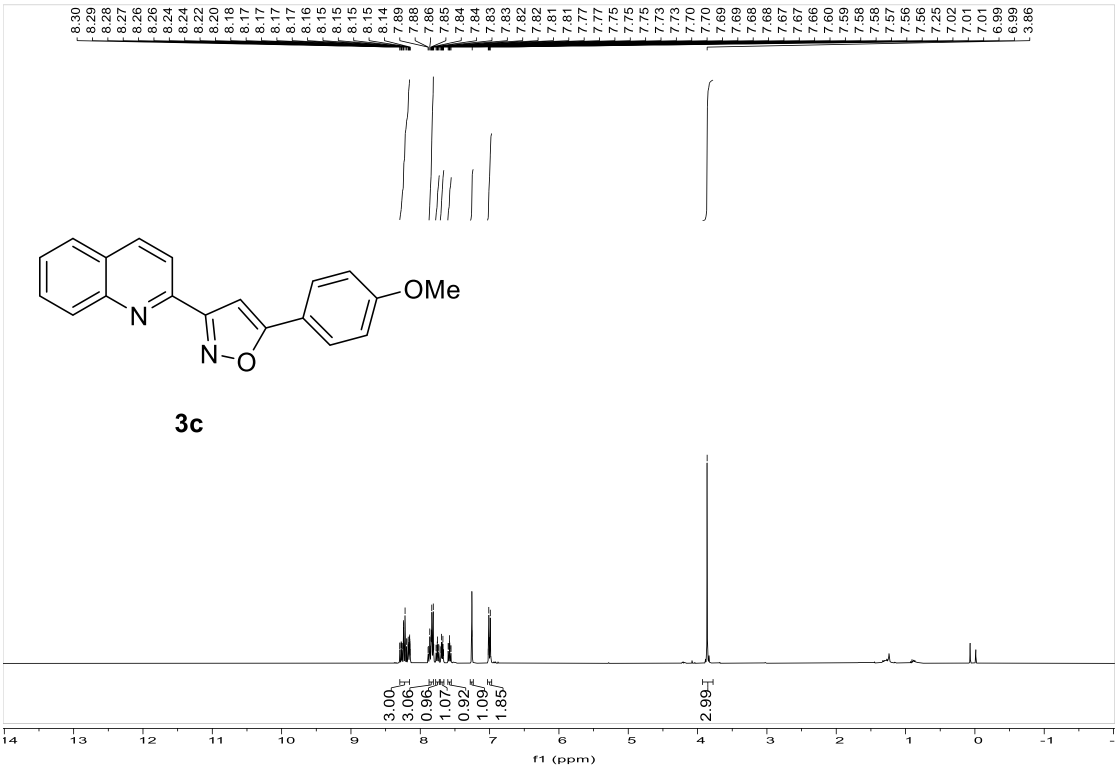

$^{13}C\{^{1}H\}$ NMR spectra of compound **3c**

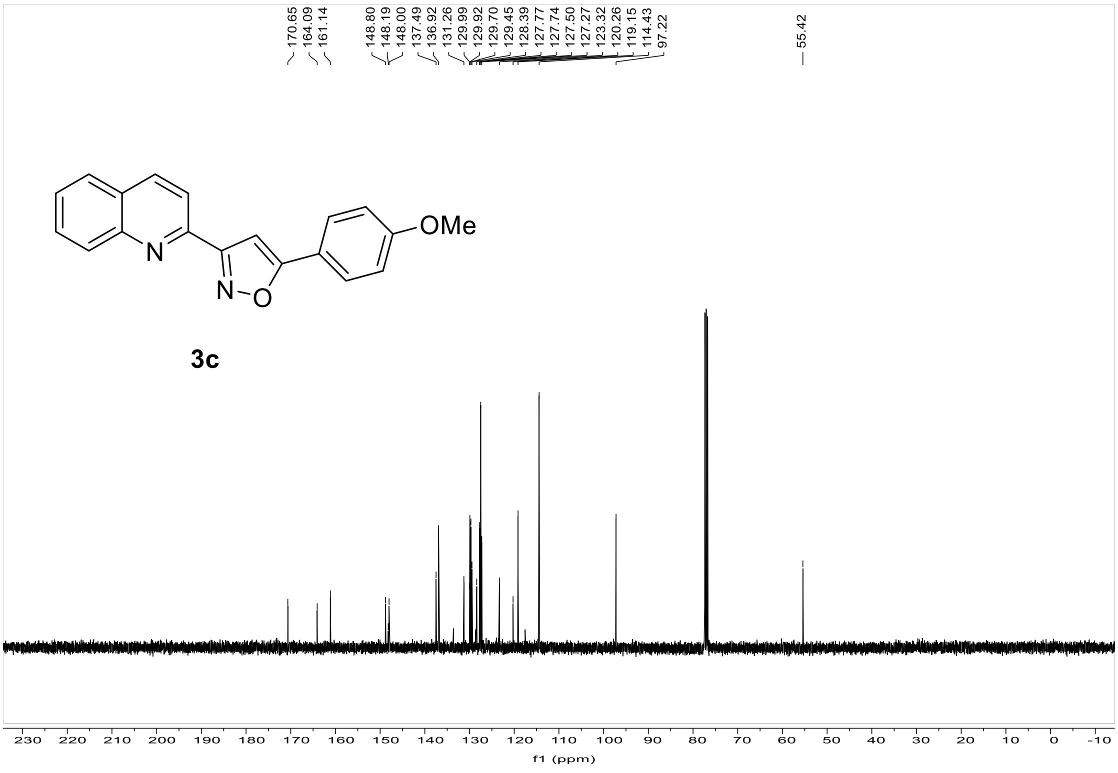

^{1}H NMR spectra of compound **3d**

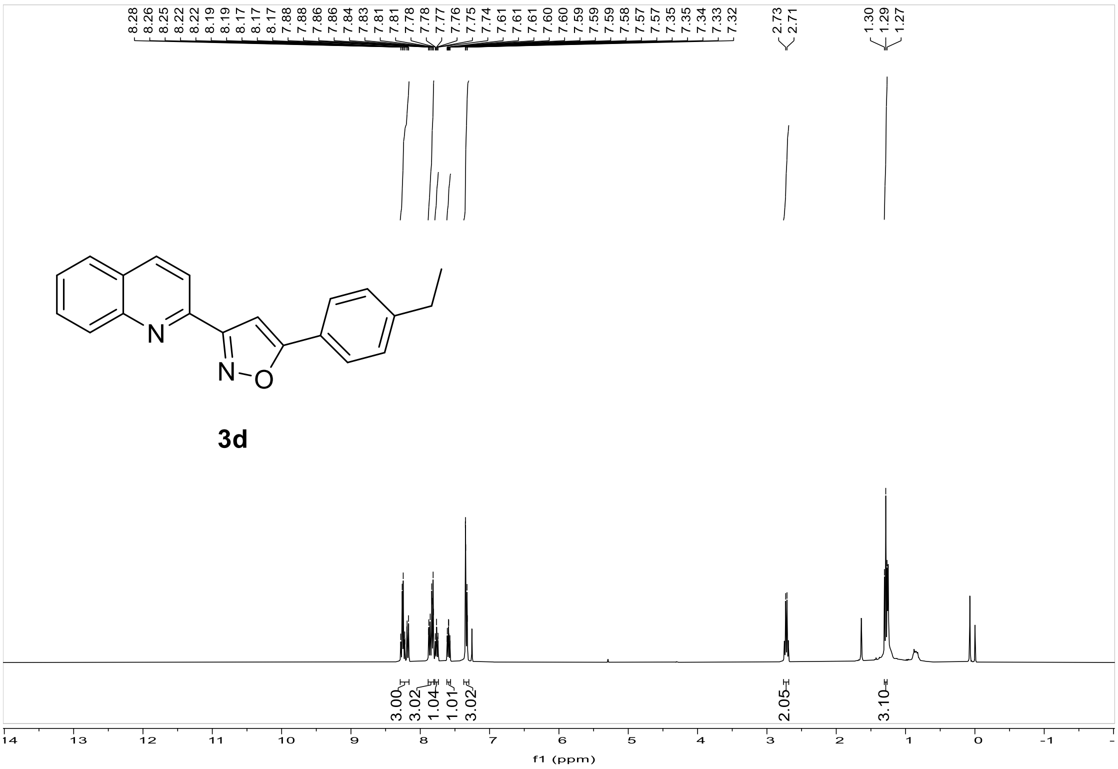

^{13}C{^{1}H} NMR spectra of compound **3d**

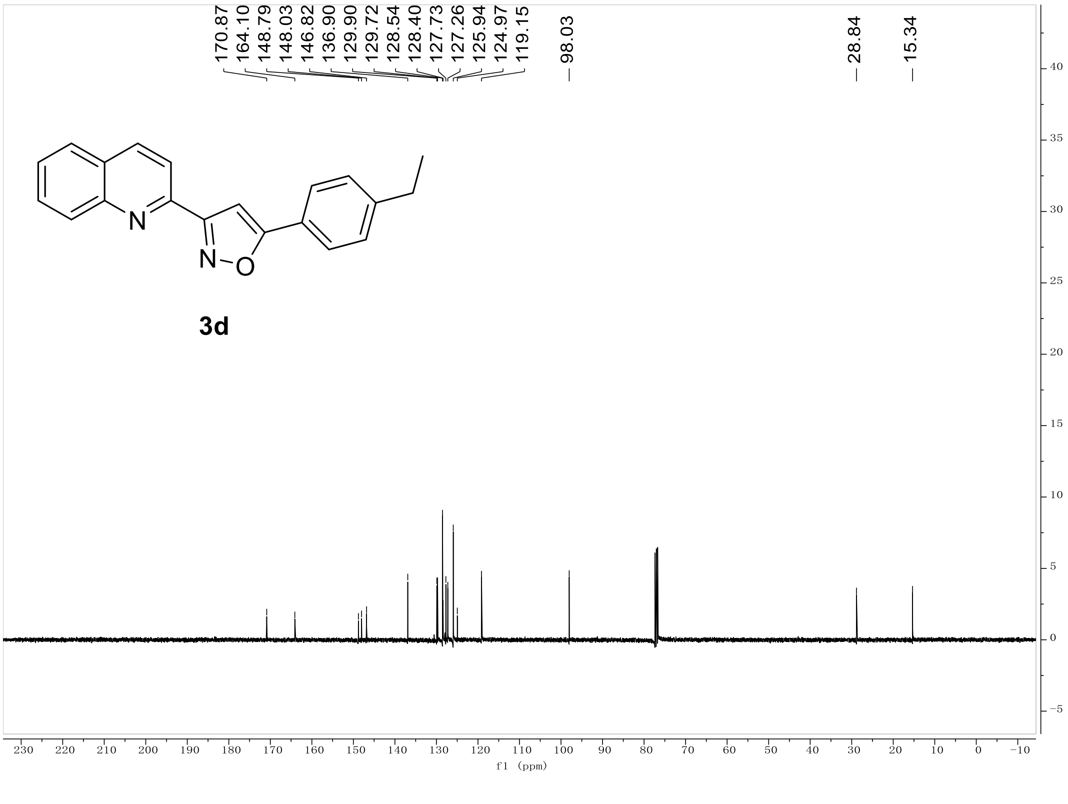

^{1}H NMR spectra of compound **3e**

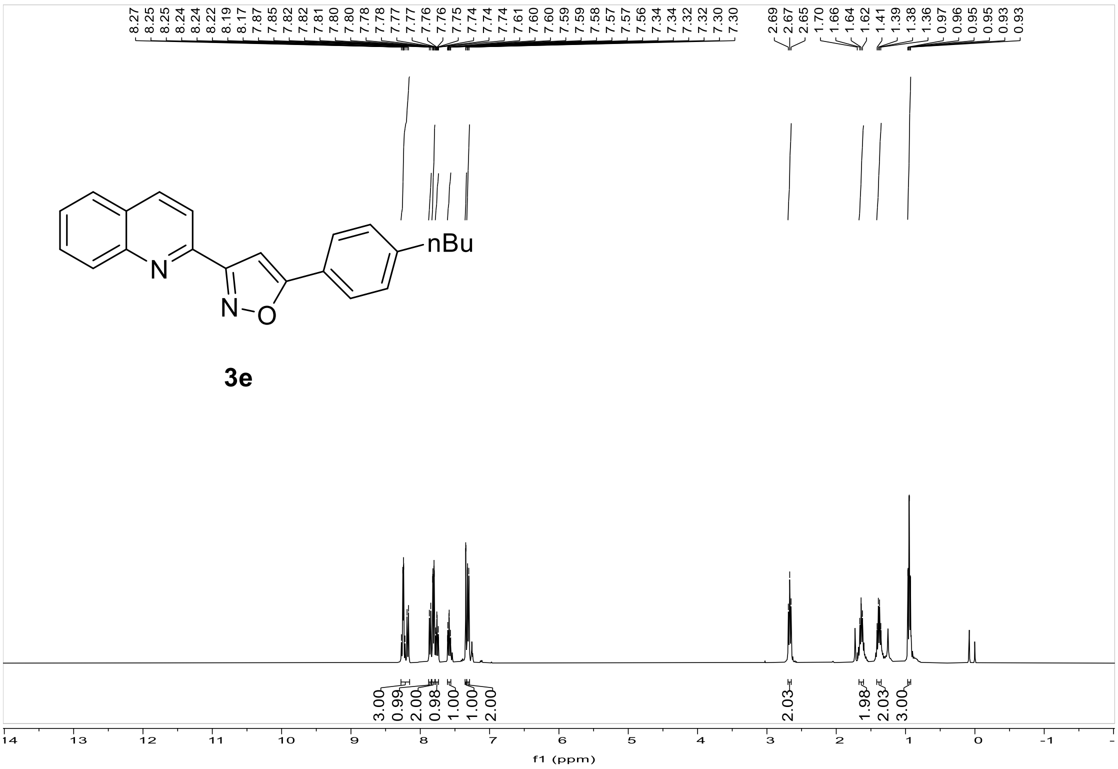

^{13}C{^{1}H} NMR spectra of compound **3e**

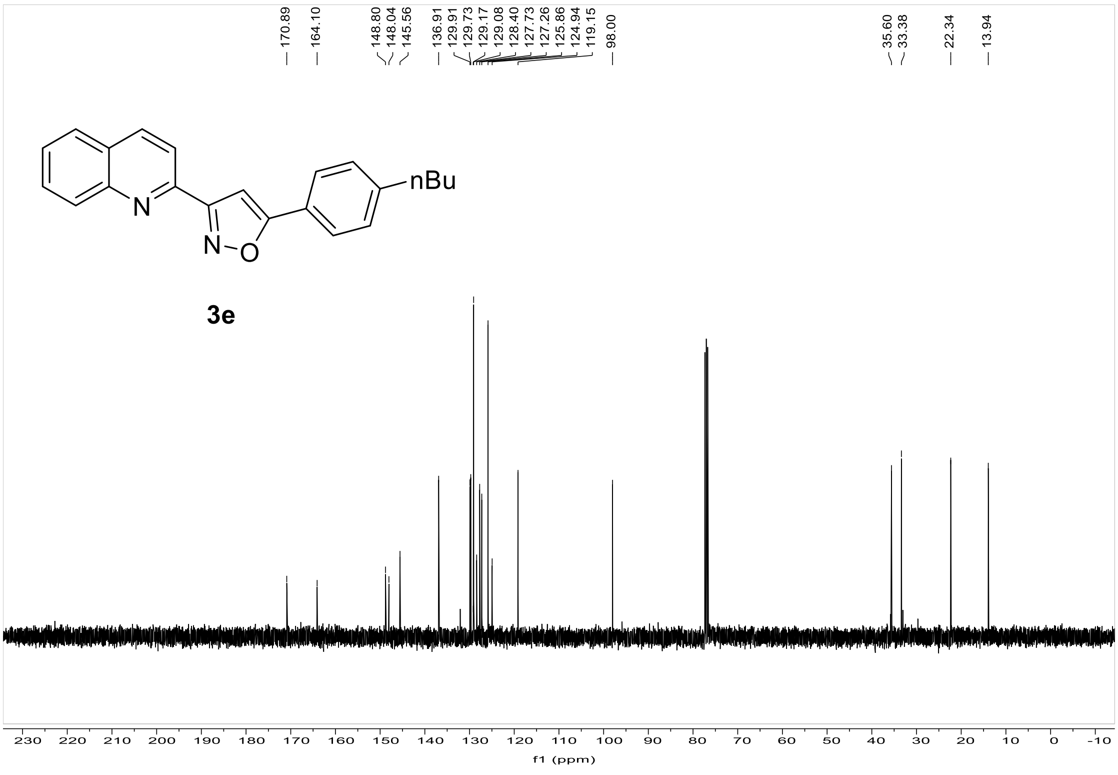

^{1}H NMR spectra of compound **3f**

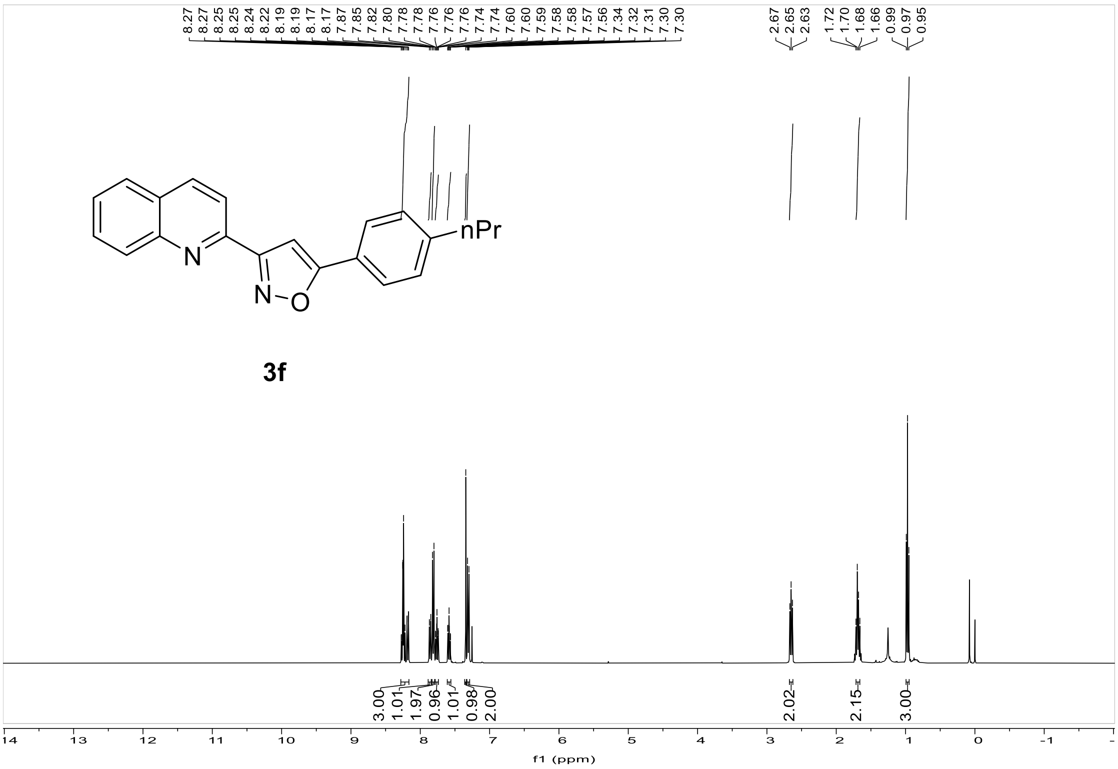

^{13}C{^{1}H} NMR spectra of compound **3f**

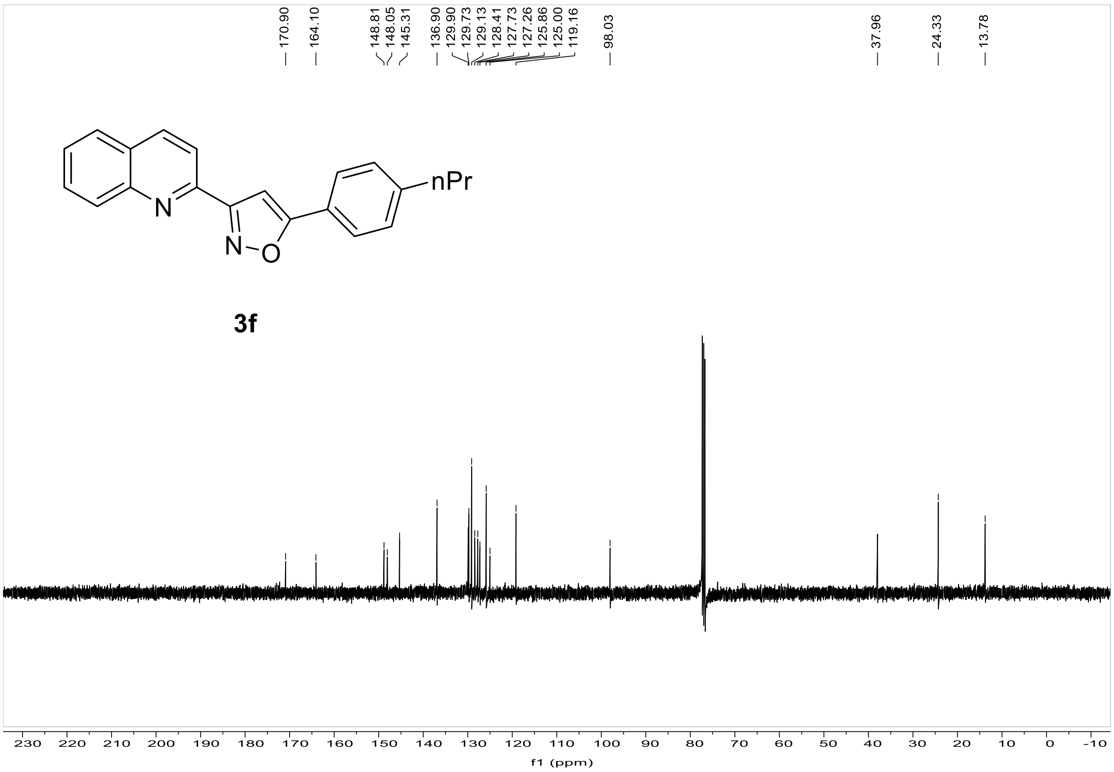

^{1}H NMR spectra of compound **3g**

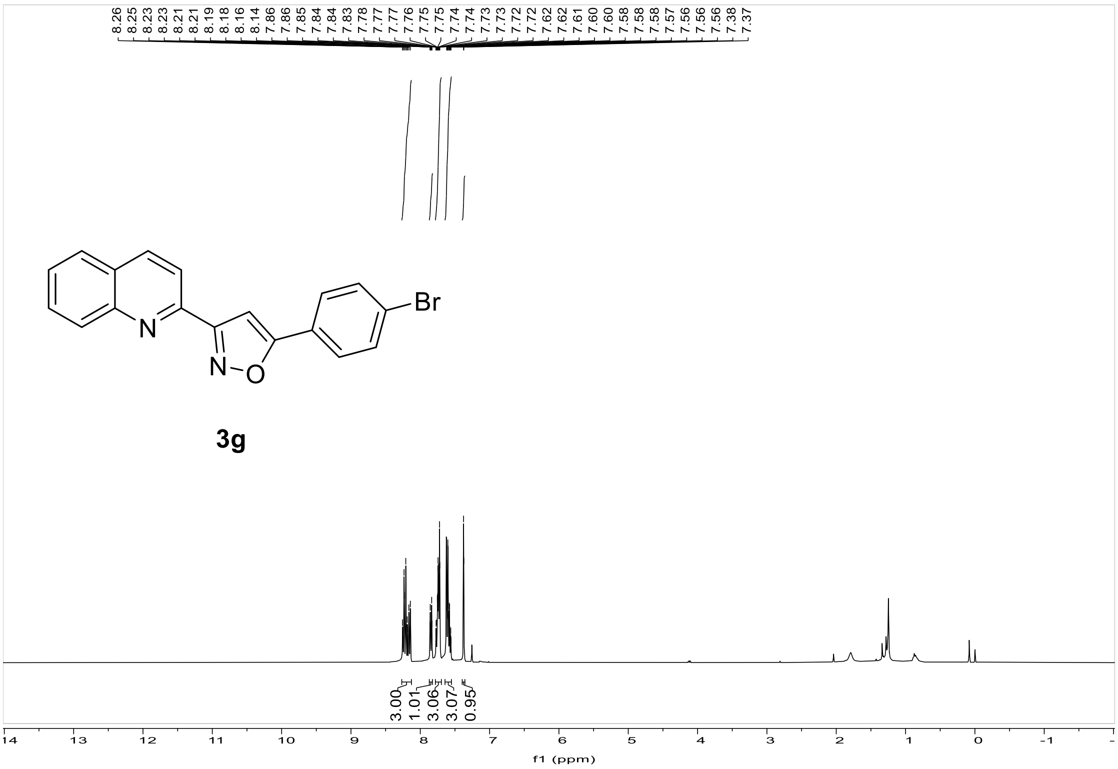

^{13}C{^{1}H} NMR spectra of compound **3g**

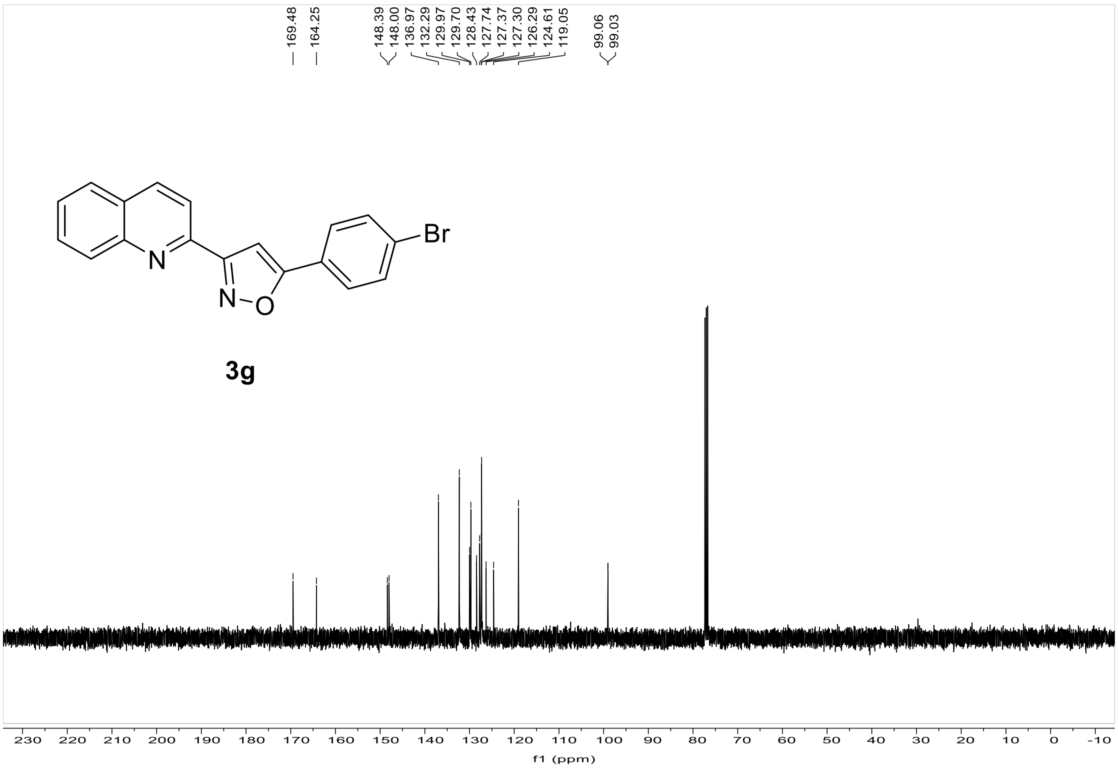

^{1}H NMR spectra of compound **3h**

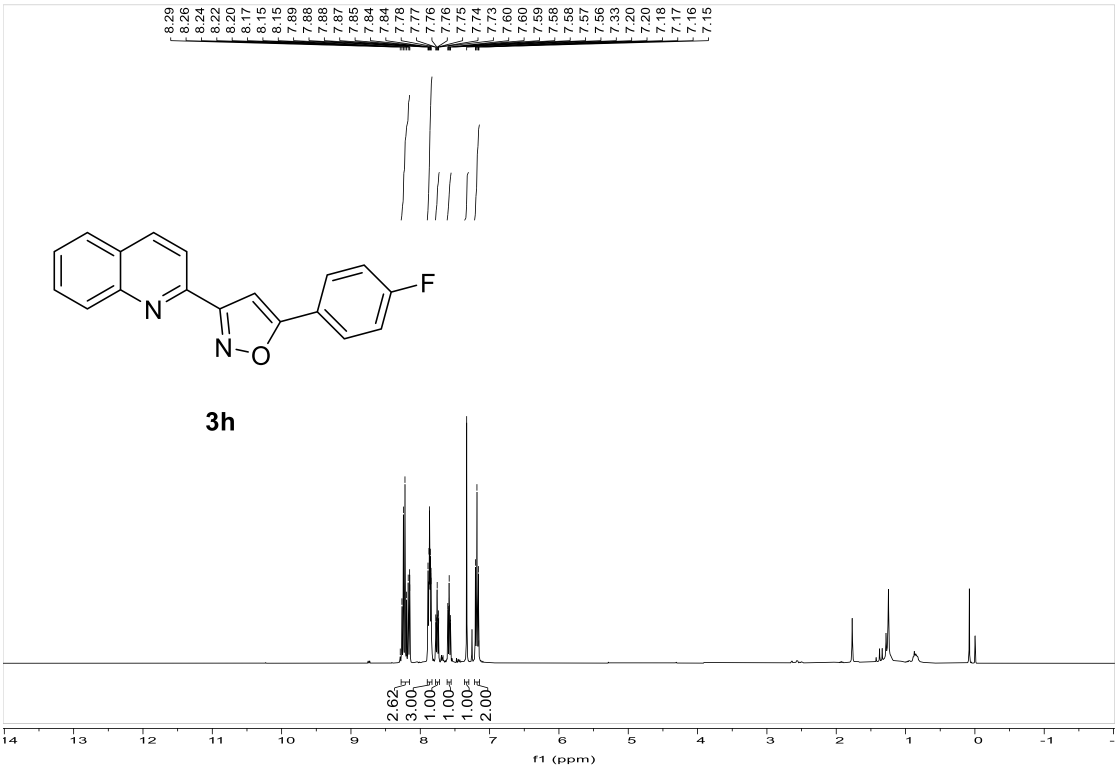

^{13}C{^{1}H} NMR spectra of compound **3h**

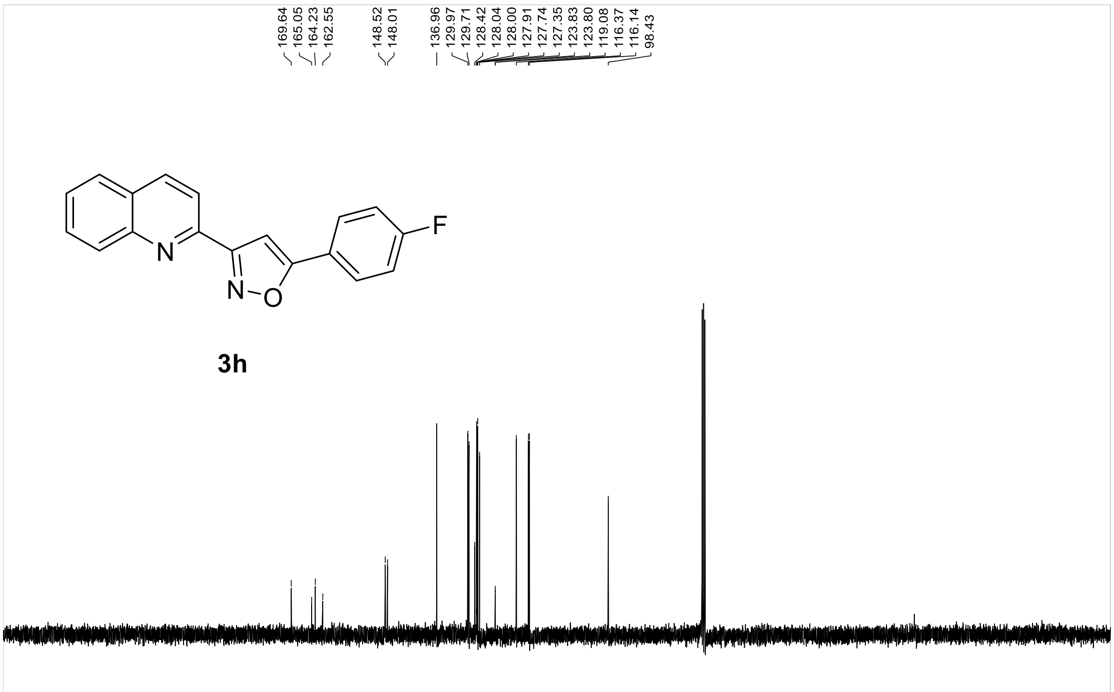

^{19}F NMR spectra of compound **3h**

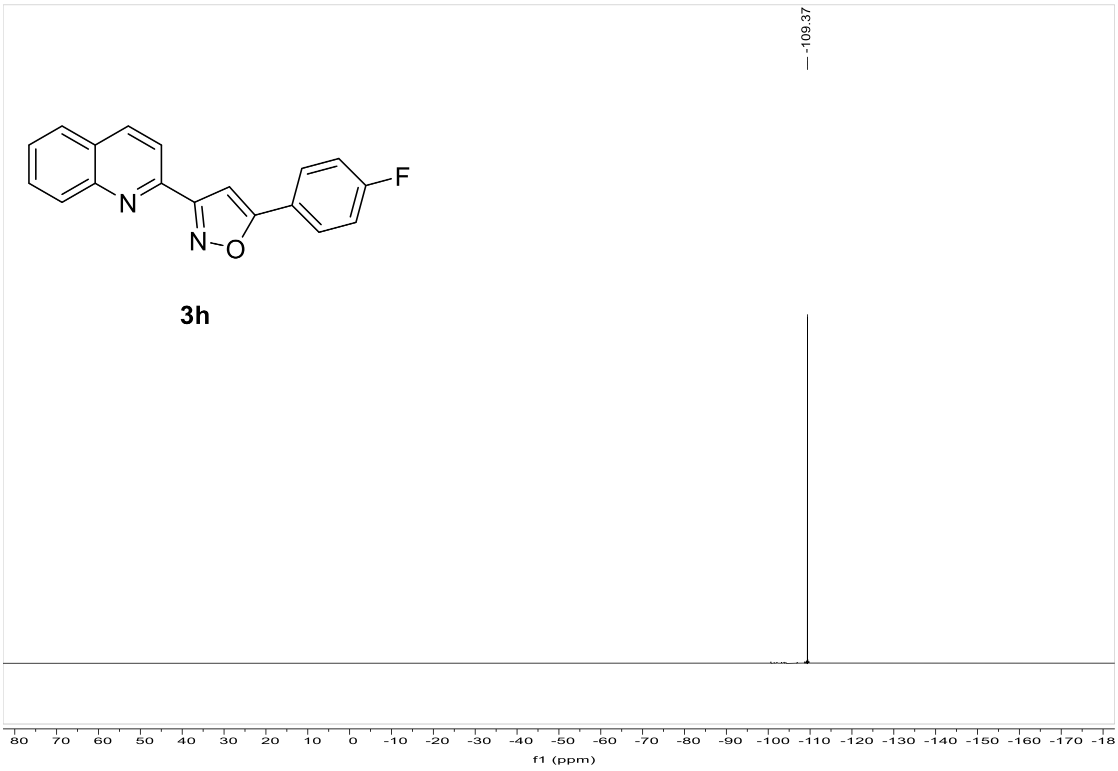

^{1}H NMR spectra of compound **3i**

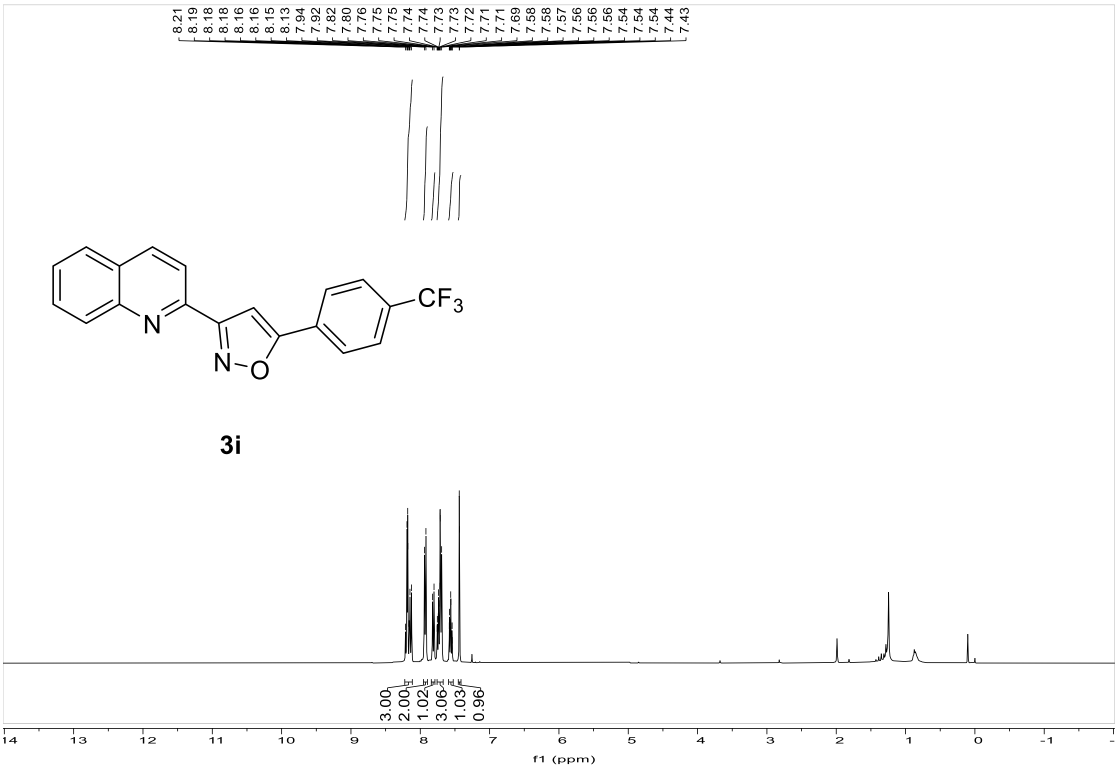

^{13}C{^{1}H} NMR spectra of compound **3i**

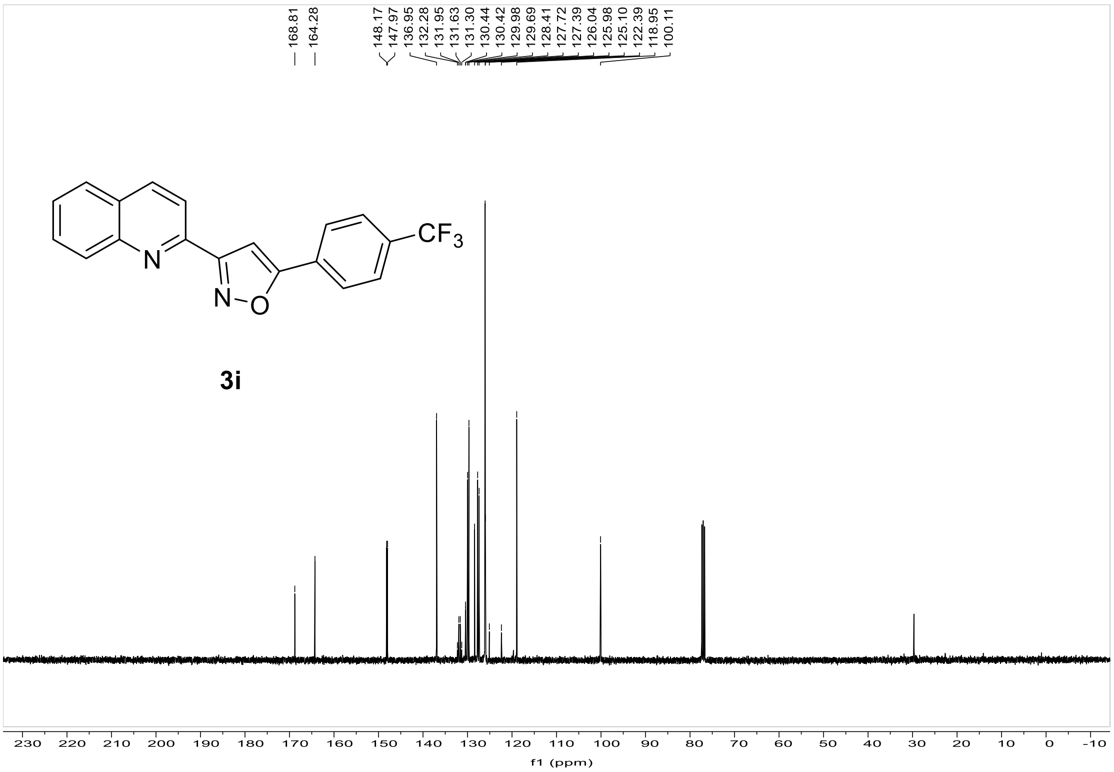

^{19}F NMR spectra of compound **3i**

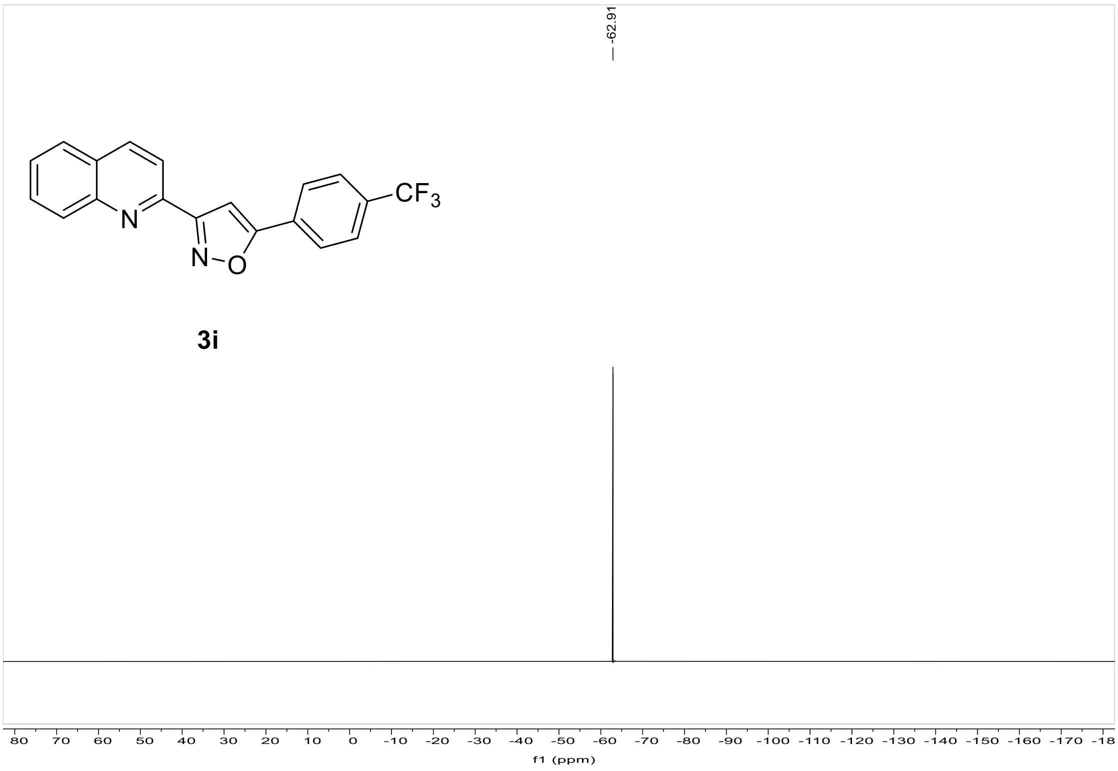

^{1}H NMR spectra of compound **3j**

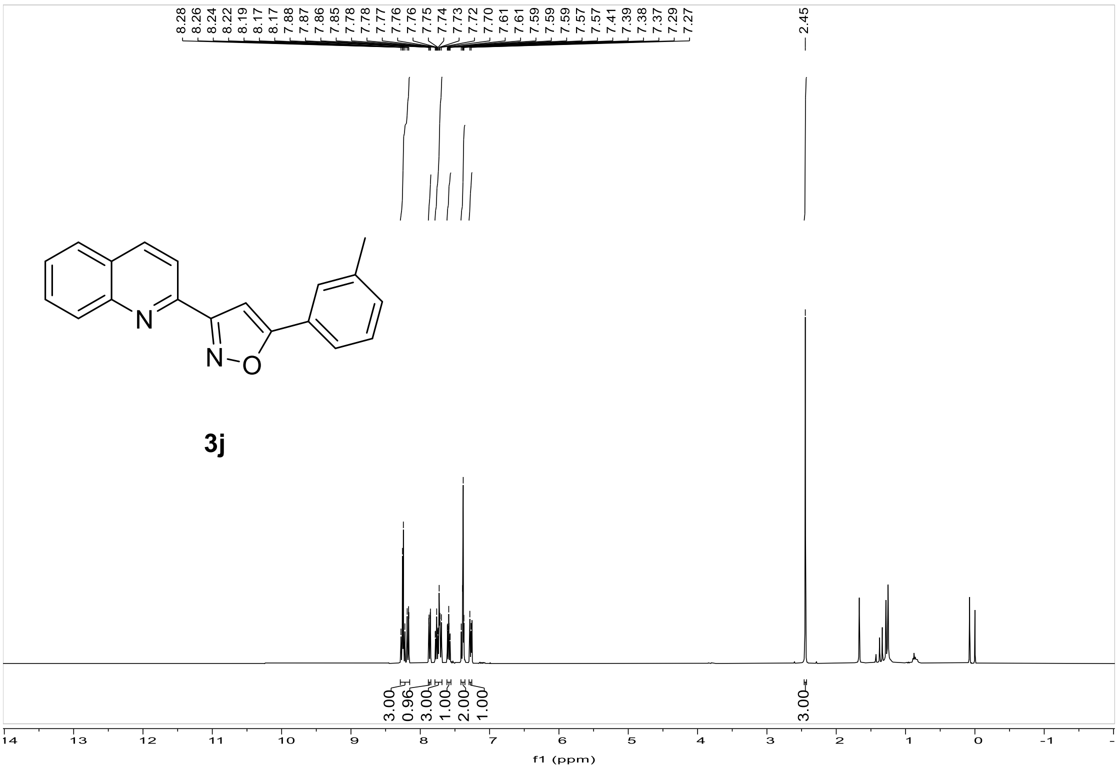

^{13}C{^{1}H} NMR spectra of compound **3j**

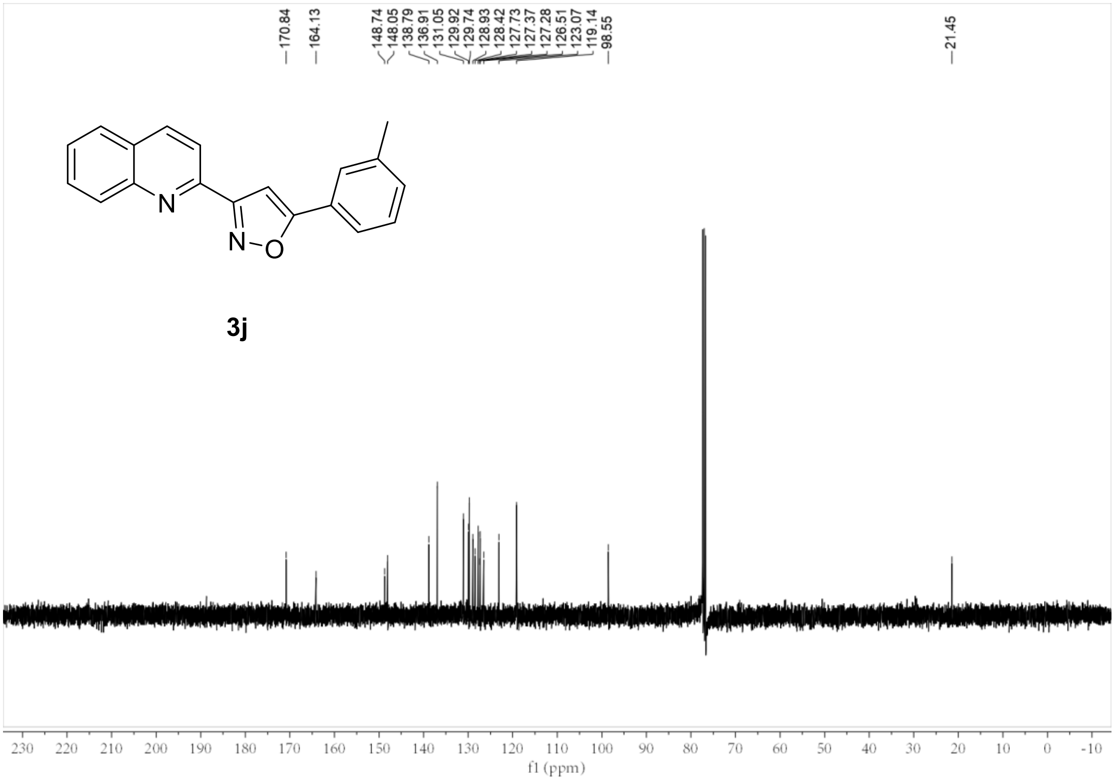

^{1}H NMR spectra of compound **3k**

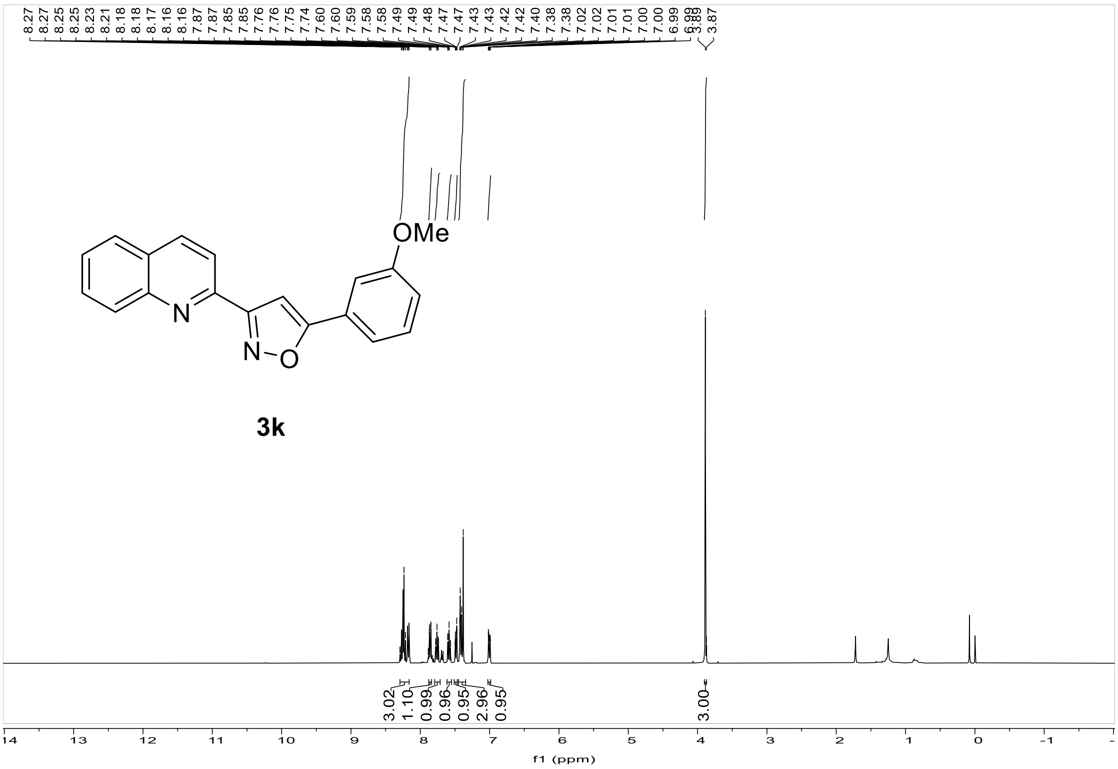

^{13}C{^{1}H} NMR spectra of compound **3k**

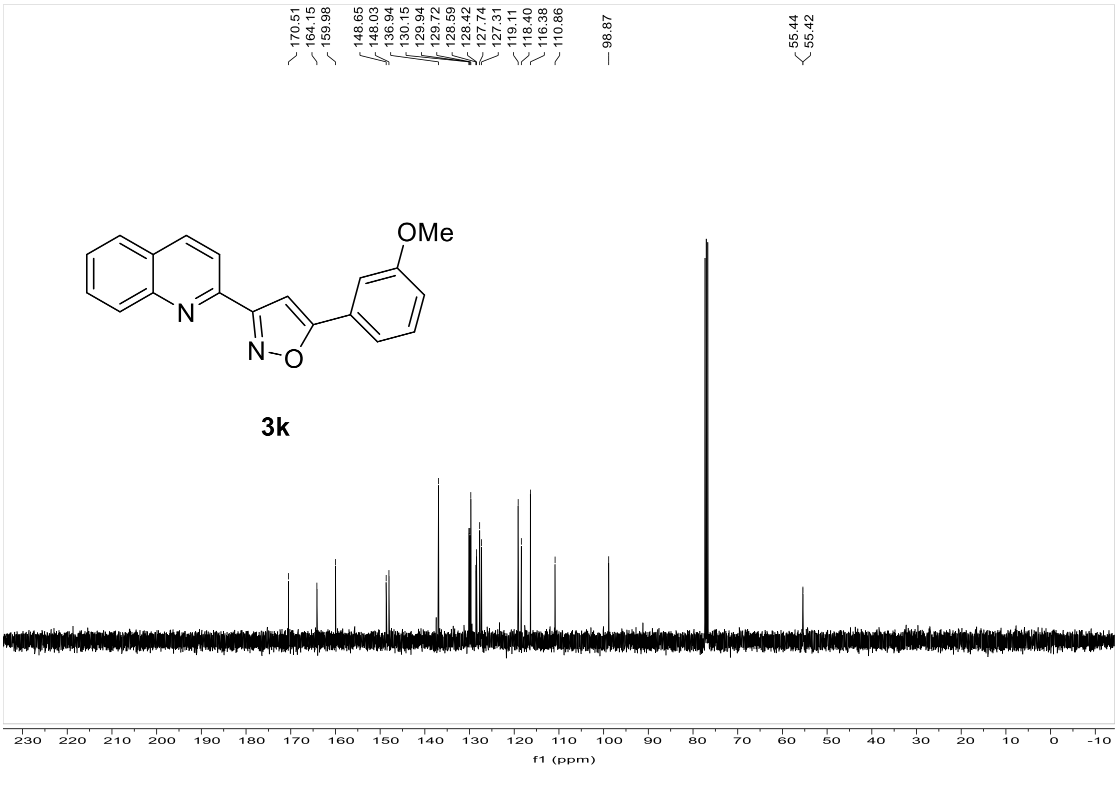

^{1}H NMR spectra of compound **3l**

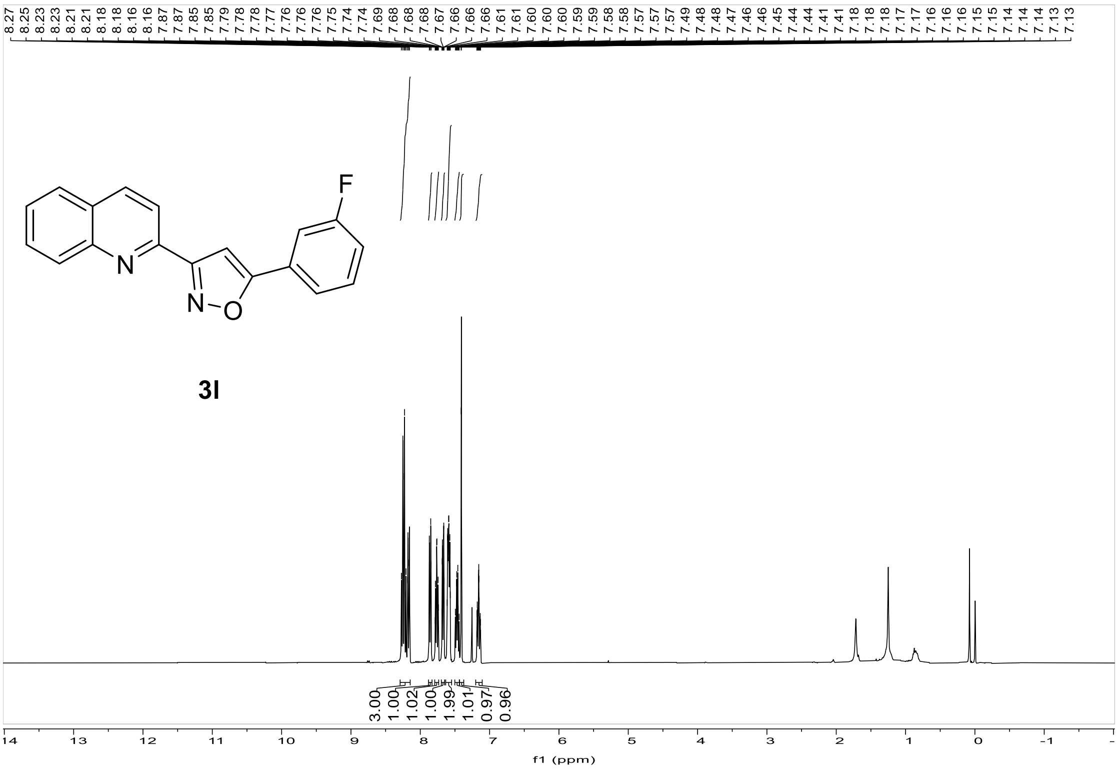

$^{13}C\{^{1}H\}$ NMR spectra of compound **3l**

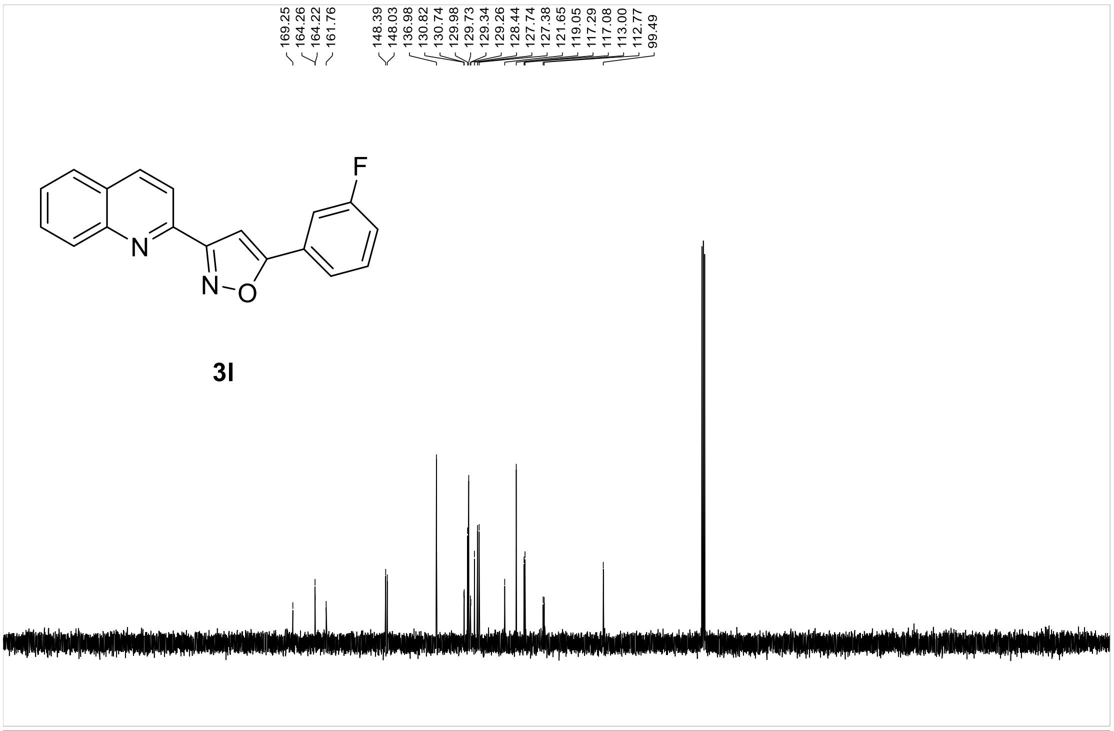

^{19}F NMR spectra of compound 3**l**

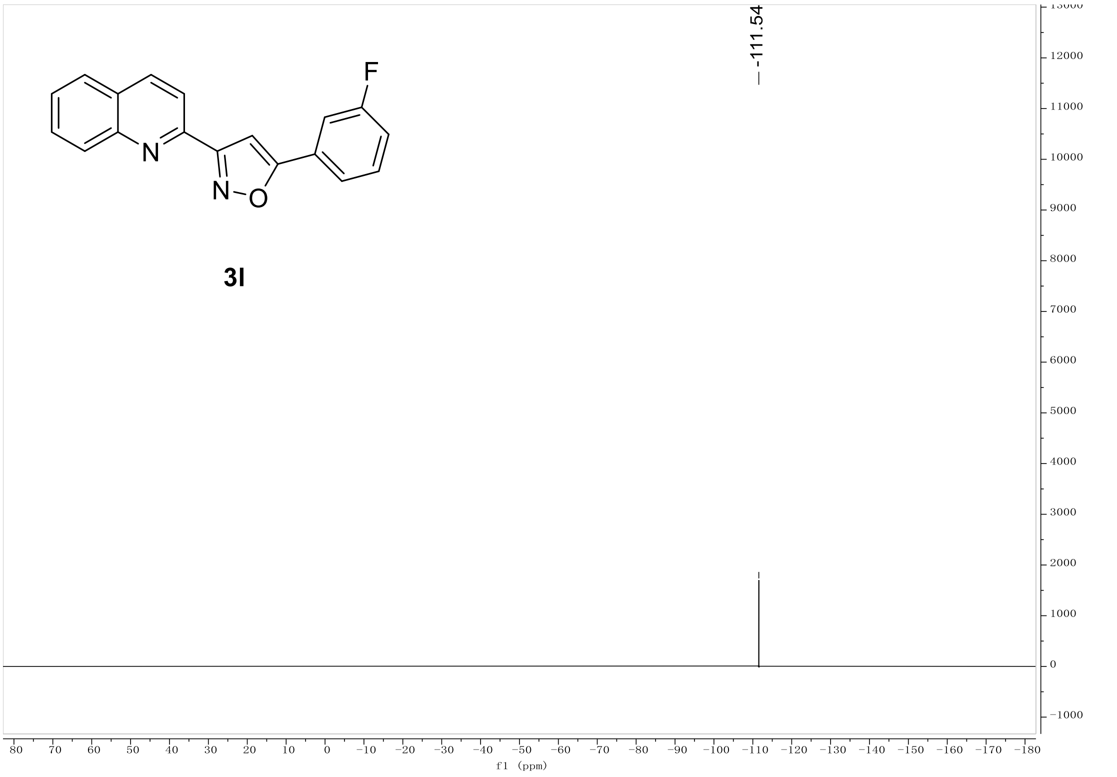

^{1}H NMR spectra of compound **3m**

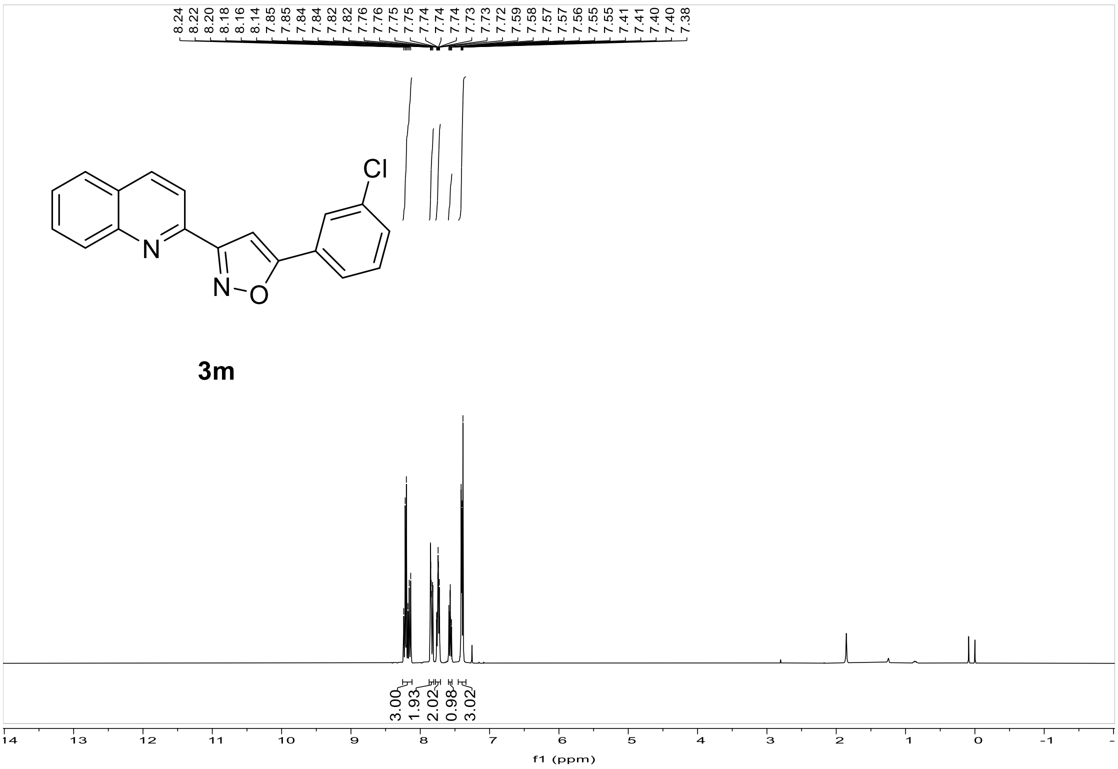

^{13}C{^{1}H} NMR spectra of compound **3m**

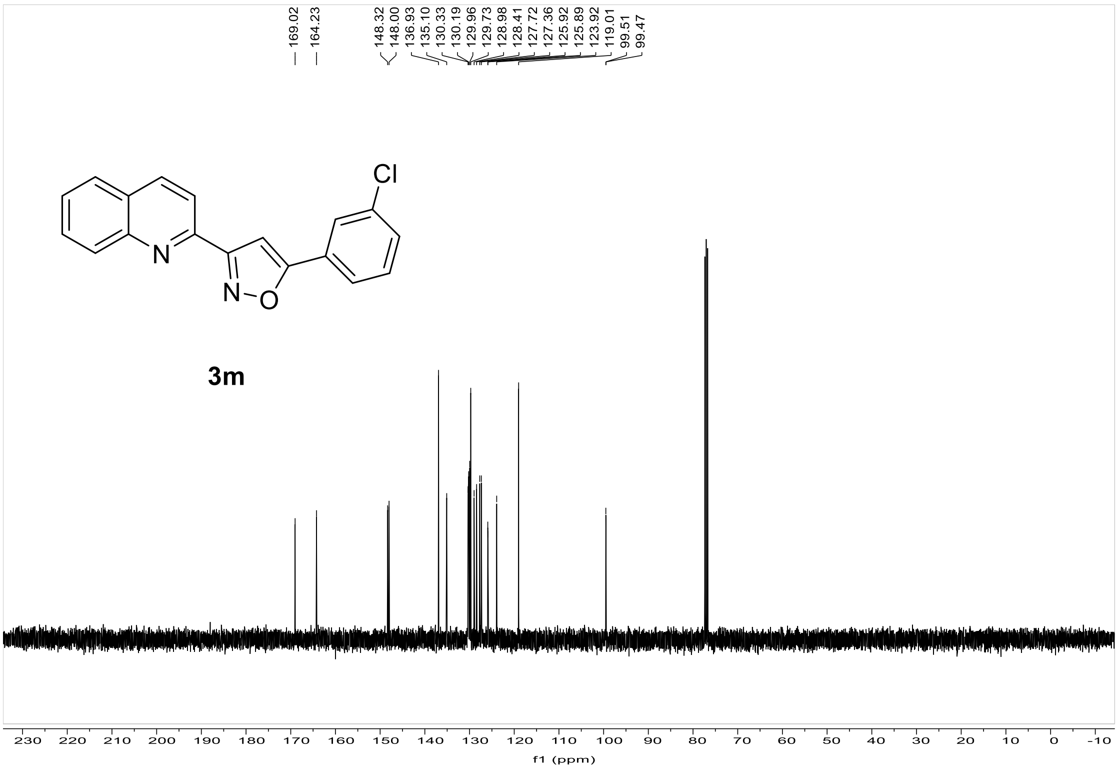

^{1}H NMR spectra of compound **3n**

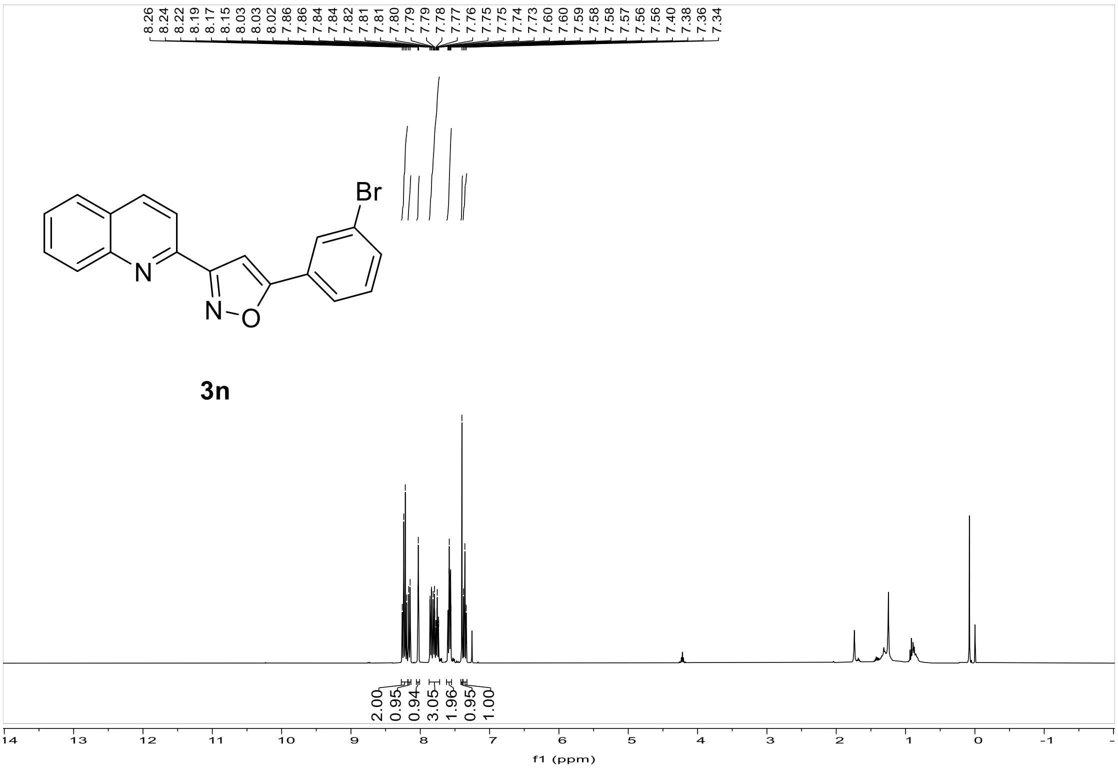

^{13}C{^{1}H} NMR spectra of compound **3n**

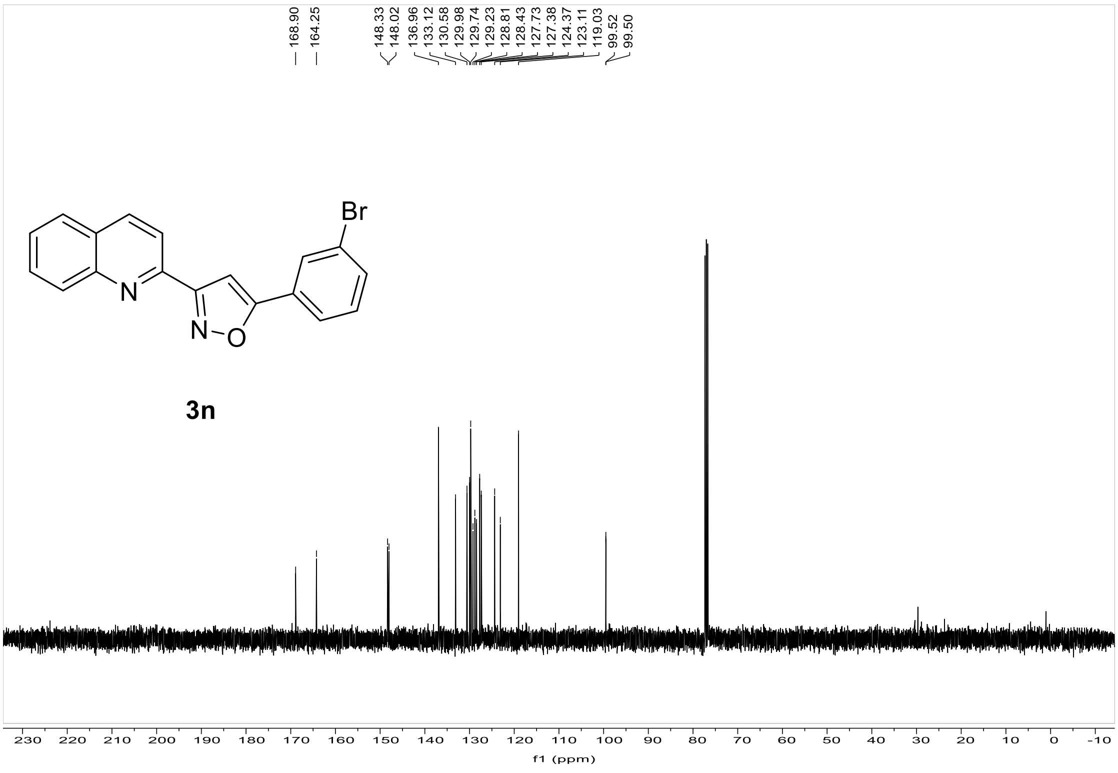

^{1}H NMR spectra of compound **3o**

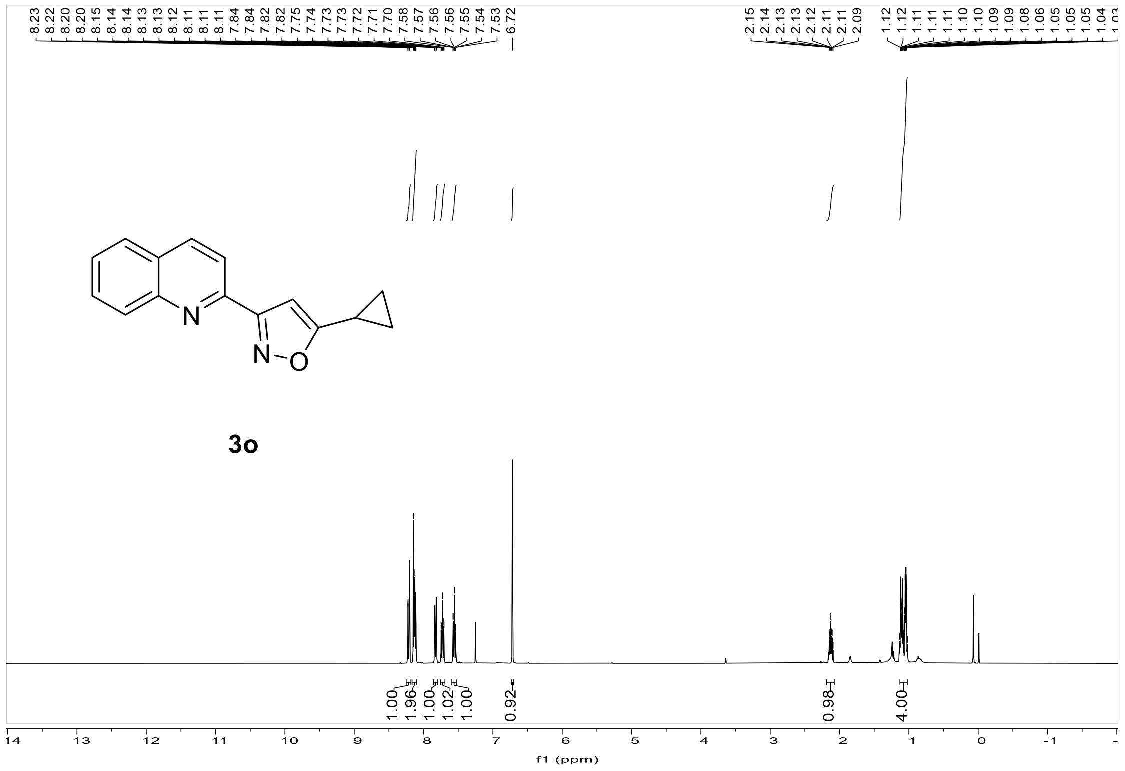

$^{13}C\{^{1}H\}$ NMR spectra of compound **3o**

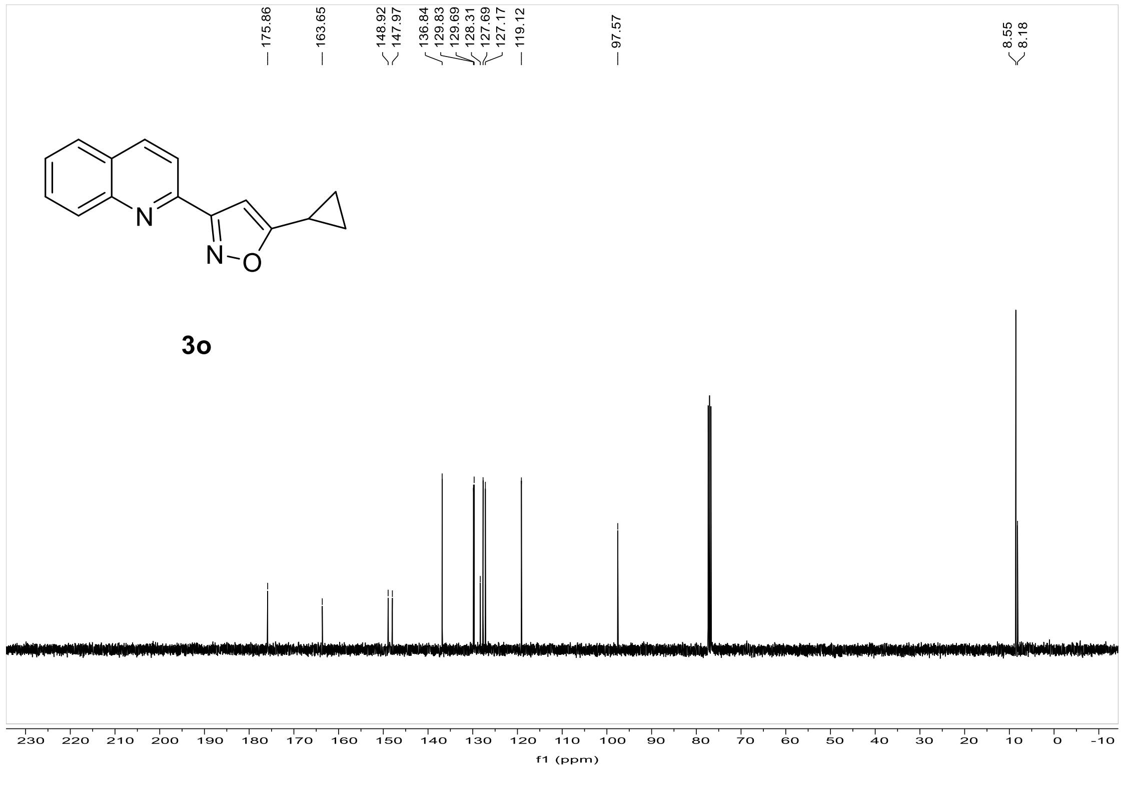

^{1}H NMR spectra of compound **3p**

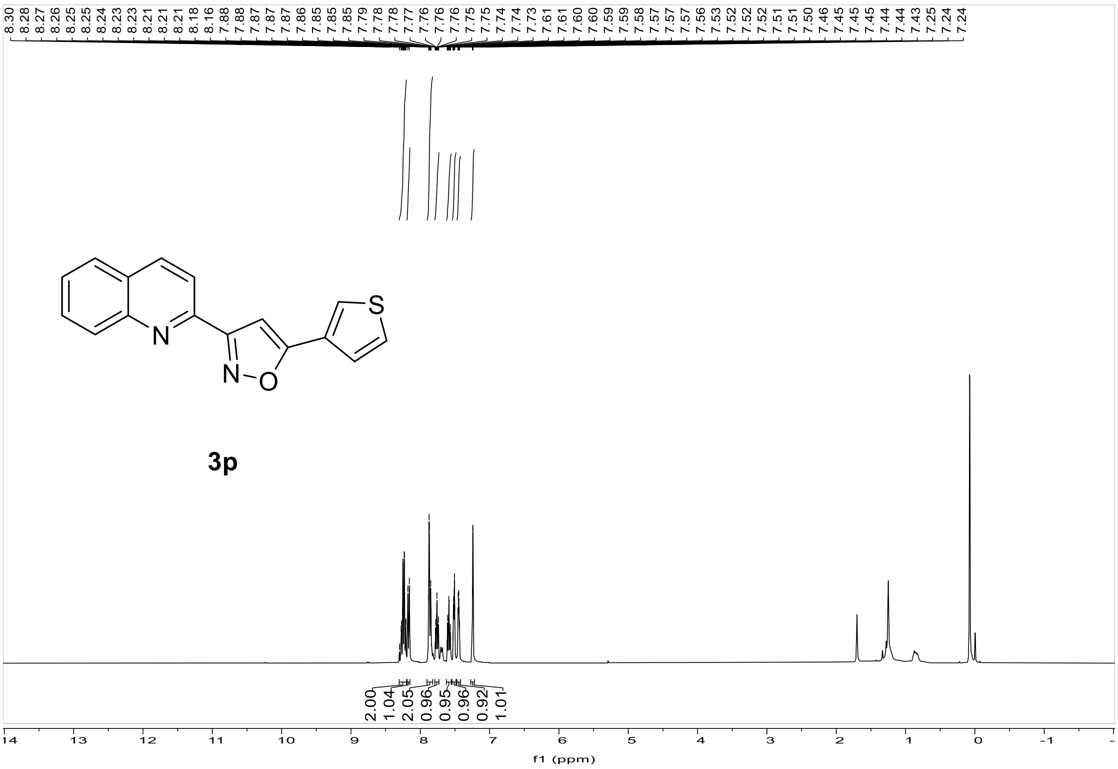

^{13}C{^{1}H} NMR spectra of compound **3p**

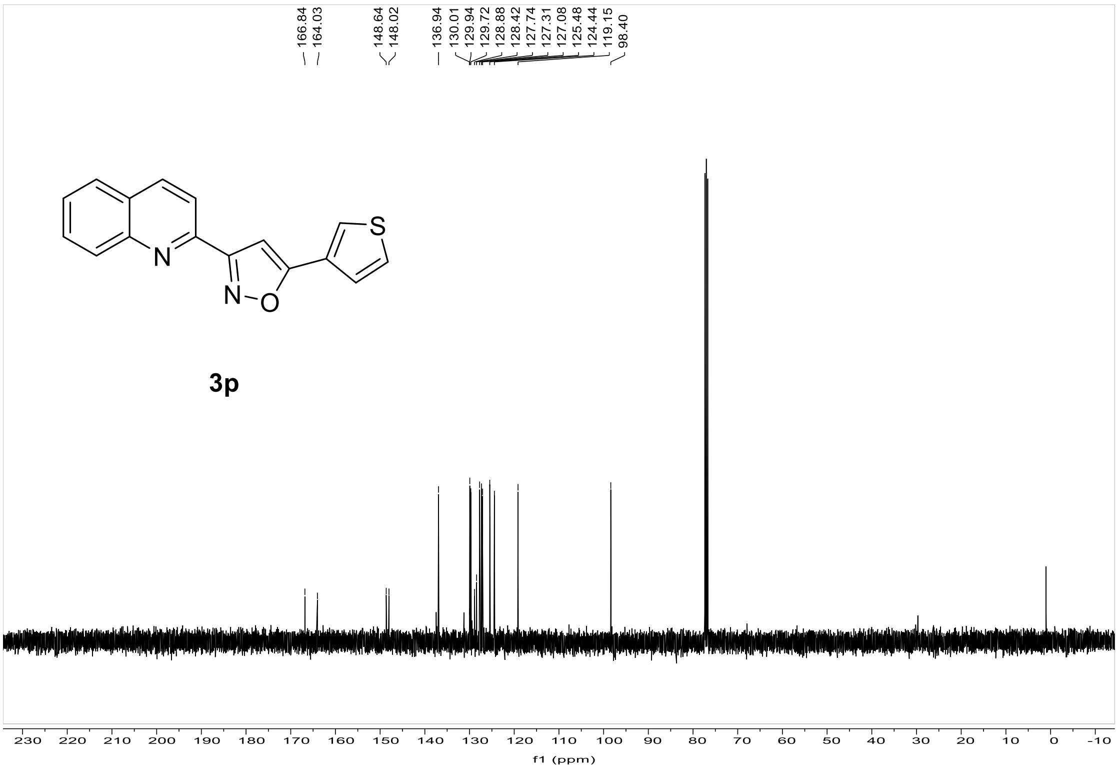

^{1}H NMR spectra of compound **4a**

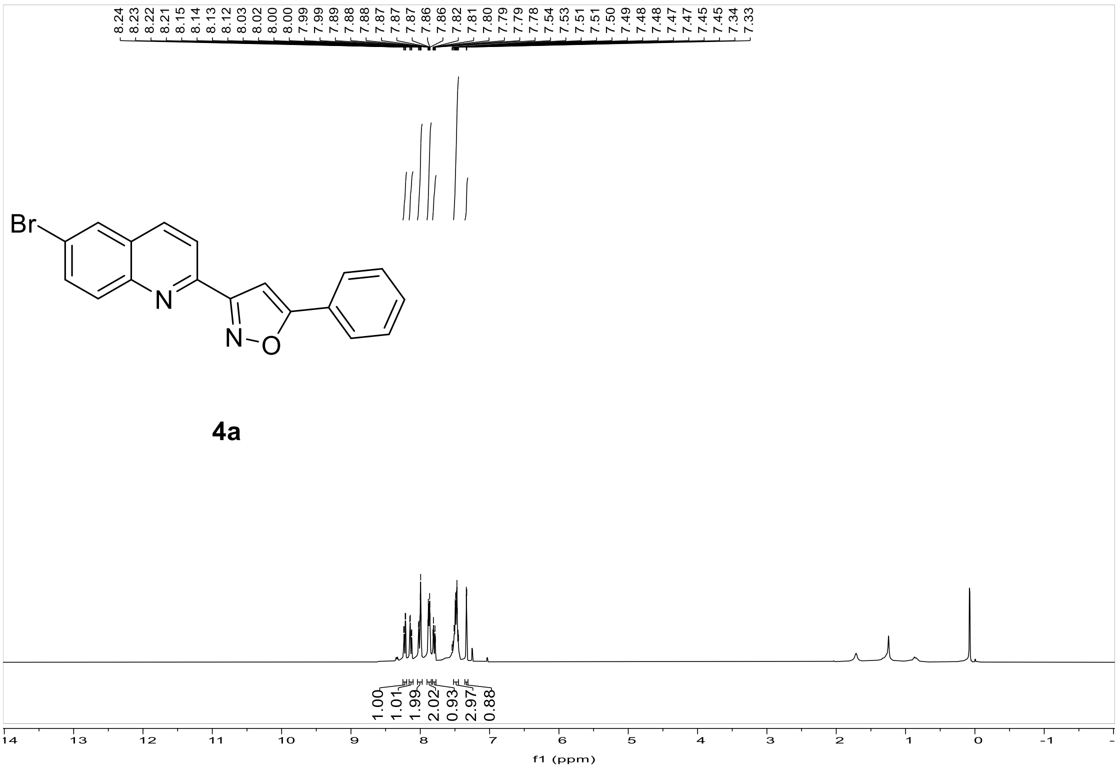

^{13}C{^{1}H} NMR spectra of compound **4a**

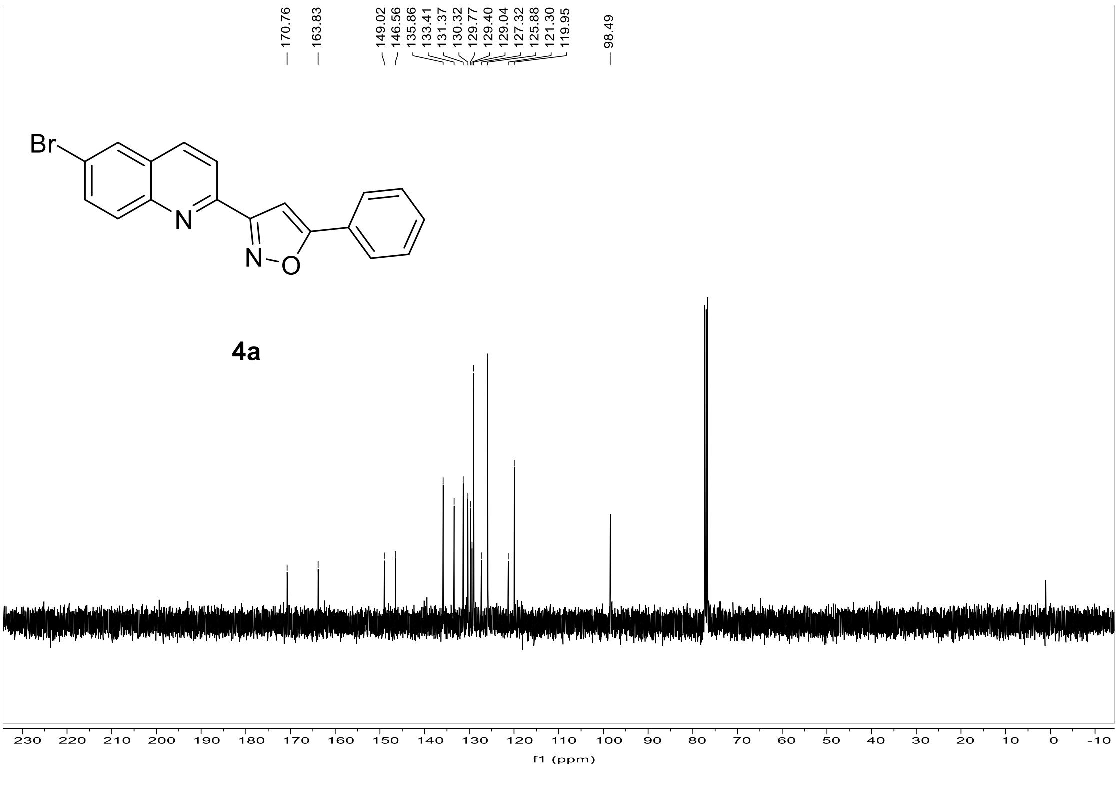

^{1}H NMR spectra of compound **4b**

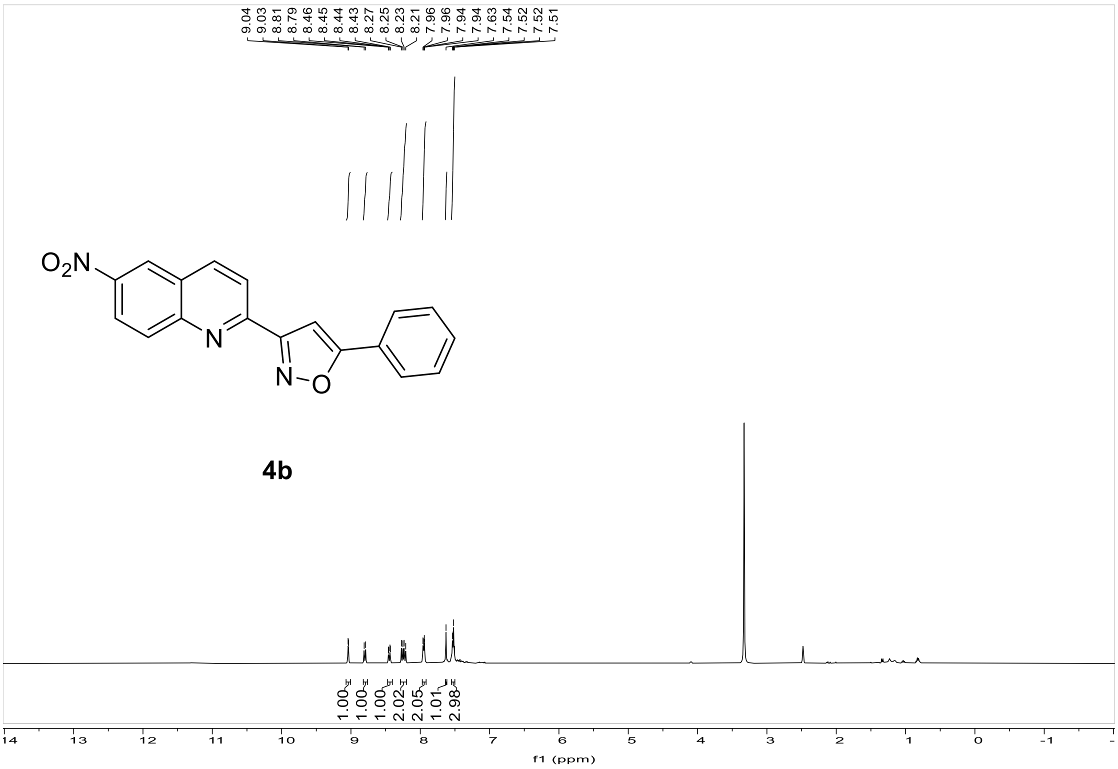

^{13}C{^{1}H} NMR spectra of compound **4b**

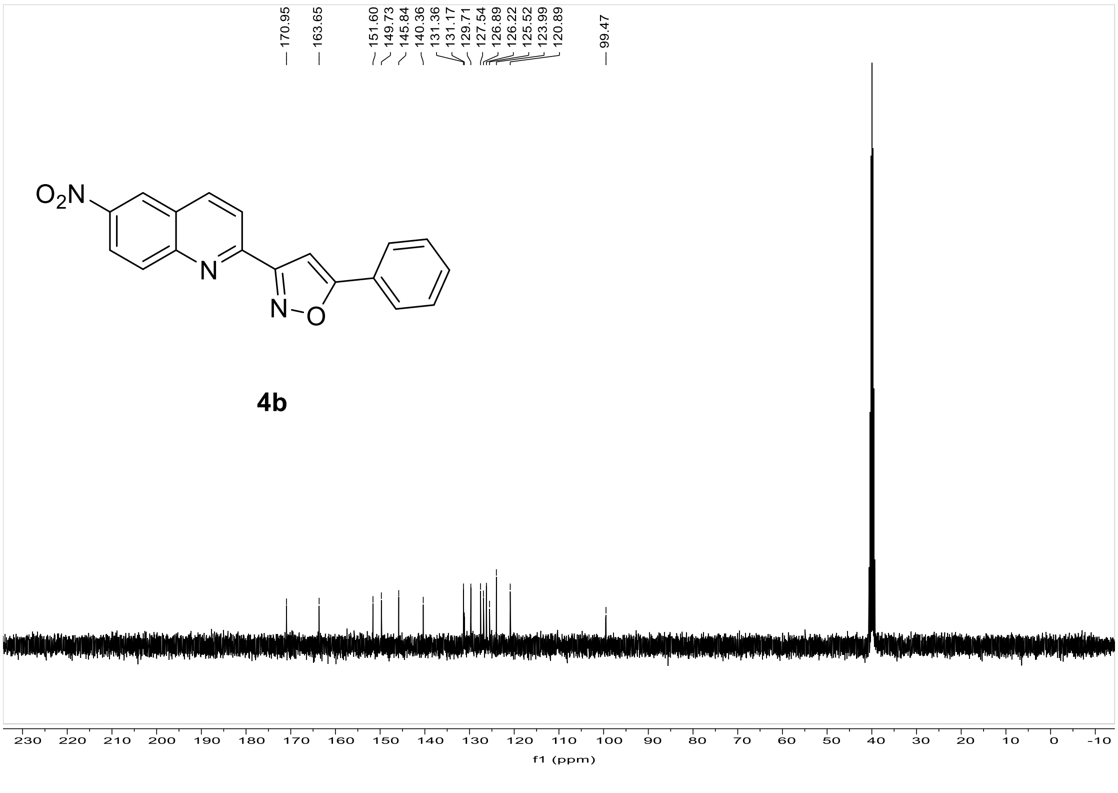

^{1}H NMR spectra of compound **4c**

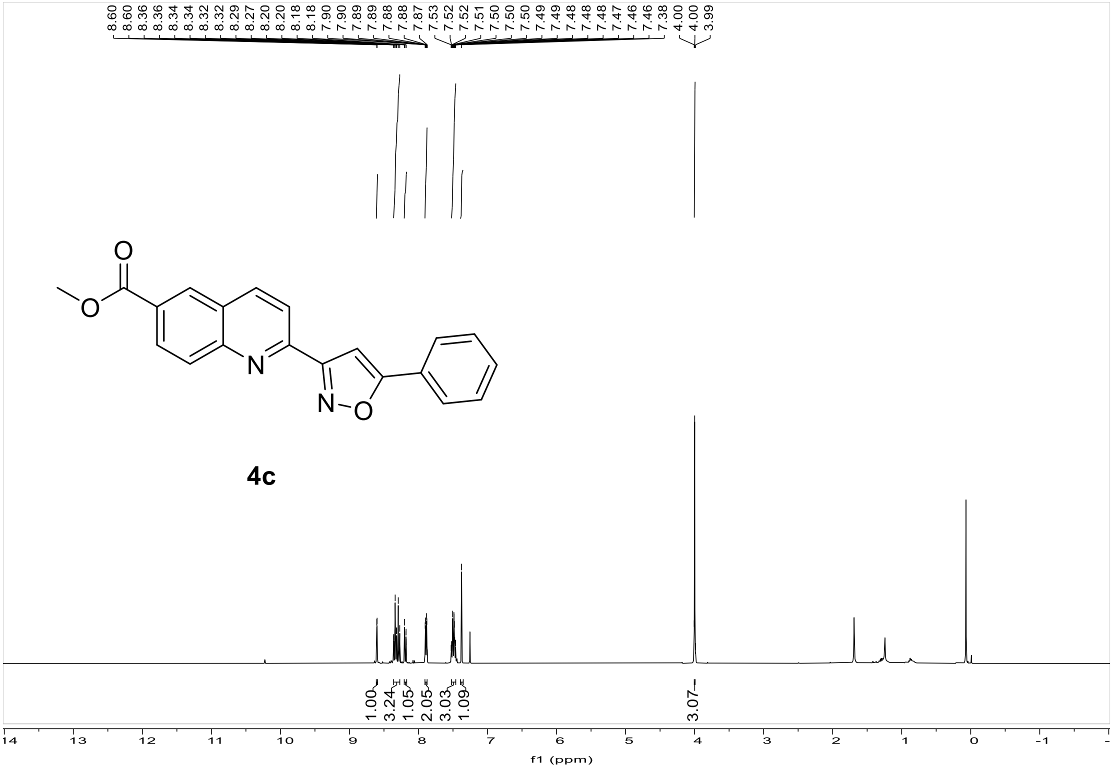

^{13}C{^{1}H} NMR spectra of compound **4c**

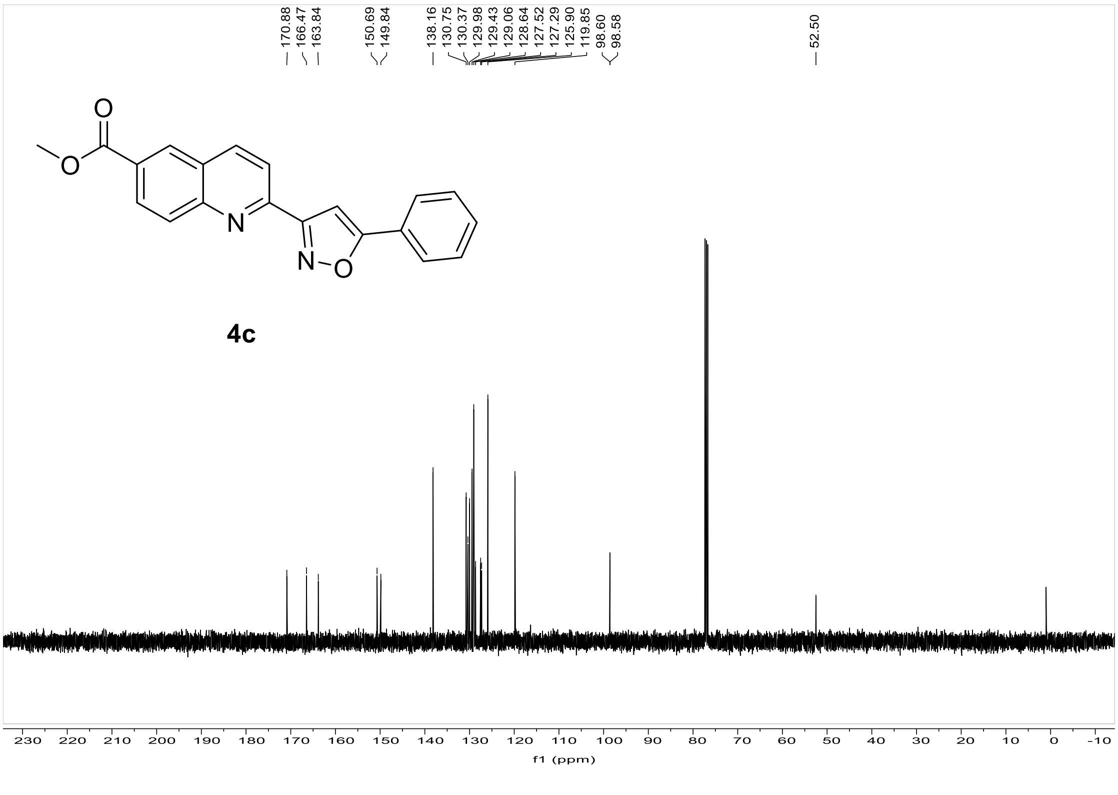

^{1}H NMR spectra of compound **4d**

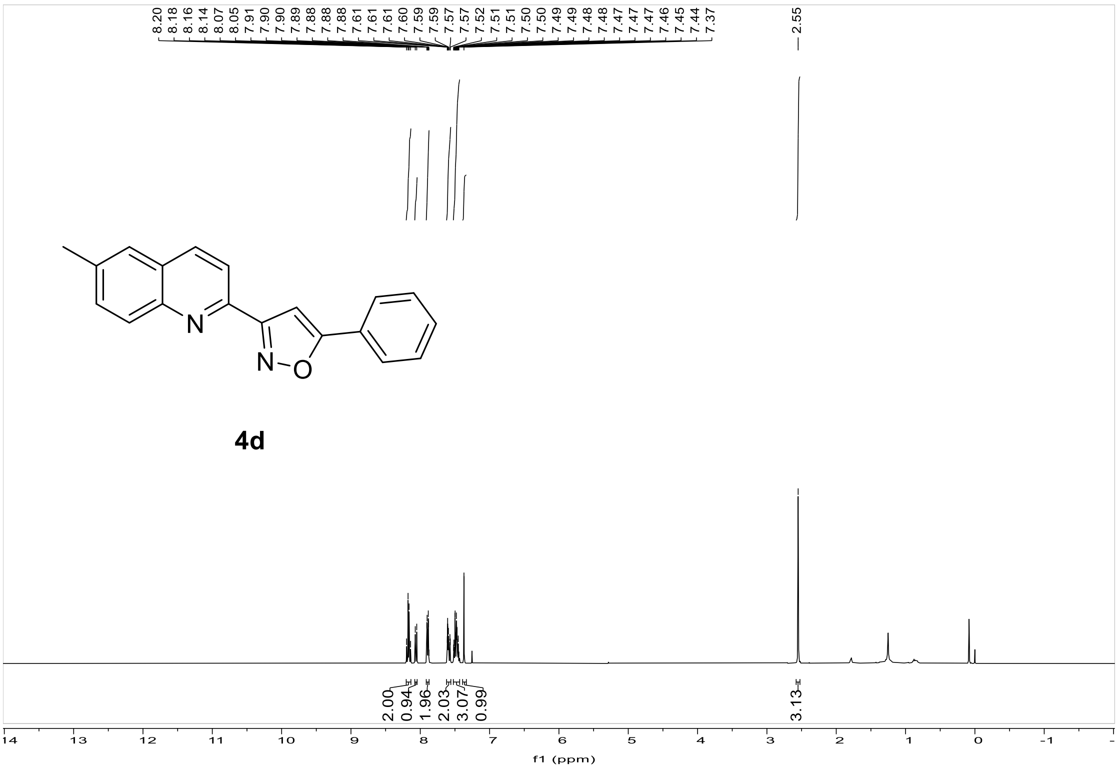

^{13}C{^{1}H} NMR spectra of compound **4d**

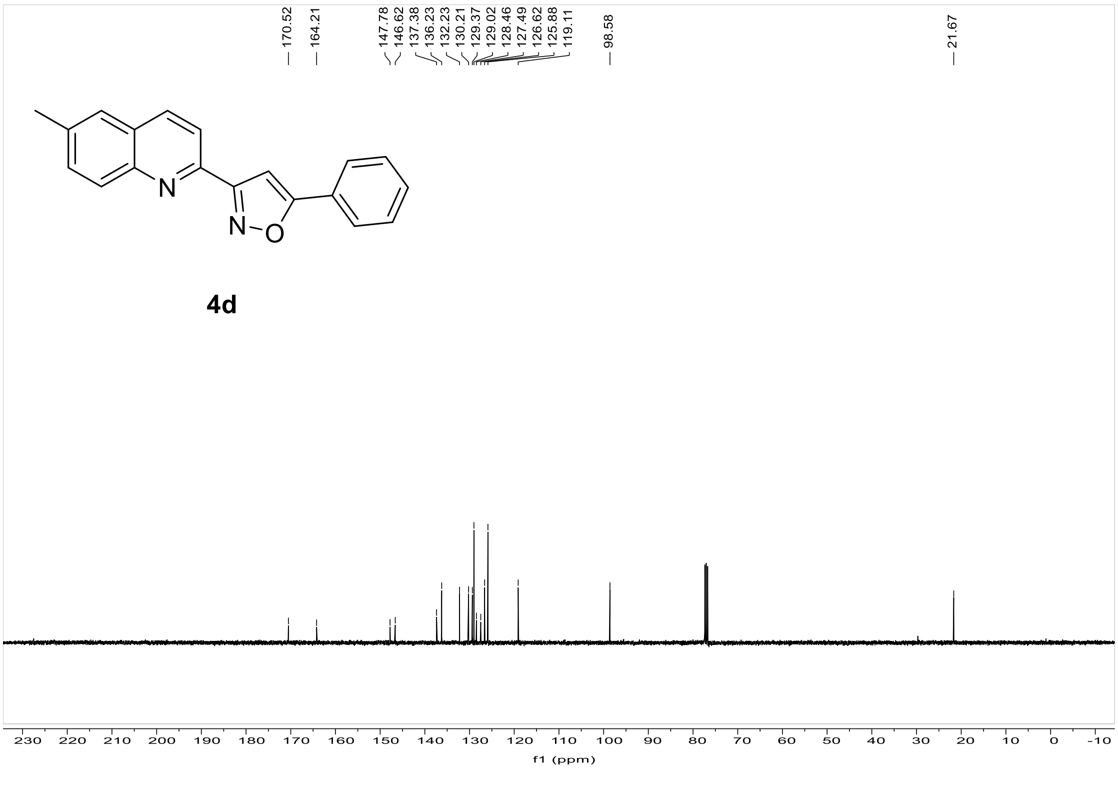

^{1}H NMR spectra of compound **4e**

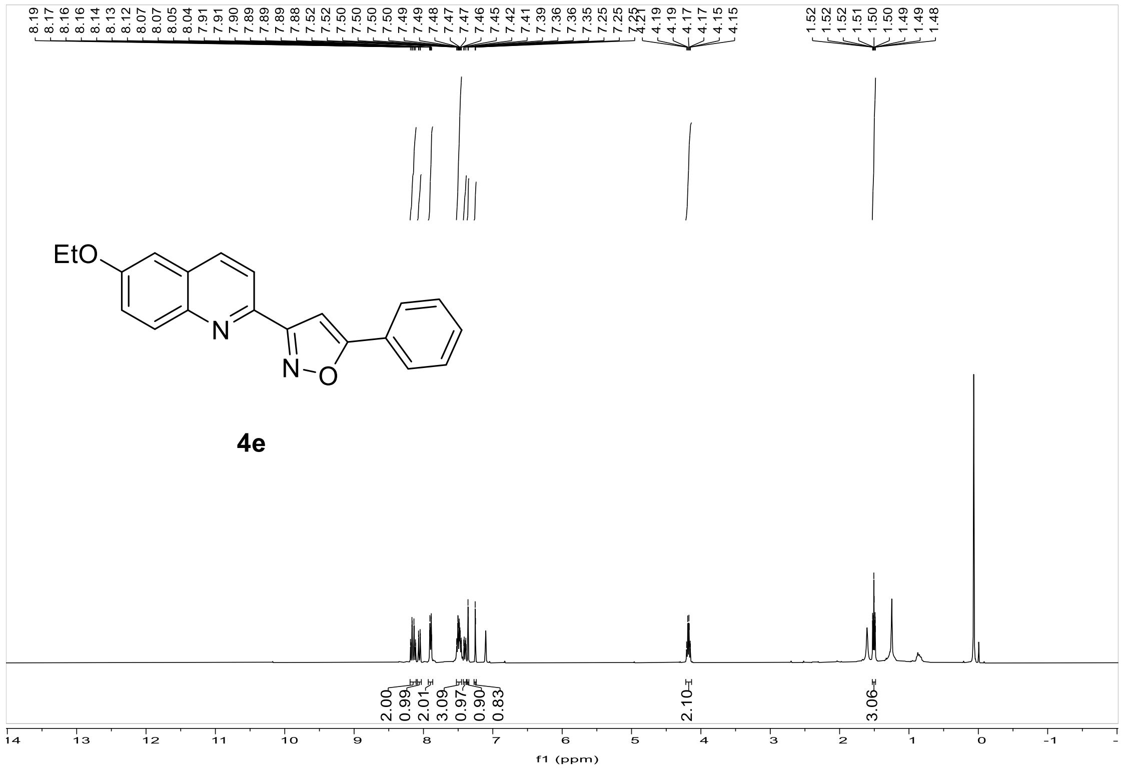

$^{13}C\{^{1}H\}$ NMR spectra of compound **4e**

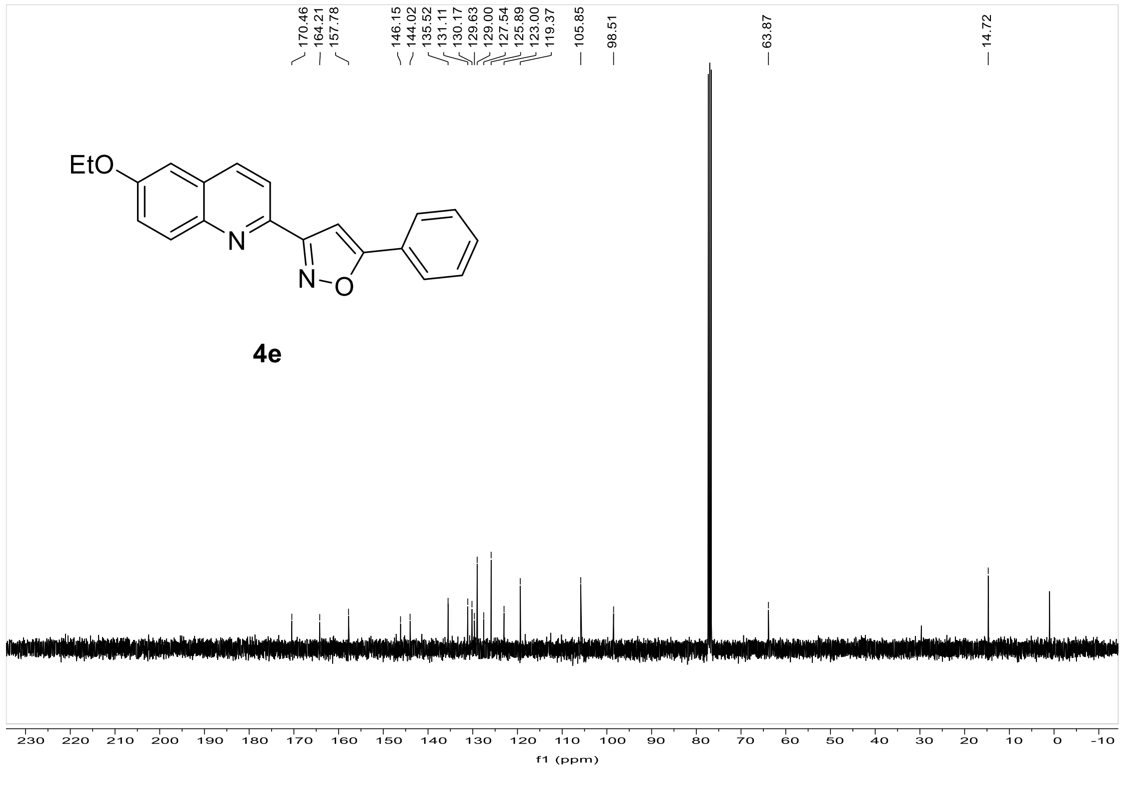

^{1}H NMR spectra of compound **4f**

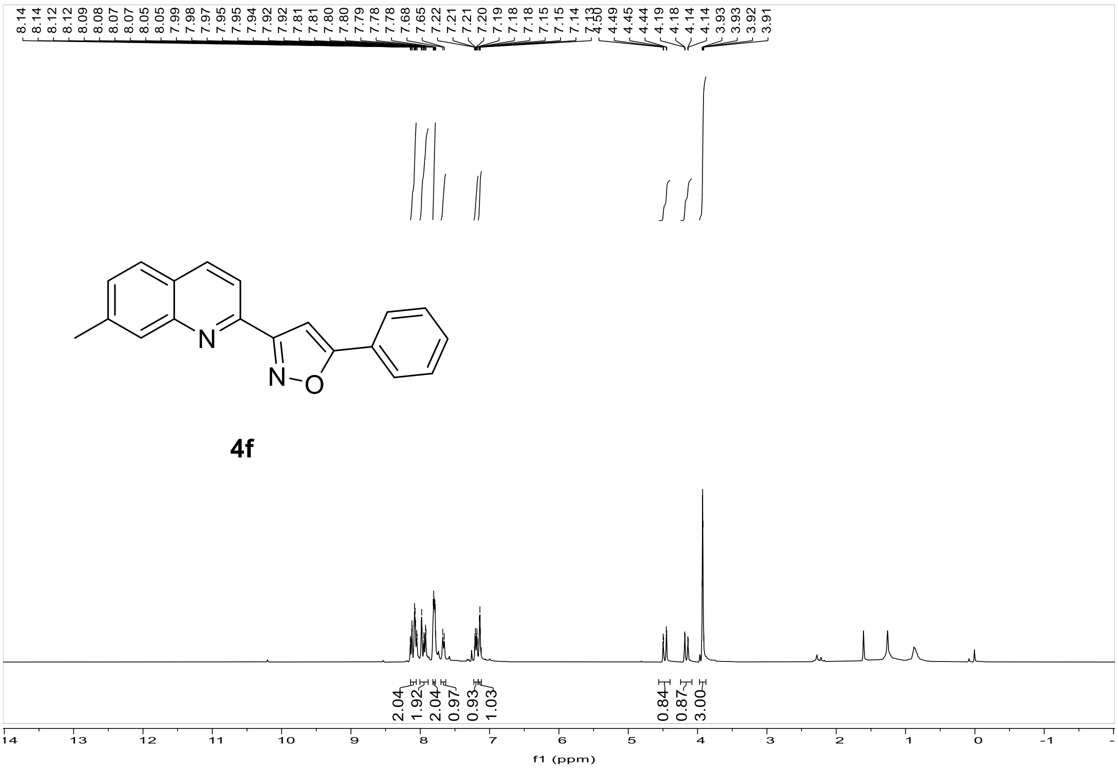

^{13}C{^{1}H} NMR spectra of compound **4f**

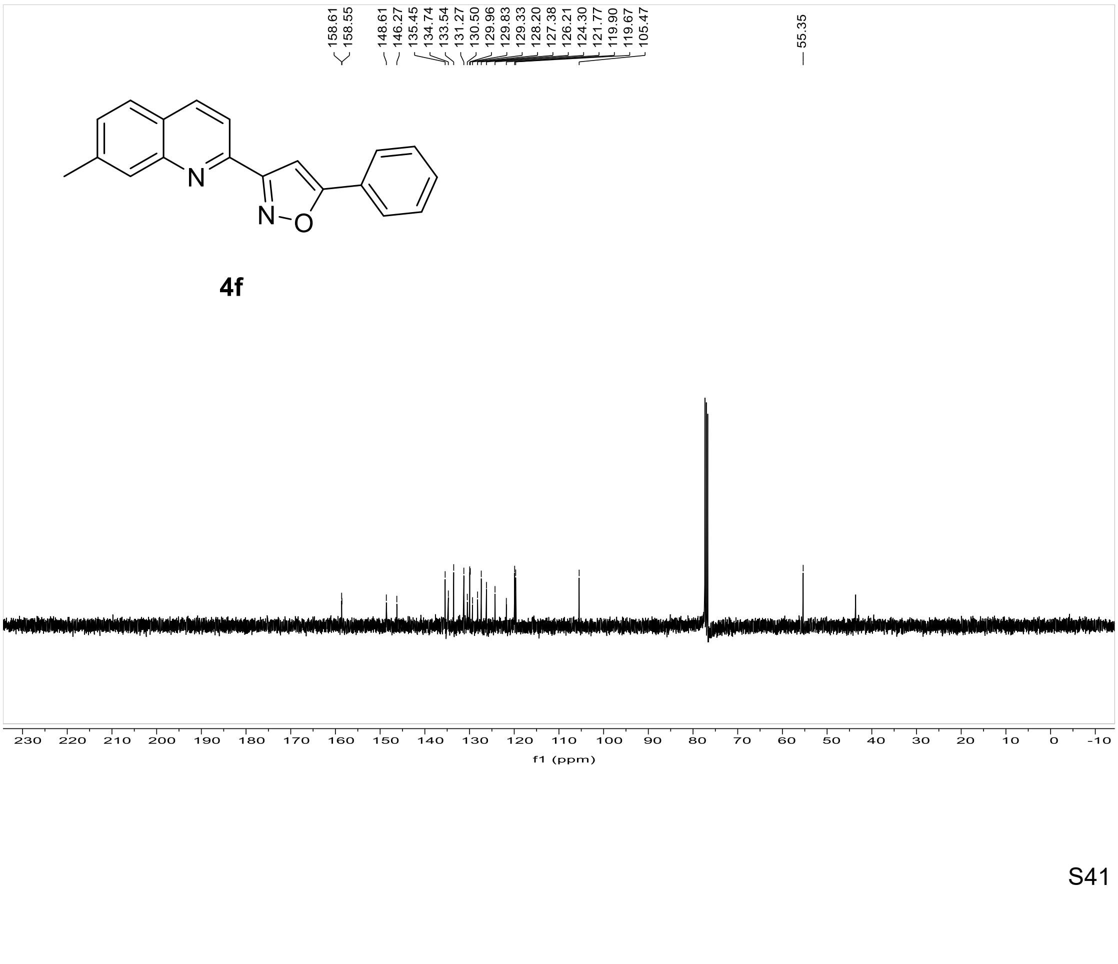

^{1}H NMR spectra of compound **4g**

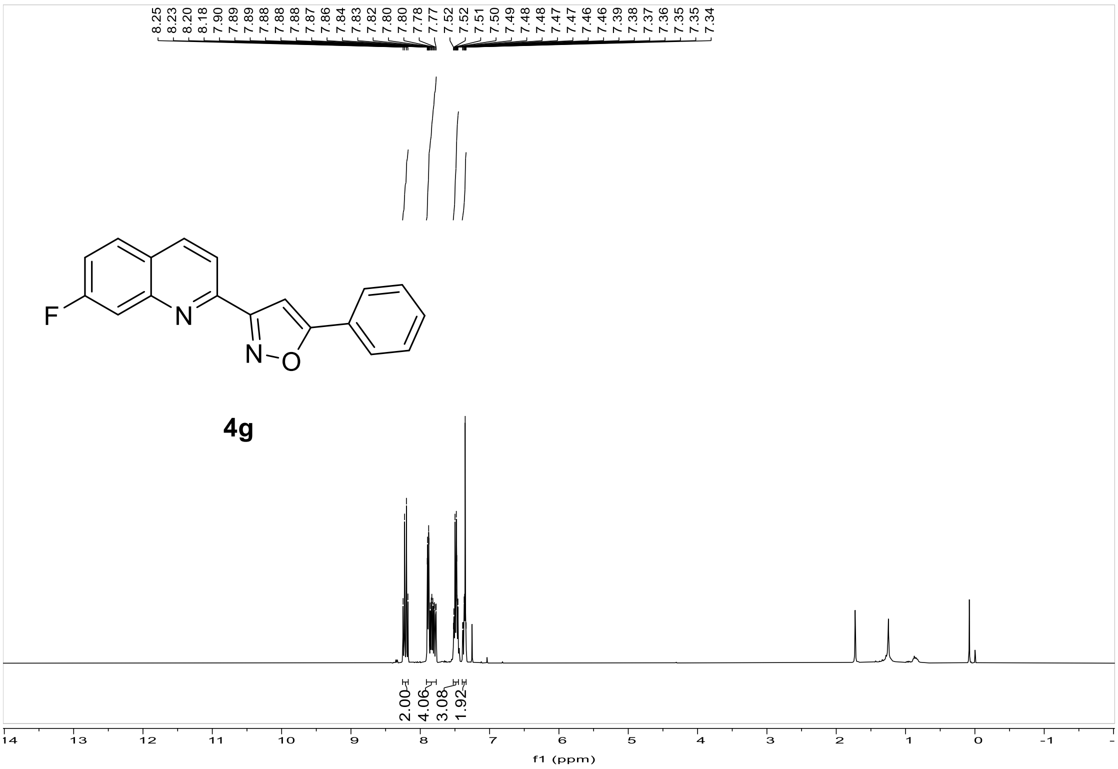

^{13}C{^{1}H} NMR spectra of compound **4g**

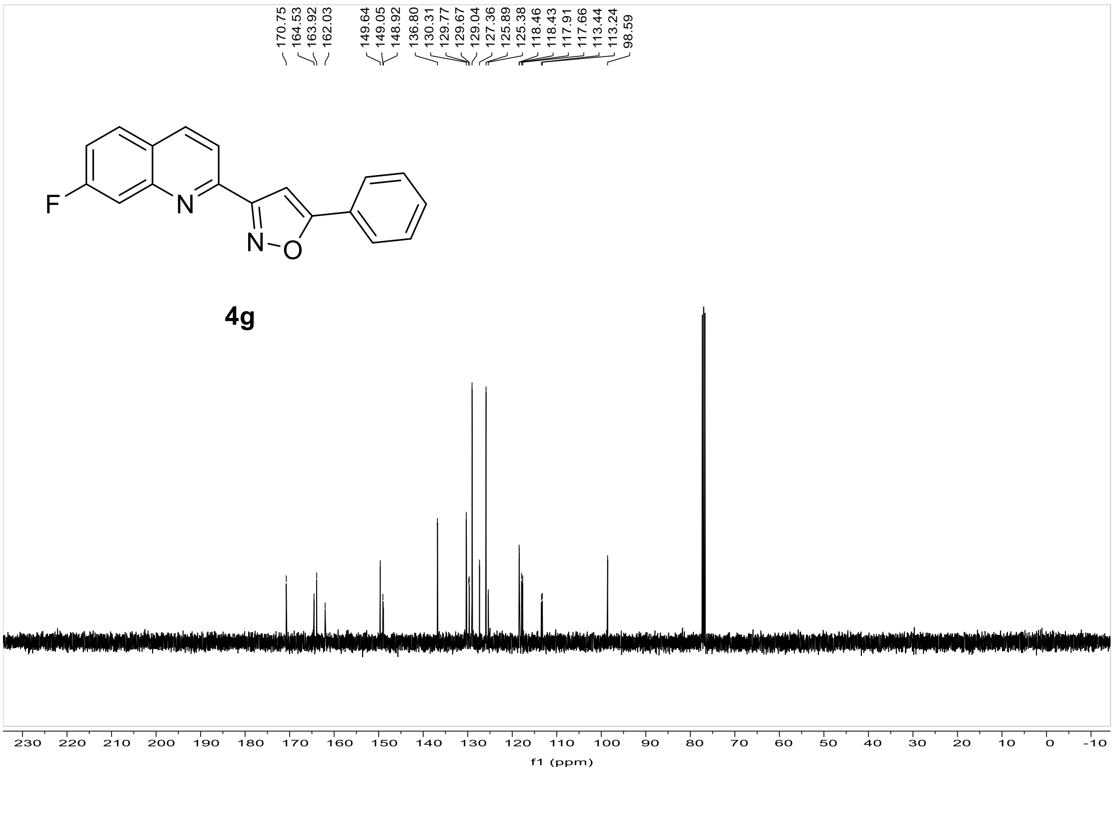

^{19}F NMR spectra of compound **4g**

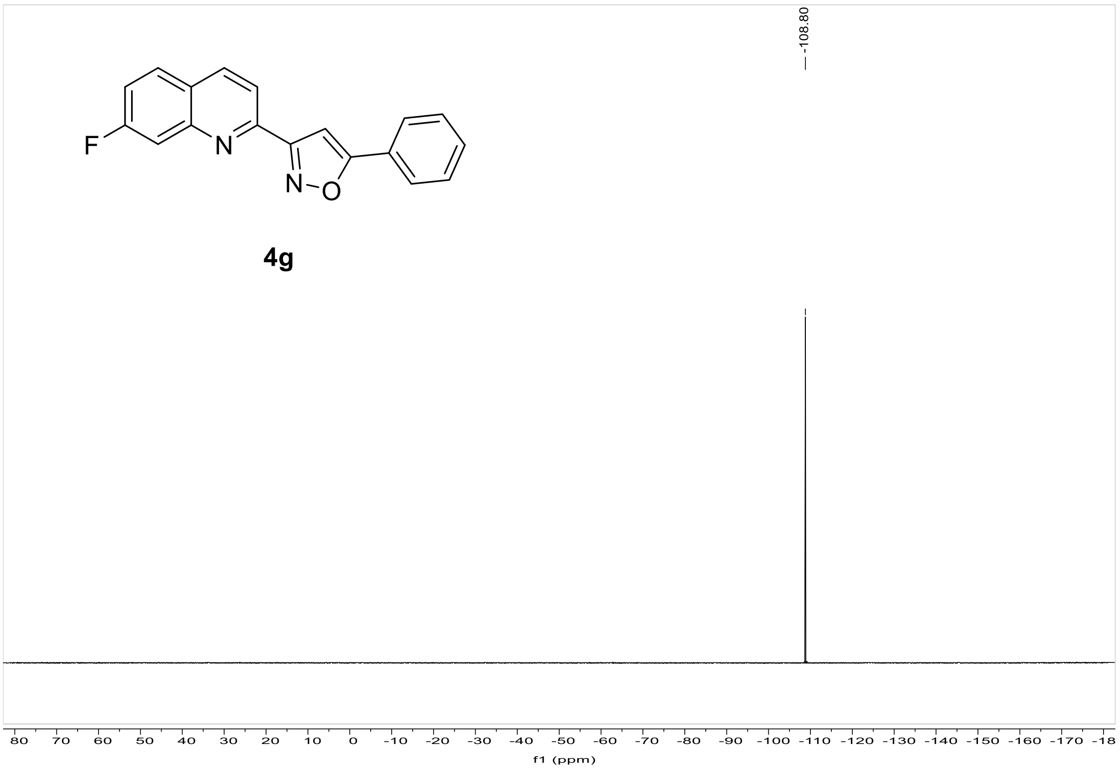

^{1}H NMR spectra of compound **4h**

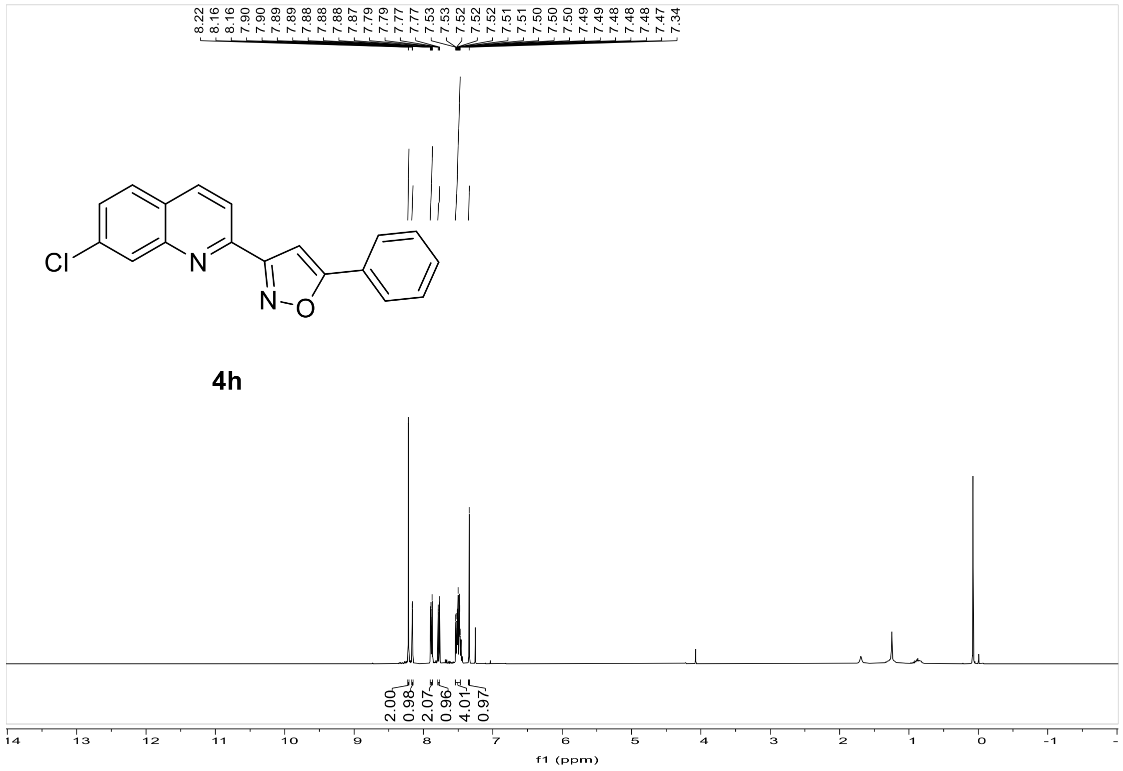

^{13}C{^{1}H} NMR spectra of compound **4h**

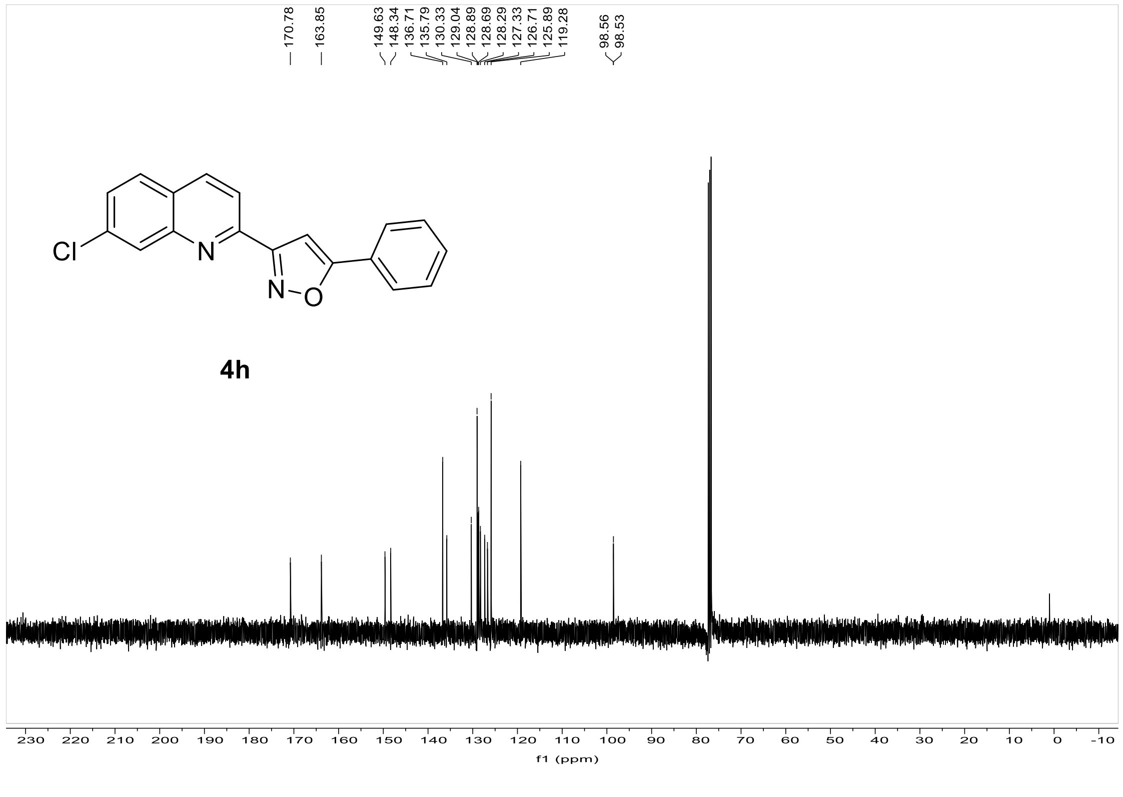

^{1}H NMR spectra of compound **4i**

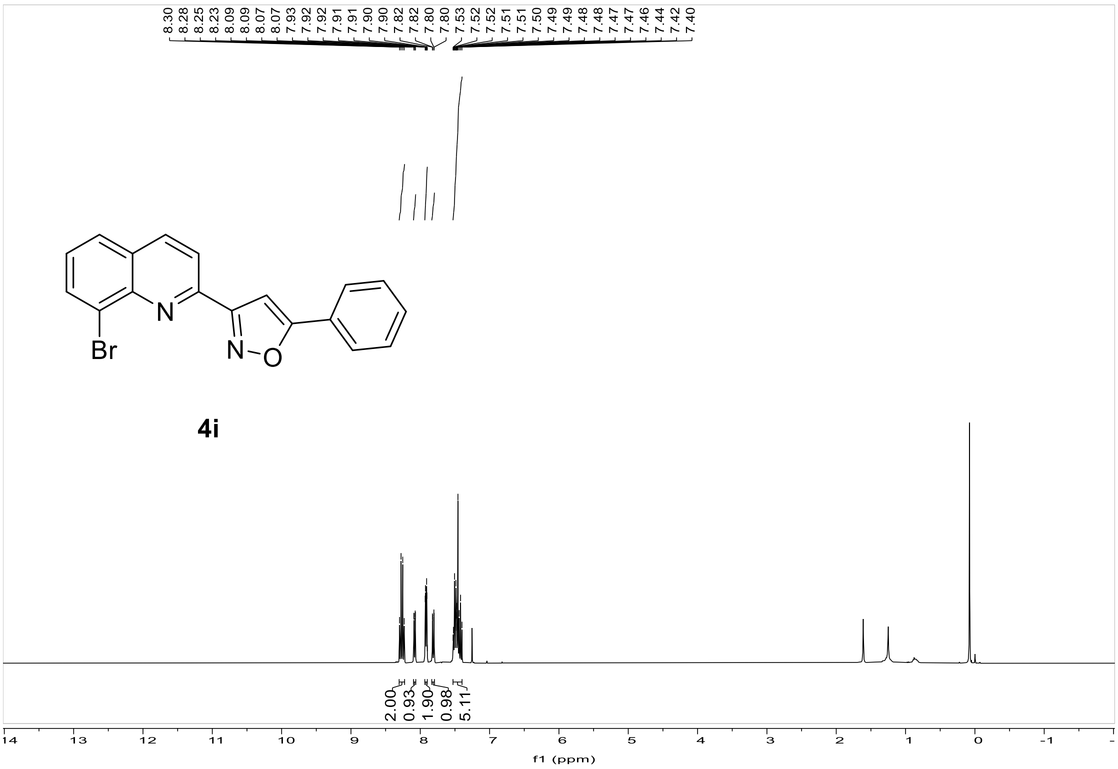

^{13}C{^{1}H} NMR spectra of compound **4i**

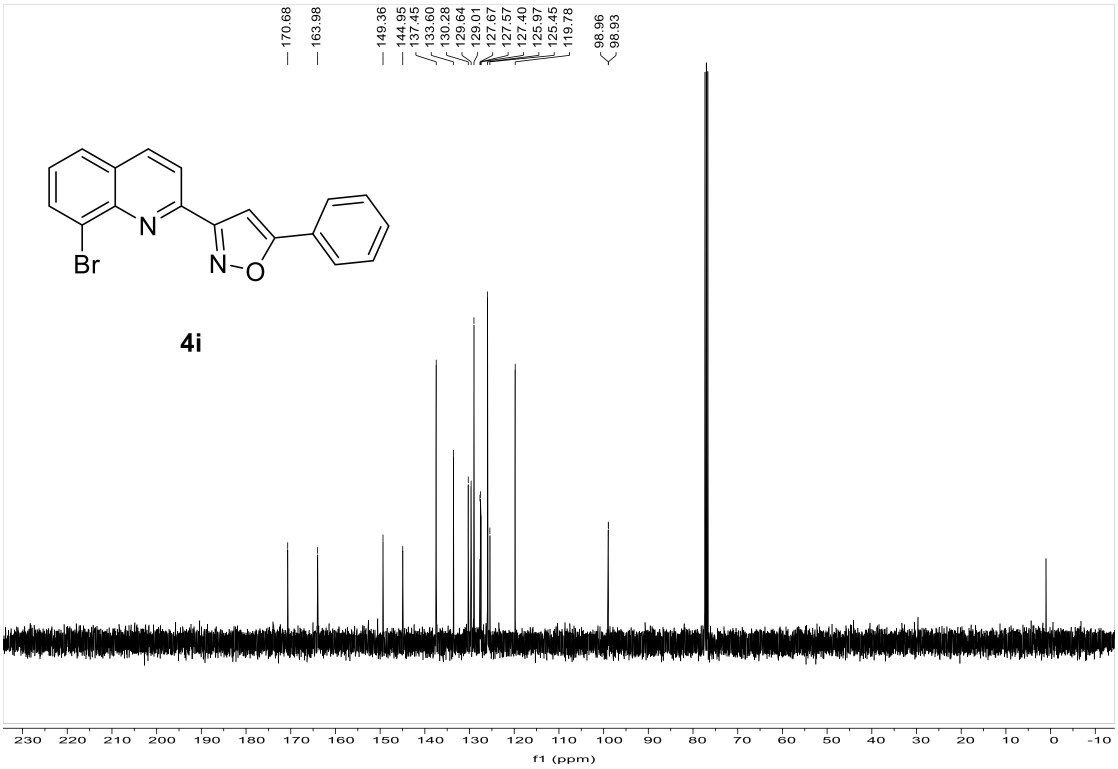

^{1}H NMR spectra of compound **4j**

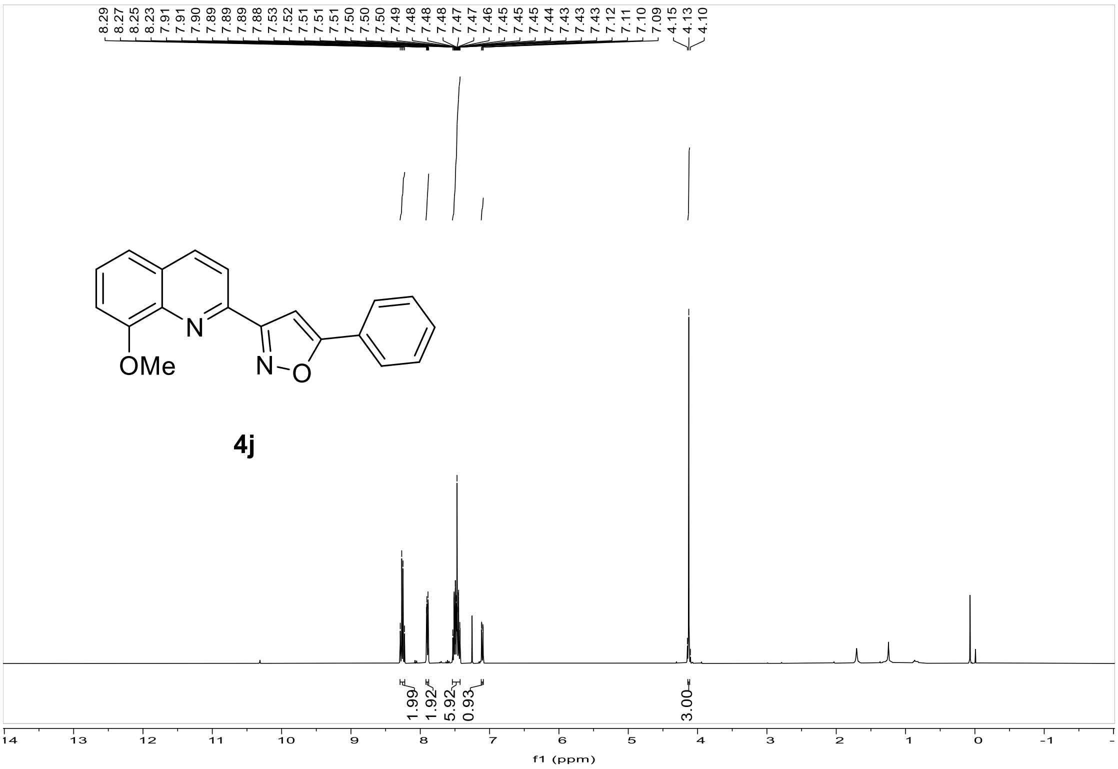

^{13}C{^{1}H} NMR spectra of compound **4j**

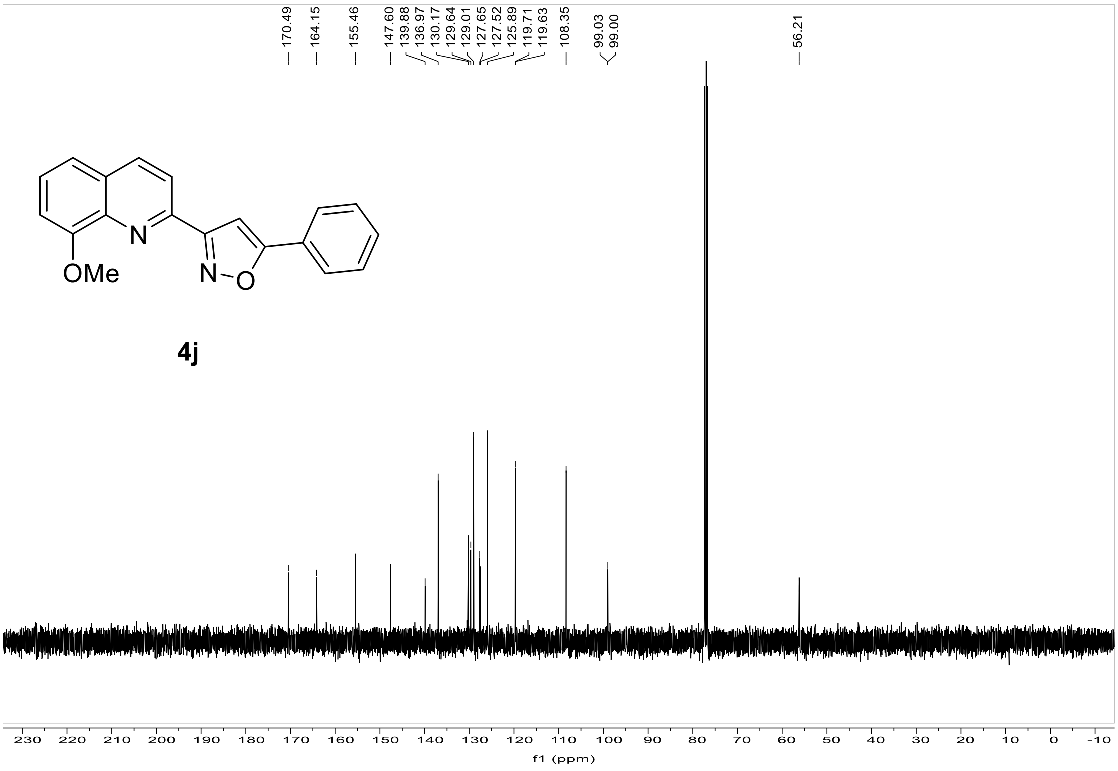

^{1}H NMR spectra of compound **4k**

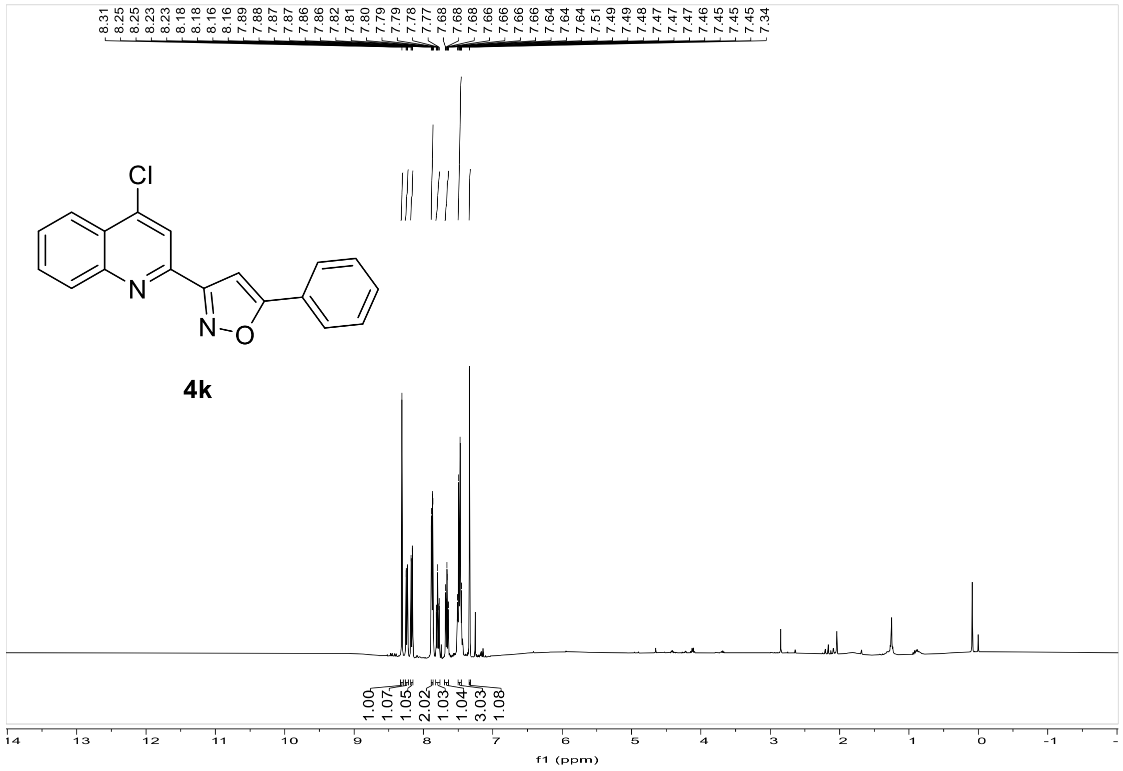

^{13}C{^{1}H} NMR spectra of compound **4k**

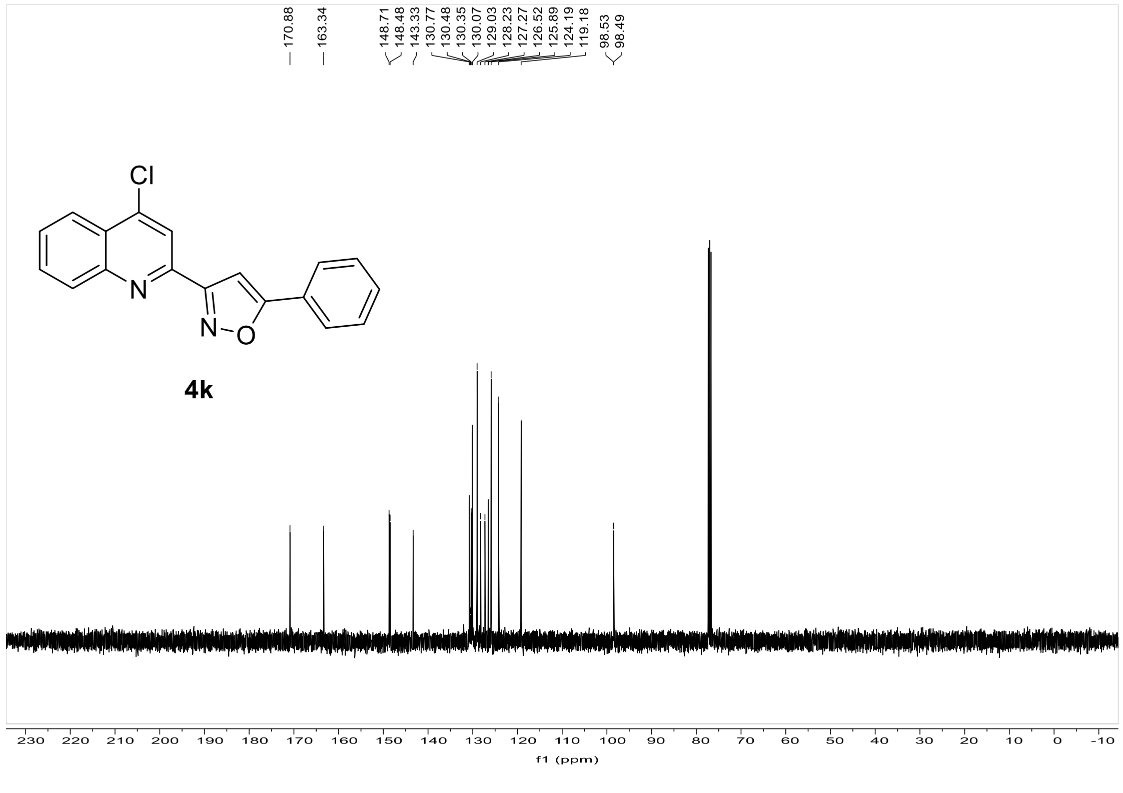

^{1}H NMR spectra of compound **4l**

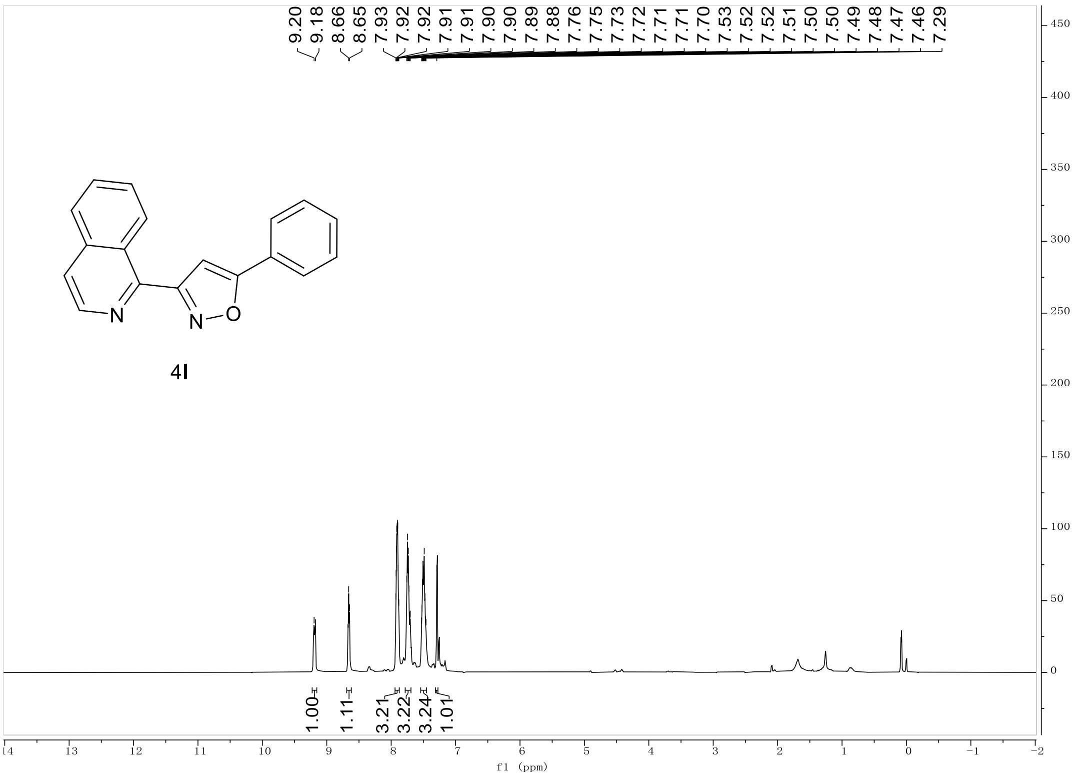

^{13}C{^{1}H} NMR spectra of compound **4l**

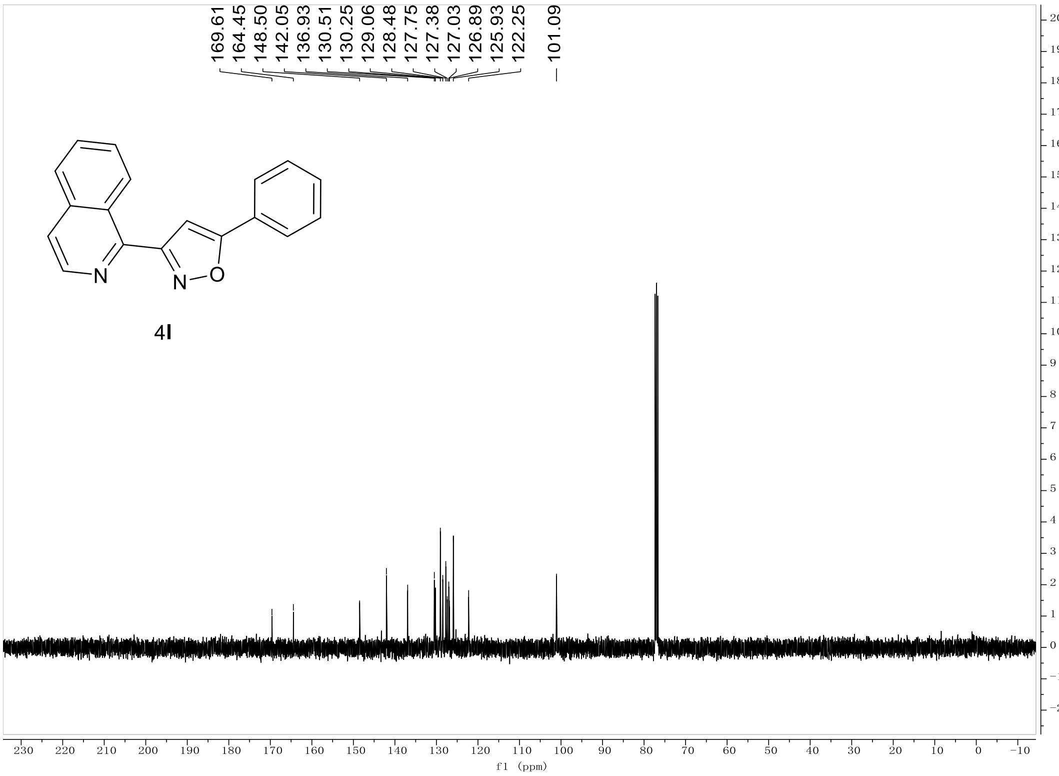

Supplement: File 1 — Characterization data and copies of spectra. [file Beilstein_J_Org_Chem-19-1562-s001.pdf]
